# Genetic Structure of the Perennial Flax Trifecta: *Linum austriacum*, *L. lewisii*, *L. perenne*

**DOI:** 10.3390/genes17080978

**Published:** 2026-08-20

**Authors:** Hannah J. Hall, Neil O. Anderson, Donald L. Wyse, Kevin J. Betts

**Affiliations:** 1 Department of Horticultural Science, University of Minnesota, St. Paul, MN 55108, USA; halljhannah@gmail.com; 2 Department of Agronomy & Plant Genetics, University of Minnesota, St. Paul, MN 55108, USAbetts003@umn.edu (K.J.B.)

**Keywords:** genotype by sequencing, single-nucleotide polymorphism, oilseed, fiber (linen), herbaceous perennial, cut flower

## Abstract

**Background/Objectives**. Annual flaxseed (*Linum usitatissimum*) is the most cultivated *Linum* species. Perennial species (*Linum austriacum*, *Linum lewisii*, *Linum perenne*) show potential as alternative oilseed and fiber sources. It is critical to determine genetic variation in any collection to understand relationships and utilization within a breeding program. The research objectives were to analyze the genetic structure of the trifecta perennial flax using single-nucleotide polymorphic (SNP) markers for species differentiation and to discover potential Centers of Origin and/or diversity. **Methods**. We tested 70 USDA-GRIN-global wild populations (19 *L. austriacum*, 32 *L. lewisii*, 19 *L. perenne*; N = 850 seedling genotypes) to generate 9804 DArTseqLD SNPs (Group 1). **Results**. After filtering, within *L. austriacum,* 1199 SNPs (261 genotypes; Group 2) remained; in *L. lewisii*, there were 90 unique SNPs (273 genotypes; Group 3); in *L. perenne,* there were 2716 SNPs (309 genotypes; Group 4). Four genetic clusters were detected using STRUCTURE, consistent with principal coordinate analysis and SplitsTrees. Clear distinctions within and among each perennial species’ populations were found, separating into two distinct pure taxon groupings, along with peripheral populations or outliers. Both *L. austriacum* and *L. lewisii* potentially had two putative Centers of Origin, although *L. perenne* had one. **Conclusions**. The occurrence of sympatric *Linum* species in the wild could explain the occurrence of peripheral population groups within each taxon, although other explanations are also possible. This may be consistent with the potential for genetic exchange between the perennial species and the greater genetic variability available. Future research will evaluate additional *Linum* to further delineate the genetic structure and variation within the genus *Linum*.

## 1. Introduction

*Linum*, flax or linseed, is the largest genus in the Linaceae with 300 species [1]. The most widely cultivated species is annual flax, *L. usitatissimum* L. (2*n* = 2*x* = 30) [2]. Cultivation of flax began as early as 30,000 years ago [3]. Early domestication of annual flax focused on fiber traits—plants that displayed long unbranched stems—and oilseed phenotypes, short plants with heavily branched stems for mechanical harvesting [4,5,6]. Linen fabric is made from natural flax stem fibers, which have high tensile strength, density, luster, and biodegradability [7]. The first genome assembly of *L. usitatissimum* ‘Bethune’ employed the Illumina sequencing platform, capturing 85% of the genome consisting of 43 K protein-coding genes [8]. After the assembly of the *L. usitatissimum* genome, developments have been made in genetic marker and quantitative trait loci marker discovery. Investigations into the Canadian annual flax collection led to the discovery of over 1.7 million SNPs from high-density genotypes using sequencing analyses [9,10,11,12]. Recently, the *Linum lewisii* Pursh ‘Maple Grove’ genome was sequenced, with a genome size of 642.9 Mb and an annotated assembly size of 38,808 genes, although it was notably missing the single-copy *TSS1* present in *L. usitatissimum*, possibly due to heterostyly [13,14]. Both *L. austriacum* L. and *L. perenne* L. have heterostyly (distyly), whereas *L. lewisii* has homostyly (similar to *L. usitatissimum*) [1].

Current global flax production is 3.37 million tonnes, with Canada being the largest producer [15], followed by China, India, and the United States. In 2023, 56,656 ha of annual flax were grown in the U.S., with an average oilseed yield of 25–48 bushels/ha [16].

The continued popularity of healthy foods for human consumption has increased the food and byproduct uses of annual flax, since it contains compounds that impact human health, such as dietary fiber, proteins, oils, and lignan [17,18]. Cultivars average an oil content of 38–45%, with 50–62% of that being alpha-linolenic acid (ALA), an omega-3 fatty acid [18], with flax having the highest concentration in its seed oil out of all cultivated crops [17,18]. Additionally, flax also has potential for ornamental uses, either as a cut flower for floral arrangements, ornamental herbaceous annual bedding, or perennial garden plants [19,20,21,22,23,24,25,26,27].

While annual flax is an adaptable heat/drought-tolerant crop, its production disadvantages include a 1x/year harvest for oilseed and/or fiber production, leaving bare soil to erode during much of the year. Thus, interest in perennial flax cropping has come to the forefront in reviving breeding efforts to revolutionize oilseed and fiber production, as well as other novel traits afforded by perenniality [21].

Recent creation of a perennial flax breeding program at the University of Minnesota has focused attention on numerous agronomic and horticultural traits that would transform flax production for wider adaptability as a “green” herbaceous perennial crop [19]. We have invoked crop ideotype breeding strategies for the simultaneous breeding and selection of multiple agronomic, ecosystem services, and horticultural phenotypic traits for oilseed, fiber, pollinators, cut flower, and herbaceous perennials [19,20,24,25,26,27]. Perennial flax species have undergone little breeding and selection for any/all of these traits [21,28,29]. The available perennial flax cultivars on the market, primarily for pollinator and/or ornamental purposes, predominantly include *L. perenne* [21] and *L. lewisii* [30], although *L. austriacum* is also of interest as a perennial flax taxon [19,21]. A recent study of perennial flax species for winter hardiness demonstrated a range in variation for winter survival in USDA Z3 and Z4 [23].

The trifecta of perennial flax species studied herein range in plant height from 20 to 80 cm, forming a crown as the overwintering storage organ with stems arising from cotyledonary nodes, and with small linear to lanceolate-linear leaves [13,14,17,19,30]. All inflorescences are open cymes, primarily with blue flowers. Pollinated flowers produce seed capsules that split along the numerous sutures dividing the locules; seed shattering in the wild commonly occurs in all three species.

*Linum austriacum* (2*n* = 2*x* = 18), Asian flax (section Adenolinum), is native from eastern and central Europe to western and central Asia [31,32], and is considered to be part of the *L. perenne* group in Europe [31], as well as a relative species to *L. usitatissimum* [33]. It has naturalized in western Europe and North America. Similar to *L. usitatissimum, L. austriacum* sports blue flowers and displays a range of plant habits that would make it adaptable to a wide range of crop uses. In dichotomous taxonomic keys, the species can be keyed out based on the existence of heterostyly (differential lengths of anthers and styles among genotypes; distylous for *L. austriacum* with classic pin/thrum flower morphs) [34], as well as “Leaves moderately crowded, the longest at least 10 mm long and Pedicels deflexed or patent” [31,32]. It has a self-incompatibility system linked with distyly [35]. Seeds are produced in multiloculated capsules. There are three subspecies, i.e., *L. austriacum* ssp. *austriacum*, *L. austriacum* ssp. *squamulosum*, and *L. austriacum* ssp. *collinum* [36]. In some native habitats, *L. austriacum* is sympatric with other *Linum*, e.g., in the Crimean Peninsula, Sevastopol, Ukraine, it grows with *L. tenuifolium* L. and *L. corymbulosum* Reichenb. [37], providing a potential opportunity for cross-pollination, provided the taxa are within the same species gene pool [38]. Arylnaphthalene lignans (secondary metabolites) with antiviral and antiproliferative properties have been identified in *L. austriacum* as well as *L. perenne* [39]. Of the 18-chromosome species in section Adenolinum, the C/DAPI bands in *L. austriacum* are smaller than those in *L. leonii* and *L. perenne* [40]. Randomly amplified polymorphic DNA (RAPD) analysis showed *L. austriacum* to be more closely aligned with *L. leonii*, followed by *L. perenne* [40].

*Linum lewisii*, Lewis’ flax or prairie flax, is in the Linum section (2*n* = 2*x* = 18) [41] and is native to western North America, i.e., the state of Alaska, U.S., and Churchill, Manitoba, Canada, southward to Baja California, Mexico, and from the coastal Pacific Ocean eastward to the Mississippi River [13]. The species was widely used by Native American Tribal Nations as a fiber and oilseed food crop [42]. It is also a blue-flowered perennial flax species occurring in a wide range of native habitats [43,44]. One additional blue-flowered flax species is native to North America: annual *L. pratense* [44]. Each of the 10 locules/capsules produces one ovule or seed. Seed dormancy has been reported in limited studies [13], although after-ripening can reduce seed dormancy levels. *Linum lewisii* is unusual as it is homostylous, with a single floral morph with style lengths longer than or as long as the anthers, known as facultative autogamy, necessitating insect pollen vectors (primarily dipterans and hymenopterans) to transfer the pollen [45]. Inbreeding rates could vary among populations due to differing style/anther lengths observed [44]. Many traits in *L. lewisii* make it attractive for domestication as perennial flax, including blue flowers, drought/heat tolerance, winter hardiness, self-compatibility (due to homostyly), large seed size, and a small genome size [13,19,22,46,47]. It has been an important crop for restoration efforts in the western U.S., including the development by the U.S. Department of Agriculture of *L. lewisii* ‘Maple Grove’, which was released in 2004 (from wild populations in Millard County, Utah, U.S.) [13,14].

*Linum perenne* (2*n* = 2*x* = 18), blue flax or perennial flax (section Adenolinum) [48,49], is native to Eurasia and is sympatric with other *Linum* species, including *L. austriacum* [3,17,28]. Similar to *L. austriacum*, perennial flax has naturalized in western Europe as well as North America [48,49]. *Linum perenne* is self-incompatible and distylous [31,36], flowering indeterminately. Seed shattering has been observed [49]. The latter trait might indicate the potential for multiple harvests during a growing season. It is an evergreen broadleaf herbaceous perennial plant with primarily blue flowers, although white and/or bicolored flowers have been reported. *Linum perenne* is grown for revegetation and horticultural purposes in the states of Iowa, North Dakota, and Oregon, U.S., including the commercial variety, *L. perenne* ‘Appar’ [13,46]. Since both *L. perenne* and *L. austriacum* have naturalized in the U.S. and western Europe and they share the same chromosome numbers, as well as being in the same section of the genus *Linum*, peripheral populationization remains a distinct possibility. However, these would not naturally cross-pollinate with *L. lewisii*.

Screening the flax taxa contained within the world germplasm banks would provide useful phenotypic and molecular data for incorporation into the perennial flax breeding program. In this first flax germplasm study within this context, utilizing genomic tools for the trifecta perennial flax species would allow for greater progress and directionality in breeding efforts. Screening perennial germplasm in available collections would allow for broadening the genetic base and improving the efficiency of marker-assisted selection. The benefits of genetic tools rely on further research and discovery in wild populations, as well as germplasm repository collections. Research objectives are to analyze the genetic structure of the three perennial *Linum* species for single-nucleotide polymorphic (SNP) markers for species differentiation and to discover potential Centers of Origin and/or diversity within each taxon.

## 2. Materials and Methods

### 2.1. Germplasm

Perennial *Linum* species used in this analysis were from the USDA’s Germplasm Resource Information Network Global collection (GRIN-Global; https://www.grin-global.org/) and the Plant Gene Resources of Canada (https://pgrc-rpc.agr.gc.ca/gringlobal/landing (accessed on 9 August 2025)), with a focus on three perennial flax species, *L. lewisii, L. perenne*, and *L. austriacum*, for this study. Seeds of 70 GRIN-Global accessions (19 *L. austriacum*, 32 *L. lewisii*, and 19 *L. perenne*) were sown, germinated in the greenhouse (n = 1630 seedling genotypes in total), and transplanted into the field (n = 850 seedling genotypes; Supplemental Table S1) in a completely randomized design (n ≤ 20 reps, seedling genotypes/accession). This resulted in a total of 850 individual seedling genotypes from the 70 accessions sampled for sequencing, 843 of which also produced SNPs after filtering (see below). Samples were not pooled within accessions due to segregation. The following replicated samples were grown and run for each of the species: *L. austriacum* (2 reps. PI 650297, plant no. 11; 2 reps. CN 107288, plant nos. 1,2,3,5,6), *L. lewisii* (2 reps. Ames 32565, plant nos. 1, 3–20), and *L. perenne* (2 reps. ‘Blue Flax’ plant no. 1). Each biological replicate consisted of two samples (rep1, rep 2), each of which originated from a single leaf, which were extracted and sequenced separately.

### 2.2. Greenhouse/Field Growing Conditions

All flax accessions were sown into 288 plug trays containing Berger BM2 soilless germination mix (Saint-Modeste, QC G0L 3W0, Canada), with a covering of fine Palmetto vermiculite (Fine-Medium, A2; Vermiculite Co. Inc., Woodruff, SC, USA) during week 13 (2019). Seeds were germinated for 1–8 weeks in greenhouse conditions (21/21 °C, day/night, 16 h; minimum lighting set point at 150 μmol m^−2^ s^−1^) with misting (10 min intervals, 0600–2200 h, 7 s duration) at the St. Paul campus Plant Growth Facilities greenhouses, University of Minnesota (44.98766632135765, −93.18086973245858). Once >75% of the seeds had germinated, the seedling plug trays were moved to capillary mats in a greenhouse set to 15.6 °C day/night with a 14 h photoperiod (0600–2000 HR; long days) supplied by 400 w high-pressure sodium high-intensity discharge (HPS-HID) lamps, at a minimum of 150 μmol m^−2^ s^−1^ at plant level [22,23,24,50]. Fertigation water was applied twice daily, between 0700–0800 h and 1600–1700 h, using a constant liquid feed (CLF) of 125 ppm nitrogen (N) supplied from a water-soluble 20N–4.4P–16.6K fertilizer (Scotts, Marysville, OH, USA). Monthly rotational fungicide drenches were administered. Plants were then transplanted into the field at a spacing of 61.0 cm on-center (O.C.) within and between rows using a completely randomized design [50]. Post-planting irrigation occurred weekly (2.54 cm water) unless rainfall occurred. The field was fertilized with urea (56 kg/ha actual N) and chemical weed control was provided by Fortress pre-emergent herbicide (a.i. Isoxaben + Dithiopyr; EPA registration no. 59807-19; 168 kg/ha rate; OHP, Inc., Morrisville, NC, USA). Additional weed control consisted of mechanical tillage between rows and hand weeding within rows, as needed.

### 2.3. Plant Material Sampling and DArTseqLD Genotyping

Leaf sampling for SNP analyses occurred once mature true leaves developed. In 2019, tissue samples were collected, placed into Cillia^TM^, Melitta^®^ unbleached paper tea filters (Brews Better tasting Tea Co., Clearwater, FL, USA), and dried at 95 °C, with full desiccation occurring after 3–5 d [50]. Dried samples (25 mg/ground) were loaded into 96-well plates (MicroTube Rack System, Lot: VF3013611). To each well, a 3 mm steel bead (Retsch, 22.455.0011) was added and covered with cap strips (In-line Micro Tube, Lot: VG3013742). All tissues were ground using a Geno/Grinder^®^ Tissue Homogenizer, SPEX SamplePrep (Avantor, VWR, Barrington, IL, USA) for 10 min at 1500 rpm [50] and sent to DArT (Diversity Arrays Technology Pty Ltd., Yarralumla, Australia; https://www.diversityarrays.com/) for low-density (LD) genotype by sequencing (GBS) to generate SNPs. All DNA extraction, purification, and quantification was conducted at DArT.

DArTseq is a high-throughput, proprietary genotyping-by-sequencing method [50] using restriction enzymes to reduce genomic complexity. Fragment populations are hybridized to microarrays to assay genome-wide markers [50] and produce large numbers of SNPs via restriction fragment sequencing. It is a cost-effective means of identifying genome-wide molecular markers in species without sequenced genomes or classic genetic maps. We employed it to generate “low-density” (LD) SNP coverage and apply an optimal “call rate”, or the proportion of genotypes in which the genotype call is not missing. This was achieved by examining histograms of loci and individuals and selecting a call rate that retained a large number of individuals while excluding low-quality loci.

### 2.4. Genetic Diversity and Population Structure Statistical Analyses

Analyses for the perennial *Linum* species, together (Group 1) and individually (Groups 2–4), were performed, and foundations of interrelationships were based on SNP data analyzed for genetic diversity (principal component of analysis, PCoA) and genetic STRUCTURE [51]. An initial pilot plate of genetically diverse *Linum* taxa was used to elucidate the extraction and filtering of SNPs by DArTseq for this analysis. Subsequent SNP analyses were on a per-species basis, and separate SNP panels were created for each of the three perennial flax species. Four groups were established for the purposes of statistical analysis: *L. austriacum*, *L. lewisii*, *L. perenne* (all three perennial flax species; Group 1); *L. austriacum* (Group 2); *L. lewisii* (Group 3); and *L. perenne* (Group 4). These groupings were used for analyses of species interrelationships using SNP data analyzed for genetic diversity (PCoA), using the R Studio dartR Version 4.2.3 package [52], genetic STRUCTURE (Version 1.2.5033) [51], STRUCTURE Harvester, and GenAlEx 6.5, using Microsoft Excel 365 (Microsoft, Redmond, WA). Resulting PCoAs were based on SNPs filtered by call rate by locus, and genotypes beyond the threshold of 0.90 missing data were removed. Genotype similarity could then be visualized with PCoAs [52], using the ADE4 and ADEGENET packages.

While multiple components can be evaluated in the PCoA, the standard protocol is to take into consideration the first two principal components, PCoA 1 and PCoA 2. Positions along the locus axis were determined by genotype (0 for the homozygous reference SNP, 2 for the homozygous alternate SNP, and 1 for the heterozygous state) in a 2D space defined by the loci [53]. In the PCoA plot, the PCoA-1 axis represents the most variation and the PCoA-2 axis represents the most residual variation. Clusters of genotypes will be formed based on potential relationships between subpopulations in each group, later to be compared with other forms of genetic population analyses.

Genetic STRUCTURE group evaluation was performed using the Bayesian clustering method implemented with STRUCTURE v.2.3.4 [54,55,56]. Genotypes were assigned to the K number of clusters using the Admixture Ancestry Model with independent allele frequencies [56]. For each group, K values ranging from 1–15, 100,000 for the length of the burn-in period, and a 100,000-step run time were used. The STRUCTURE analysis had three replications, with values of subpopulations (K) 1–15. The outputs from STRUCTURE, the estimated likelihood value of data [lnP(D)], and the ad hoc statistic Delta K (ΔK) were visualized using the maximized log value of the plot of peak(s) to illustrate the best K value using STRUCTURE Harvester [54,55,56] and plotted as x- and y-axes in Microsoft Excel, which illustrated the best ∆K as maximized log values (peaks).

The division of variation of species was calculated using the Analysis of Molecular Variance (AMOVA) in GenAlEx in Microsoft Excel 365 (Microsoft, Redmond, WA, USA), based on the genetic perspective of clusters. GenAlEx was used to estimate genetic variation and the percentage of polymorphism (%P) among regions, among and within populations. For the Φ(Phi) coefficient, the association between two binary variables was used to test the Ho hypothesis: there is no genetic difference among or within species used (ΦRT), within and among populations or accessions (ΦPR), or among individuals (ΦPT) within populations or accessions [53].

The SNP markers from the various *Linum* species were used to calculate the genetic diversity, based on the Jaccard similarity index [57], and transformed into a distance matrix to construct dendrograms using the unweighted pair group method (UPGMA) with 1000 bootstrap replicates. SplitsTree (CE) used neighbor joining to compute unrooted phylogenetic networks using the SNP molecular sequence data [58].

An input file constituting a matrix of Euclidean distances between populations was created in dartR using the gl2phylip function. A splits network was then constructed using the default neighbor-net method [59].

## 3. Results

### 3.1. SNP Markers and Genotyping

The resulting DArTseqLD DNA sequencing of Group 1 produced 9804 polymorphic binary SNP markers from 850 individual genotypes from the three perennial flax species, *L. austriacum, L. lewisii*, and *L. perenne*. After filtering, the dataset was refined to 1128 SNPs and 843 individuals with a minimum call rate of 90%. The filtered *L. lewisii* population resulted in 90 SNPs and 273 genotypes (Group 3). *Linum perenne* was filtered to 2716 SNPs and 309 genotypes (Group 4), whereas *L. austriacum* resulted in 1199 SNPs and 261 individual genotypes (Group 2).

#### Population Genetic Structure (Groups 1–4)

- Group 1

**Figure 1:**
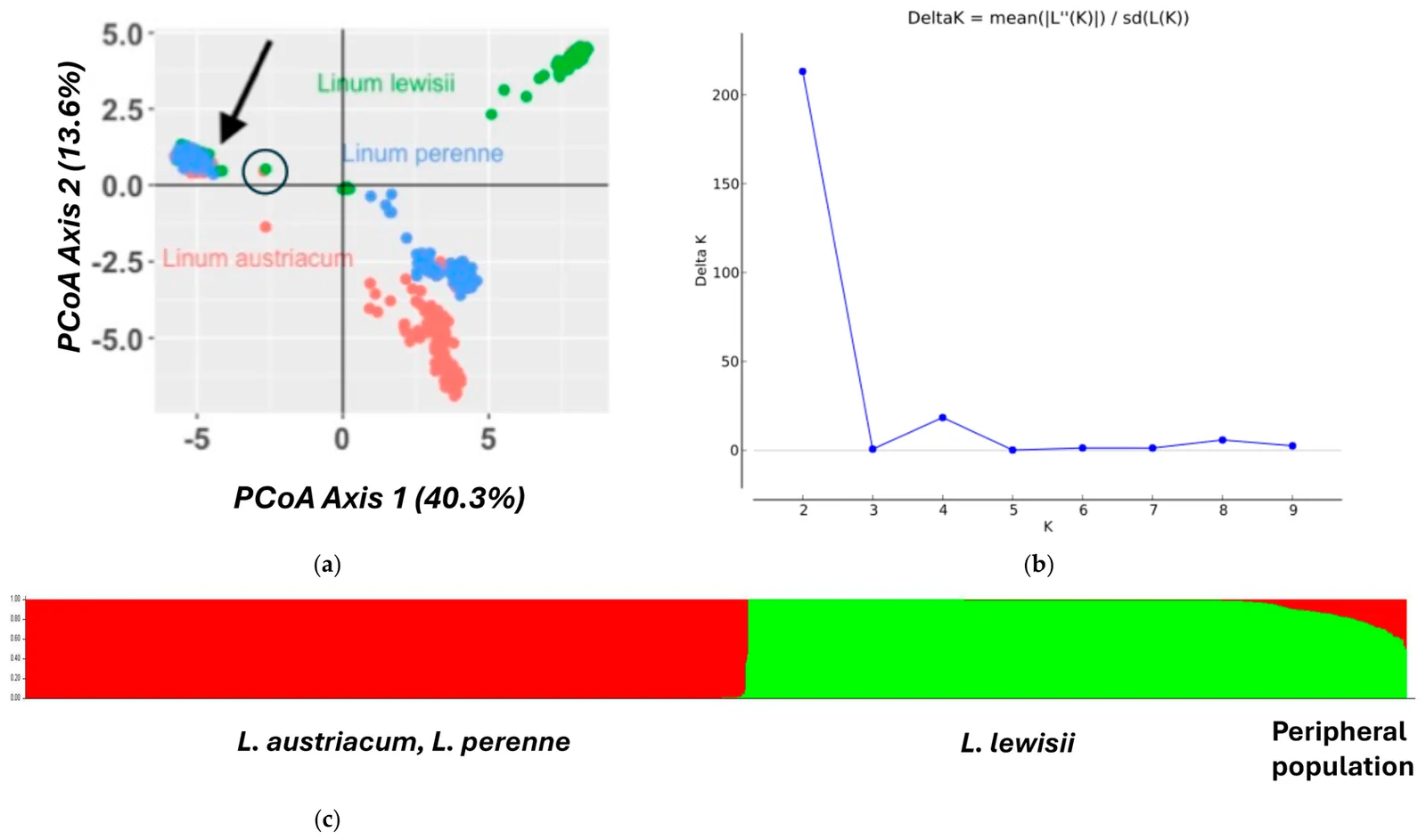
Genetic structure and diversity of the trifecta perennial flax species, *Linum austriacum, L. lewisii,* and *L. perenne* (Group 1) for accessions (wild or cultivated populations, cultivars) and their genotypes (n = 1–20/accession) in sampled single-nucleotide polymorphisms (SNPs). (**a**) Principal coordinate analysis (PCoA) plot for the first two coordinates (PCoA 1 and PCoA 2). Arrow denotes the peripheral population; the circle denotes the outlier peripheral population. (**b**) Estimation of K based on a filtered dataset using ΔK, following Evanno et al. [54]. Generated graph was produced via Structure Harvester [55]. Highest ΔK occurs at K = 2, 4; (**c**) single-line Q-plot denoting the separation of the filtered dataset for K = 2, as determined by STRUCTURE analysis.

PCoA analysis of the three perennial species (Group 1) showed four clusters, with a distinct delineation within each species into three of the clusters (Figure 1a). The percent contribution to the PCoA axis peaked at zero (40.3%), with all eigenvalues being positive (Figure 1b). The percentages are on a relative scale of 0 to 100%, so the majority of variation in the dataset is explained by the first two principal components or dimensions. Genotypes that were identical to each other or closer to one another on the PCoA are genetically more similar than genotypes more distant from one another. Geometric distances in the PCoA between individuals reflect genetic distances among individuals [60]. Each taxon formed distinct clusters in the PCoA, along with a “peripheral population” cluster. Genotypes contained within each taxon are identified and arise from numerous flax seed sources for varying accessions (Table 1). Genotypes in the “peripheral population” clustering did not have any anomalies found in their call rates or percent missing data prior to imputation; an explanation for the designation of this grouping is provided below for each taxon. The three clusters denote each set of *L. austriacum, L. lewisii,* and *L. perenne* taxa, whereas the additional cluster contained all three species’ genotypes intermixed together into one interrelated (shared SNPs) germplasm pool. Principal component 1 (PCoA1) or dimension 1 accounted for 40.3% of the variation, while principal component 2 (PCoA2) or dimension 2 encapsulated 13.6% of the genetic variation (Figure 1a). The fourth cluster in the Group 1 PCoA is composed of all three perennial flax species (denoted by the arrow, Figure 1a), containing a wide array of genotypes (n = 411) in a germplasm pool from across the n = 843 (48.8%) total genotypes. These 411 genotypes constitute the three-species SNP arrays (n = 264 *L. austriacum*; n = 276 L. lewisii; n = 309 *L. perenne*). The n = 411 genotypes in the germplasm pool are categorized as the “peripheral population” in the SNP analyses henceforth (Table 1), with the exception of n = 3 genotypes, which separated from their respective species but were distinctly separated from the hybrid cluster grouping: n = 2 *L. austriacum* (19F-224, PI 650296-1; 19F-245, PI 650297-6) and n = 1 *L. lewisii* (19F-1577, Ames 32565-3) (Figure 1a; Supplemental Tables S1 and S2); these are categorized as the “outlier peripheral population”.

In the germplasm pool’s “peripheral population” cluster, there are more *L. perenne* (226/309 = 73.1% of species total) than *L. lewisii* (95/276 = 34.4%) and *L. austriacum* (90/264 = 34.1%) genotypes (Supplemental Table S1). Genotypes were distributed across various *Linum* germplasm sources in this study (USDA GRIN, PI, or AMES; Canadian germplasm, CN; Alaska wildflowers; commercial sources). Most genotypes within each accession fell within the “peripheral population” cluster rather than also being in the pure species clusters. For example, in *L. austriacum* (excluding the two “outlier peripheral population” genotypes), 100% of sampled genotypes (up to n = 20 genotypes/accession) within accessions PI 650293 (n = 20), PI 650295 (n = 17), CN 107255 (n = 20), and CN 107288 (n = 13) were within the “peripheral population” cluster (Figure 1a; Supplemental Tables S1 and S2). However, a genetic segregant was found in PI 650294: only genotype 5 was in this cluster; the remaining genotypes (1–4, 6–11, and 13–20) were pure species. In *L. lewisii* (excluding one “peripheral population” genotype, PI 522306 genotype 20), 100% of sampled genotypes within accessions PI 522306 (n = 20), Ames 32565 (n = 9, 2 reps ea.), PI 522305 (n = 12), and CN 107266 (n = 12) were within the “peripheral population” cluster (Figure 1a; Supplemental Tables S1 and S2). Genetic segregation occurred in PI 650320, wherein genotype 6 was in the pure species clusters, whereas all others (n = 18) were in the “peripheral population” cluster (Figure 1a; Supplemental Tables S1 and S2). In *L. perenne*, 100% of sampled genotypes within accessions PI 650327 (n = 19), PI 650328 (n = 19), PI 650329 (n = 20), Ames 2122 (n = 16), Ames 3137 (n = 20), PI 650326 (n = 19), ‘Blue Flax’ (n = 20, including all reps), PI 445972 (n = 17), AKWildflower (n = 14), PI 445972 (n = 19), CN 10726 (n = 2), CN 19024 (n = 14), CN 107256 (n = 18), and CN 107270 (n = 20) were within the “peripheral population” cluster (Figure 1a; Supplemental Tables S1 and S2). No genetic segregants were found within any *L. perenne* accessions tested.

For the replicated SNP sampling in *L. austriacum* (2 reps. PI 650297, plant no. 11; 2 reps. CN 107288, plant nos. 1,2,3,5,6), *L. lewisii* (2 reps. Ames 32565, plant nos. 1, 3–20), and *L. perenne* (2 reps. ‘Blue Flax’ plant no. 1), there were no differences among biological replications for SNPs and locations within the PCoA. All of these were grouped in the “peripheral population” cluster (Supplemental Tables S1 and S2; Figure 1a) with identical SNPs. Genetic segregants were found within *L. austriacum* accessions as being either “peripheral population” [PI 650294 (genotype 5 only), PI 650297 (genotypes 1–4, 6–11, and 13–20)] or in the pure species cluster [PI 650294 (all genotypes except for 5)].

**Figure 2:**
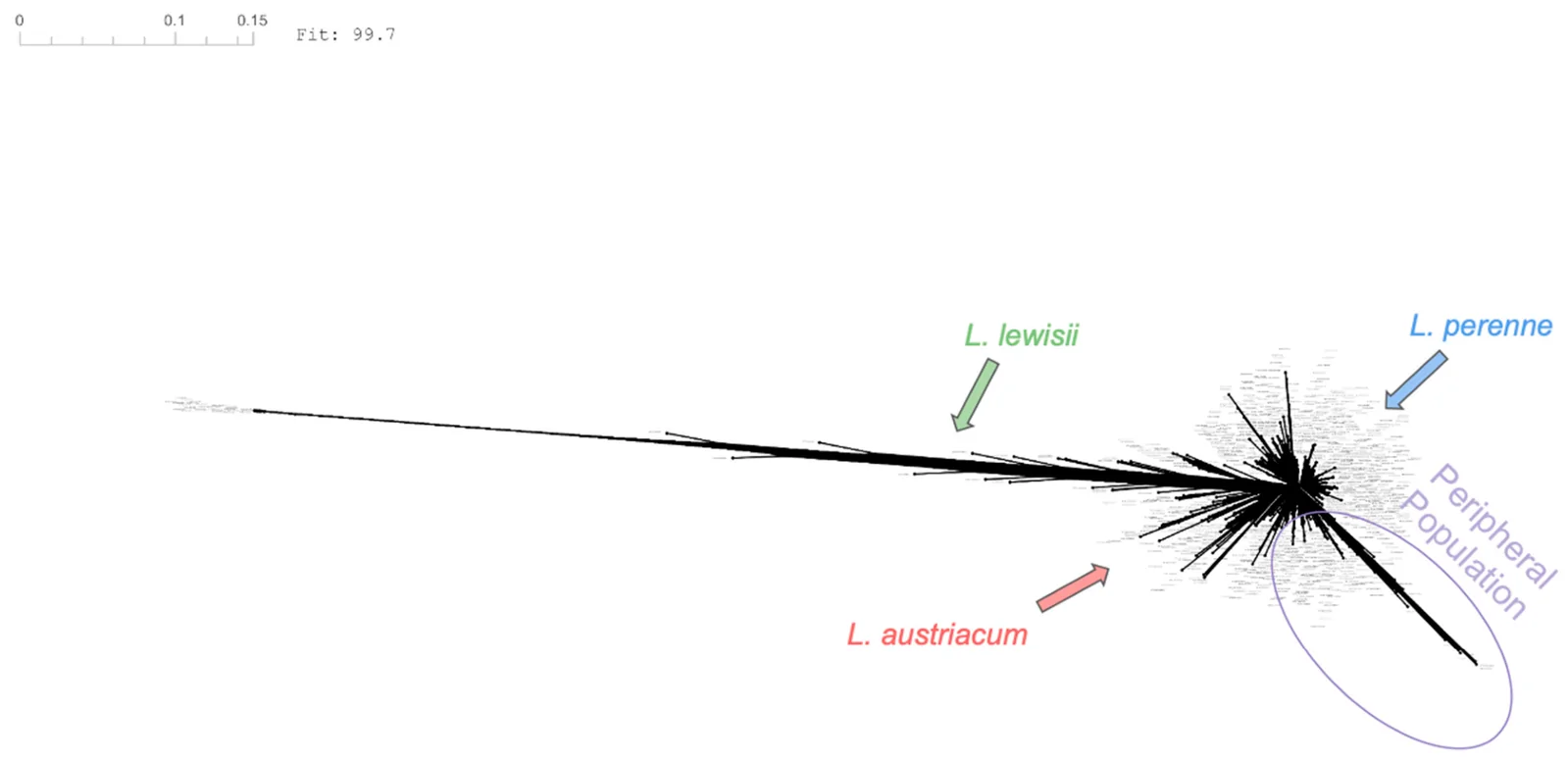
Unrooted phylogenetic tree (SplitsTrees) from single-nucleotide polymorphism (SNP) data for taxa in the trifecta perennial flax species, *Linum austriacum, L. lewisii*, and *L. perenne,* and the peripheral population (Group 1).

Branching in the phylogenetic splits tree network (Figure 2) for the complete trifecta dataset (Group 1) complements the PCoA clustering patterns for the complete dataset (Figure 1a). Similarly, branches in the splits tree group by taxon and peripheral populationization, as the branch lengths in the splits tree network correspond to the weight of the split [58]. Since the splits tree network branch lengths are calculated using genetic distances, a long branch length corresponds to a large genetic distance, and short branches to smaller genetic distances. For example, the *L. lewisii* branch is the longest, representing a large genetic distance among populations of the other two taxa and the peripheral populations group. This is not surprising, since *L. lewisii* is a N. American native perennial flax species. In contrast, *Linum austriacum* and *L. perenne* are Eurasian in origin and are closely related but distantly related to *L. lewisii*. The “peripheral populations?” group spreads above all three of the taxa, along with a shorter branch, due to the representation of all three taxa within this group (Supplemental Tables S1 and S2; Figure 1a and Figure 2).

- Group 2

**Figure 3:**
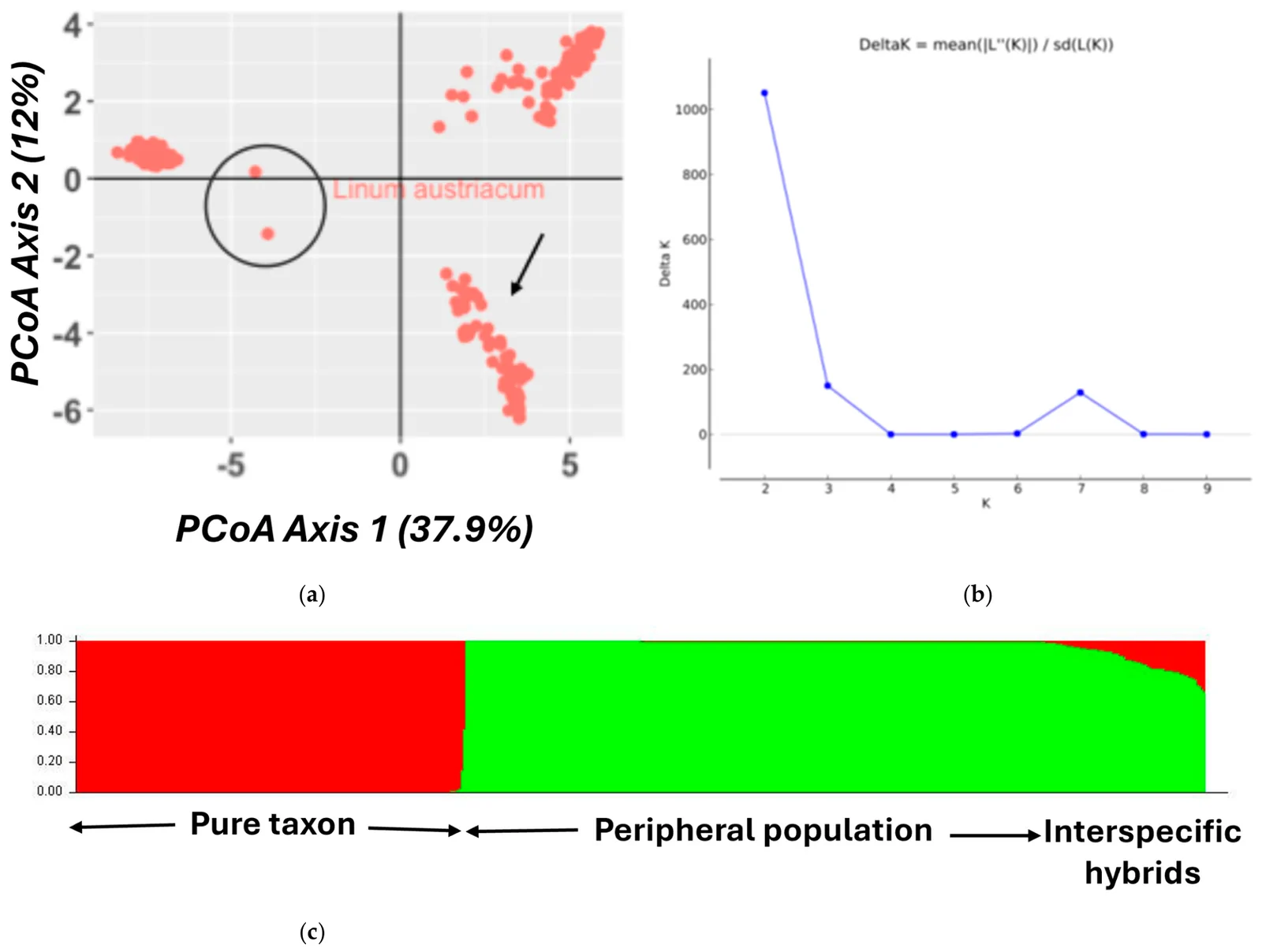
Genetic structure and diversity of perennial flax, *Linum austriacum* (Group 2), for accessions (wild or cultivated populations, cultivars) and their genotypes (n = 1–20/accession) in sampled single-nucleotide polymorphisms (SNPs). (**a**) Principal coordinate analysis (PCoA) plot for the first two coordinates (PCoA 1 and PCoA 2); the arrow denotes the peripheral population; the circle denotes the outlier peripheral population. (**b**) Estimation of K based on the filtered dataset using ΔK, following Evanno et al. [54]. Generated graph was produced via Structure Harvester [55]. Highest ΔK occurs at K = 2, 7; (**c**) single-line Q-plot denoting the separation of the filtered dataset for K = 2, as determined by STRUCTURE analysis.

In the Group 2 analysis of *L. austriacum* accessions and genotypes, the percent contribution to the PCoA axis peaked at zero (37.9%), with all eigenvalues being positive. For the 1199 *L. austriacum*-identified SNPs, the first PCoA dimension (Axis 1) described 37.9% of the variation among genotypes in Group 2, and the second dimension (Axis 2) represented 12% of the variation (Figure 3a). As noted earlier, geometric distances in the PCoA between genotypes reflect genetic distances among genotypes [60]. Similar to findings in Group 1 analyses, the Group 2 pure species taxon (*L. austriacum*) formed two distinct clusters in the PCoA, along with a third “peripheral population” cluster (Figure 3a).

Genotypes contained within the taxon are identified and arise from numerous flax seed sources for varying accessions, although most of the pure taxon accessions arise from wild populations in Ukraine, the Russian Federation, Armenia, Bulgaria, and Hungary (Supplemental Table S1). The pure species taxon group with both positive PCoA Axis 1 and 2 values include wild species collections from northern regions in eastern Europe, i.e., the Russian Federation (Yankuli, Stavropol hills, central Caucasus, PI 650294; West, 250 km of Elista, Kalmyk ARR, PI 502410; Division 5, Sheep & Goat Farm, Ravine 4, 52 km SE of Stavropol, PI 440472; Beshpagin Sands, 44 km southeast of Stavropol, PI 440473), Ukraine (Opuk National Preserve, 20 km southeast of Marfivka, Ames 29749; Steppe, Askania-Nova Preserve, PI 502405), and Armenia (Near Jil, Gegharkunik Marz, PI 650301). In contrast, the pure species taxon group with positive PCoA Axis 1 but negative PCoA Axis 2 values were wild species collections from more southern eastern European areas, i.e., Hungary (near Tatatszentgyorgy and Orgovany, the Great Hungarian Plain between Duna (Danube) and Tisza Rivers, Kiskunsag, Bács-Kiskun, PI 650300), Bulgaria (above Albena, Dobrich, PI 650296; Along roadside near Kaliakra, close to the Black Sea, Dobrich, PI 650299; along roadside near Kaliakra, close to the Black Sea, Dobrich, PI 650298), and an unknown country (no passport data, CN 107288-6). The only exception to this was some genotypes within the Russian Federation population (Division 5, Sheep & Goat Farm, Ravine 4, 52 km SE of Stavropol, PI 440472), which also had other genotypes that were within the other pure species taxon group.

Genotypes in the “peripheral population” clustering did not have any anomalies found in their call rates or percent missing data prior to imputation. However, these accessions and genotypes therein arose from wild populations collected at the Polish Arboretum (PI 650295-2, 19F-207), although their wild origin is Pogorze Przemyskie, Poland; SW of Galgenberges, on a field trail in Gatersleben-Cochstedt, Germany (PI 650297-1, 19F-241); two unknown accessions of “Wild material, no passport data” from the Canadian germplasm repository (CN 107255-1, 19F-1018; CN 107288 Reps. 1 and 2, 19F-1661, 19F-1769, respectively); one genotype (5%) within an accession from Yankuli, Stavropol hills, central Caucasus, Russian Federation (PI 650294-1, 19F-160), as well as all genotypes within the population from Szarsomlyo hill above Nagyharsany, in limestone grassland, Sedo-Festucetum dalmatica, Villany-Siklos range, Hungary (PI 650293-1, 19F-36; Supplemental Table S1). Since most of these accessions were either collected in arboreta or are from unknown collections, the designation “peripheral population” was applied. Two genotypes did not cluster with any other individuals, categorized as “putative interspecific hybrids and/or genotypes showing possible admixture”: 19F-224, PI 650296-1, 5% of the population evaluated from Albena, Dobrich, Bulgaria and 19F-245, PI 650297-6 from Gatersleben-Cochstedt, Germany, which are tentatively categorized as “putative interspecific hybrids and/or genotypes showing possible admixture” (Figure 1a and Figure 3a; Supplemental Tables S1 and S2). These were distinctly different from other groupings within the taxon. The remainder of the German population was categorized as “peripheral population”, as noted above.

**Figure 4:**
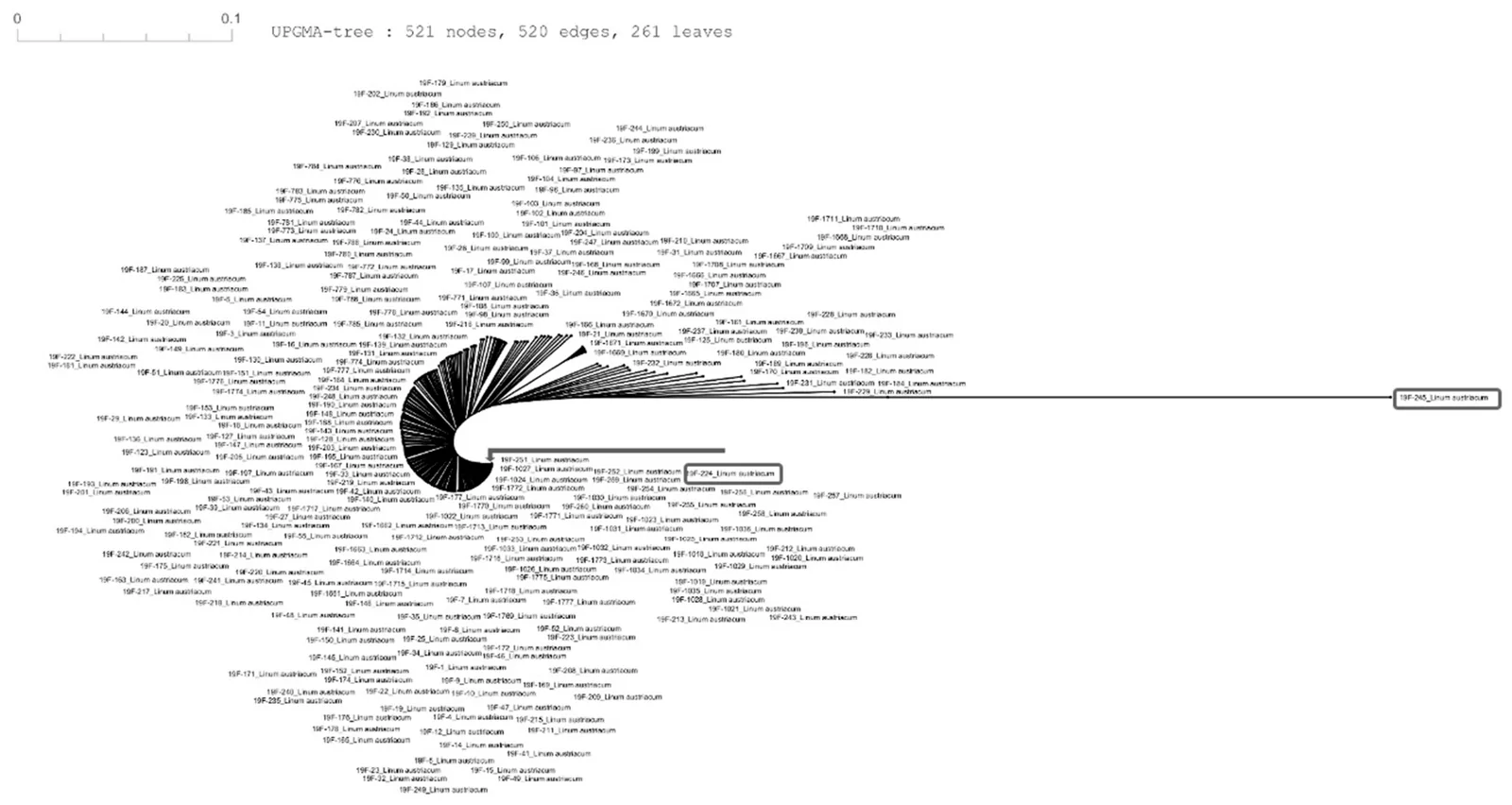
Unrooted phylogenetic tree (SplitsTrees) from single-nucleotide polymorphism (SNP) data for taxa in the perennial flax species, *Linum austriacum* (Group 2). Boxed genotypes denote the outlier peripheral population group.

Branching in the phylogenetic splits tree network (Figure 4) for the complete *L. austriacum* taxon dataset (Group 2) complements the corresponding PCoA clustering patterns for the dataset (Figure 3a). The distribution of the *L. austriacum* genotypes in the phylogenetic splits tree network forms an unusual scorpioid curve shape (Figure 4). The longest branch of the scorpioid curve terminates with one of the “putative interspecific hybrids and/or genotypes showing possible admixture”, 19F-245 (PI 650297-6; Supplemental Tables S1 and S2; Figure 4—highlighted), whereas the other genotype within the “putative interspecific hybrids and/or genotypes showing possible admixture” group, namely 19F-224 (PI 650296-1; Supplemental Tables S1 and S2), is located at the opposite end (beginning) of the scorpioid curve in the phylogenetic splits tree network (Figure 4—highlighted). Genotypes within the “peripheral populations” PCoA cluster were intermixed in the scorpioid curve of the phylogenetic splits tree network (Figure 4).

- Group 3

**Figure 5:**
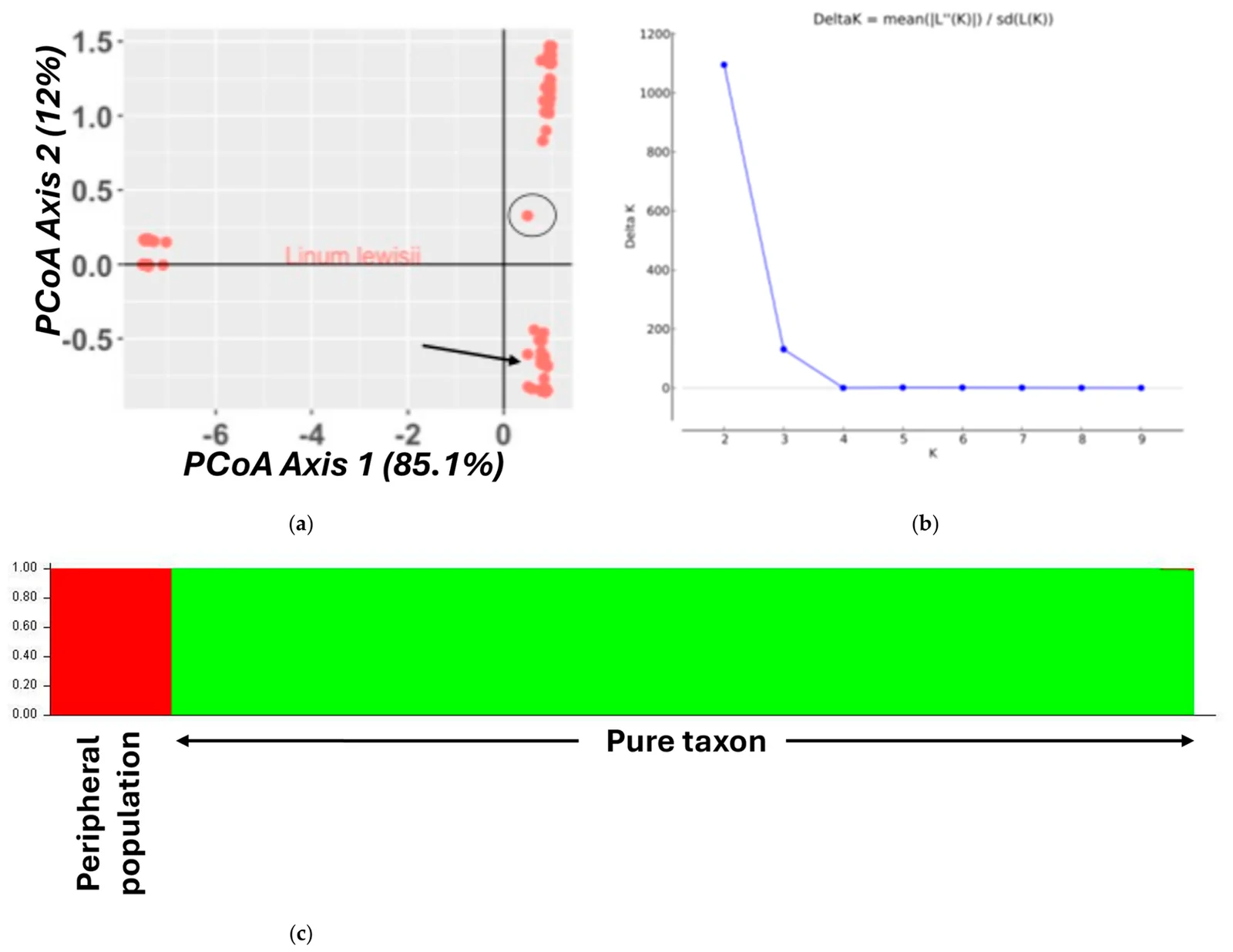
Genetic structure and diversity of perennial flax, *Linum lewisii* (Group 3), for accessions (wild or cultivated populations, cultivars) and their genotypes (n = 1–20/accession) in sampled single-nucleotide polymorphisms (SNPs). (**a**) Principal coordinate analysis (PCoA) plot for the first two coordinates (PCoA 1 and PCoA 2); the arrow denotes the peripheral population of the pure taxon, and the circle denotes the outlier peripheral population. (**b**) Estimation of K based on the filtered dataset using ΔK, following Evanno et al. [54]. Generated graph was produced via Structure Harvester [55]. Peak ΔK occurs at K = 2; (**c**) single-line Q-plot denoting the separation of the filtered dataset for K = 2, as determined by STRUCTURE analysis.

Group 3 analysis of *L. lewisii* taxon accessions and genotypes showed that the percent contribution to the PCoA axis peaked at zero (85.1%), with all eigenvalues being positive. For the 273 genotypes and 90 identified *L. lewisii* SNPs, Group 3 PCoA analysis showed three clusters, with the majority being in two clusters (positive values) in Axis or Dimension 1 (Figure 5a), identified as the taxon. The third Group 3 cluster (left quadrant) contains only 12 individuals, similar to the clustering seen in the PCoA containing all three perennial species (Figure 1a). The first (Dimension 1) coordinate describes 85.1% of the variation seen among individuals, and coordinate two (Dimension 2) describes 12% of the genetic variation.

Wild populations of *L. lewisii* classified as the pure taxon (Figure 5a, Supplemental Table S1), with positive PCoA Axis 1 and PCoA Axis 2 values, were collected in the USA states of Arizona (three populations: Ames 31360, 19F-73; Ames 33353, 19F-323; Ames 31366, 19F-394), Oregon (six populations, Ames 31361, 19F-118; Ames 29912, 19F-296; Ames 31361, 19F-407), Wyoming (two populations, Ames 32568, 19F-309), Nevada (four populations, Ames 33352, 19F-313; Ames 31369, 19F-401), or from states of unknown origin (Ames 33354, 19F-553; PI 452487, 19F-573; Ames 31368, 19F-722; Ames 31364, 19F-904; Ames 31371, 19F-1563; Ames 31372, 19F-1572; Ames 32566, 19F-1596; CN 107300, 19F-1625), and one commercial cultivar ‘Maple Grove’ (Ames 27614, 19F-56). The pure species taxon group with both positive PCoA Axis 1 and 2 values includes wild species collections from the states of Wyoming, USA (Ames 32565; Ames 32565; Ames 31364, genotype no. 17), Utah (PI 650320), and unknown origin in N. America (CN 107266; PI 522306; PI 522305; Supplemental Table S1; Figure 5a). While there were two groups of pure taxa for *L. lewisii*, the geographic locations overlapped for Wyoming and Utah but not the other locations.

In contrast, the group with positive PCoA Axis 1 but negative PCoA Axis 2 values were demoted as peripheral population of the pure taxon (Figure 5a, noted with an arrow) and consisted of wild species collections from the states of Wyoming, USA (Ames 31372; Ames 32568), Utah (Ames 32566; Ames 27614; Ames 27614—‘Maple Grove’), Nevada (Ames 33352; Ames 31369; Ames 33354; Ames 31368), Arizona (Ames 33353; Ames 31366; Ames 31360), Colorado (Ames 31365; Ames 31364), Montana (PI 452487), or are of unknown origin in N. America (Ames 31371; CN 107300; Ames 31370). Interestingly, one genotype in Ames 32565 (genotype no. 1) from Wyoming, USA, and one genotype in PI 522305 (genotype no. 1) were in this group, whereas the remainder of each respective population were in the other pure taxon grouping noted above. In addition, also included in the peripheral population of the pure taxon were primarily four wild populations of uncertain or unknown N. American origin (PI 522306, 19F-261; Ames 32565-1 both reps. 1 and 2 populations, 19F-470; PI 522305-1, 19F-561; CN 107266-1, 19F-1061), while only one accession should have been the pure taxon collected in Utah, U.S.A. (PI 650320, 19F-279) (Supplemental Table S1). The one “outlier peripheral population” genotype also arose in one of the unknown origin populations (PI 522306 genotype 20, 19F-278) but differed in SNP configuration significantly enough to separate it from other genotypes in that PI 522306 population, as well as all other “peripheral population” genotypes identified in other populations.

**Figure 6:**
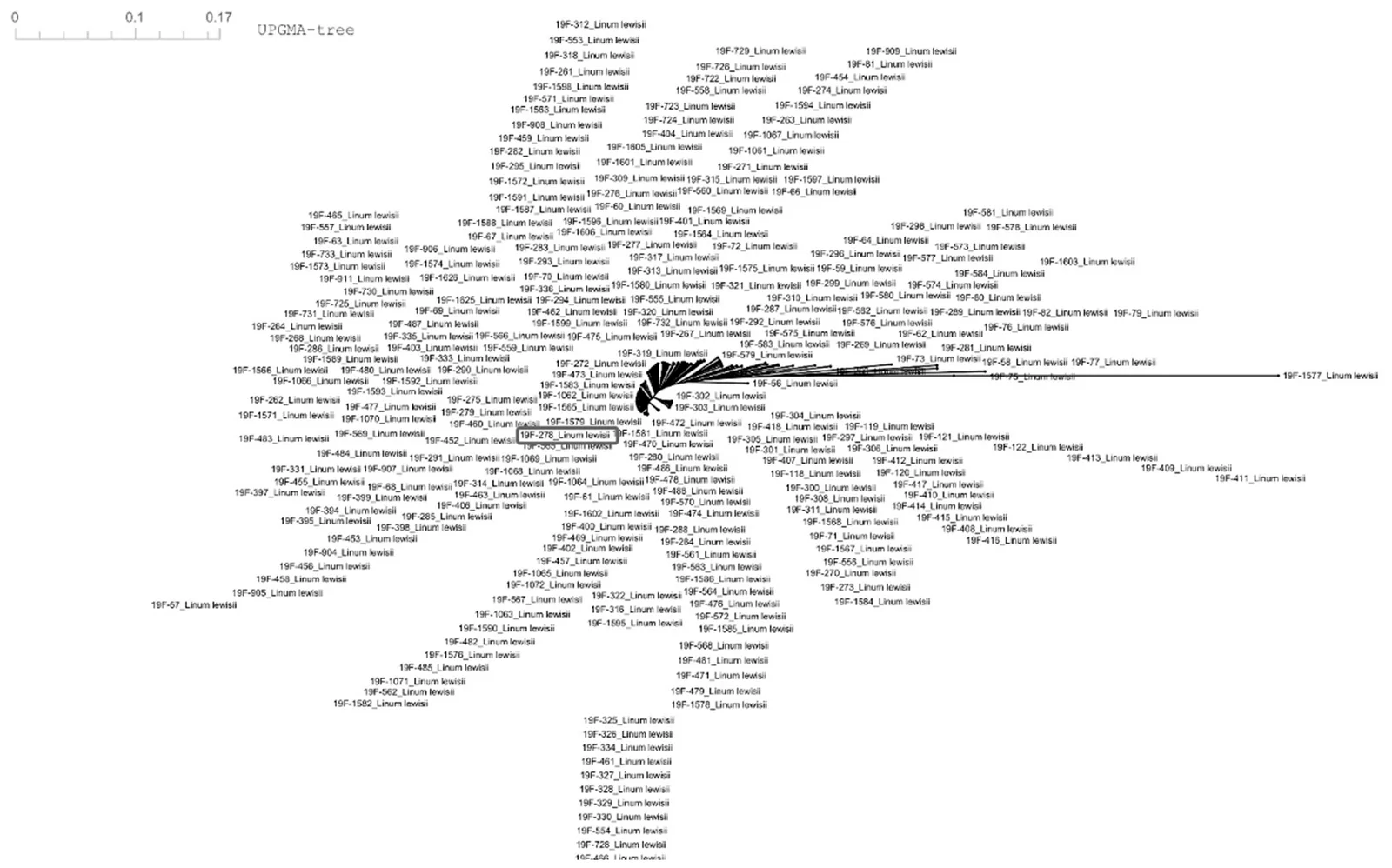
Unrooted phylogenetic tree (SplitsTrees) from single-nucleotide polymorphism (SNP) data for taxa in the perennial flax species, *Linum lewisii* (Group 3), with the peripheral population genotype boxed.

Branching in the phylogenetic splits tree network (Figure 6) for the complete taxon dataset (Group 3) complements the corresponding PCoA clustering patterns for the dataset (Figure 5a). The distribution of the *L. lewisii* genotypes in the phylogenetic splits tree network also forms an unusual scorpioid curve shape (Figure 6). The sole *L. lewisii* genotype within the “outlier peripheral populations” group, namely 19F-278 (PI 522306-20; Table 1), is located at the beginning of the scorpioid curve in the phylogenetic splits tree network (Figure 6–highlighted in red). The genotype at the end of the long tail is 19F-1577 (Ames 32565-3 rep2; Table 1). Genotypes within the “peripheral populations” PCoA cluster (Figure 5a) were primarily intermixed in the center of the scorpioid curve in the phylogenetic splits tree network (Figure 6).

- Group 4

**Figure 7:**
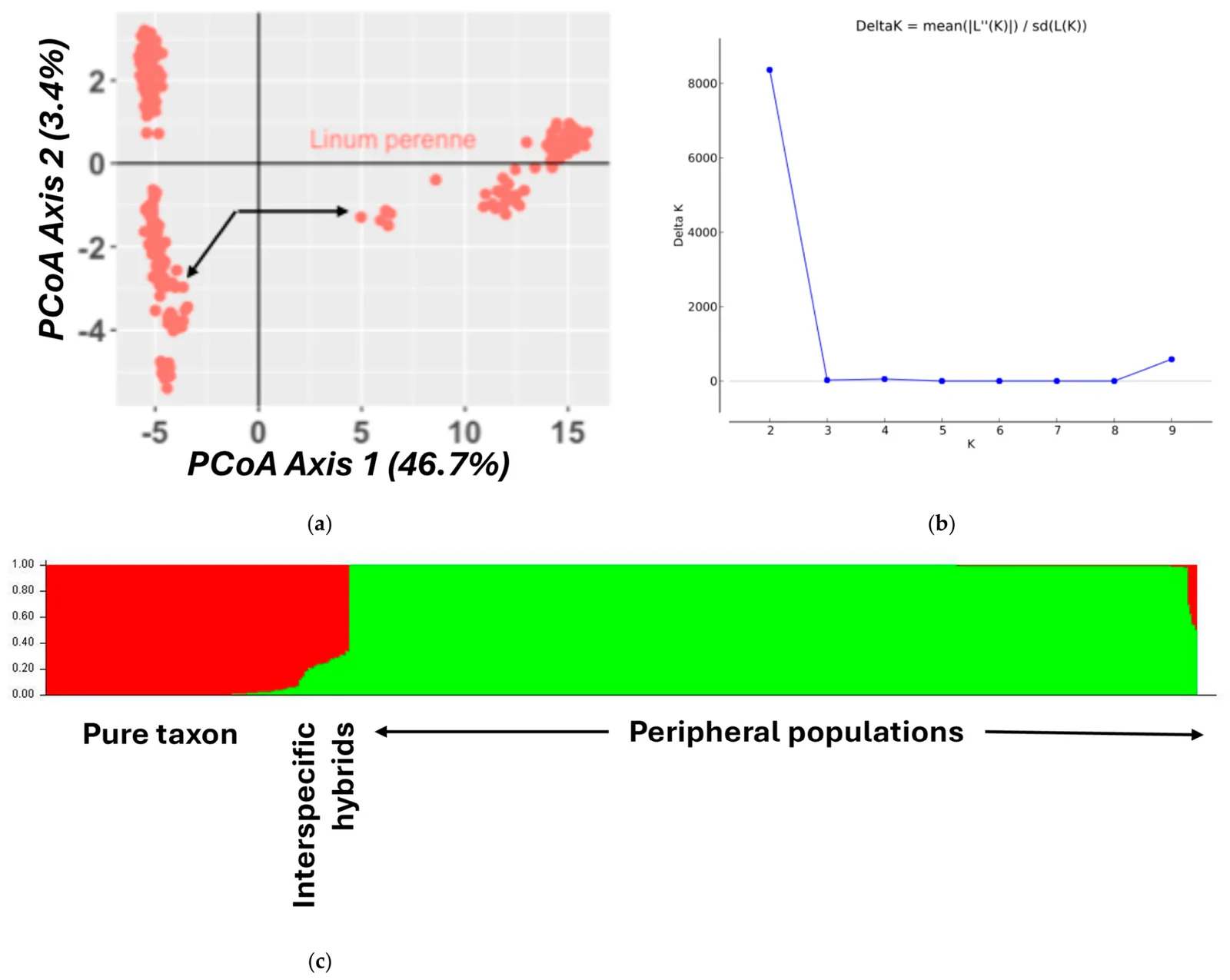
Genetic structure and diversity of perennial flax, *Linum perenne* (Group 4), for accessions (wild or cultivated populations, cultivars) and their genotypes (n = 1–20/accession) in sampled single-nucleotide polymorphisms (SNPs). (**a**) Principal coordinate analysis (PCoA) plot for the first two coordinates (PCoA 1 and PCoA; arrows denote the peripheral populations); (**b**) estimation of K based on the filtered dataset using ΔK, following Evanno et al. [54]. Generated graph was produced via Structure Harvester [55]. Highest ΔK occurs at K = 2, 9; (**c**) single-line Q-plot denoting the separation of the filtered dataset for K = 2, as determined by STRUCTURE analysis.

In the Group 4 analysis of *L. perenne* accessions and genotypes, the percent contribution to the PCoA axis peaked at zero (46.7%), with all eigenvalues being positive. For the 2716 *L. perenne*-identified SNPs in Group 4 (309 genotypes), the PCoA also showed three clusters (Figure 7a), following a similar pattern for all species (Group 1), as well as the other two species, *L. austriacum* (Figure 3a) and *L. lewisii* (Figure 5a). Most genotypes are contained in the two primary clusters for the taxon, with a smaller cluster containing the “peripheral populations?” grouping within *L. perenne*. The first PCoA dimension (Axis 1, PCoA1) describes 46.7% of variation between *L. perenne* individuals (Group 4), while the second dimension (Axis 2, PCoA2) describes 3.4% (Figure 7a). Geometric distances in the PCoA between genotypes reflect genetic distances among genotypes [60].

Genotypes within the pure taxon *L. perenne* groups were six wild population derivations collected in various locations within Hungary (Orgovany: PI 650324, 19F-535; no locations: PI 650325, 19F-734; CN 107283, 19F-982; PI 650323, 19F-1518) or unknown locations (CN 19179, 19F-1673; CN 107282, 19F-1678) (Supplemental Table S1). The pure species taxon group with both positive PCoA Axis 1 and 2 values includes wild species collections only from Hungary (locations unknown, CN 107283; PI 650324; CN 107283; Orgovany, PI 650325), with the exception of one unknown accession (CN 107282). In contrast, the pure species taxon group with positive PCoA Axis 1 but negative PCoA Axis 2 values was from wild population collections of different Hungarian origins (PI 650323), as well as wild material of unknown origin (CN 19179; Figure 7a; Supplemental Table S1). One population had genotypes in both pure taxon groupings, i.e., PI 650324.

Accessions within the “peripheral population” group were two populations originating from the Lake Balaton area, north coast, Hungary (near Vorosbereny, PI 650327, 19F-337; PI 650326, 19F-754), Moldova (Ames 21222, 19F-499), naturalized populations outside of the native species range in Sawtooth National Recreation Area, Idaho, USA (PI 650328, 19F-356), near the Bureau of Land Management building, Boise, Idaho, USA (PI 650329, 19F-375), in Oregon, USA (Ames 31374, 19F-515), two populations from South Dakota, USA (PI 445972, 19F-1001; PI 445972, 19F-1094), two seed lots of a commercial cultivar, ‘Blue Flax’ (19F-932 and 19F-1039, American Meadows Seed Co., Shelburne, VT, USA), an unnamed cultivar from Alaska Wildflowers, North Pole, Alaska, USA (19F-1040), and a German cultivar, ‘Himmelszelt’ (CN 19024, 19F-1778), along with wild populations of unknown origin (CN 107261, 19F-1647; CN 107256, 19F-1792; CN 107270, 19F-1809), all of which were of unknown origin (Supplemental Table S1). As noted, some of the peripheral population genotypes are potentially putative interspecific hybrids and/or genotypes showing possible admixture, since they shared SNPs from both groups in the STRUCTURE analysis (Figure 7c).

**Figure 8:**
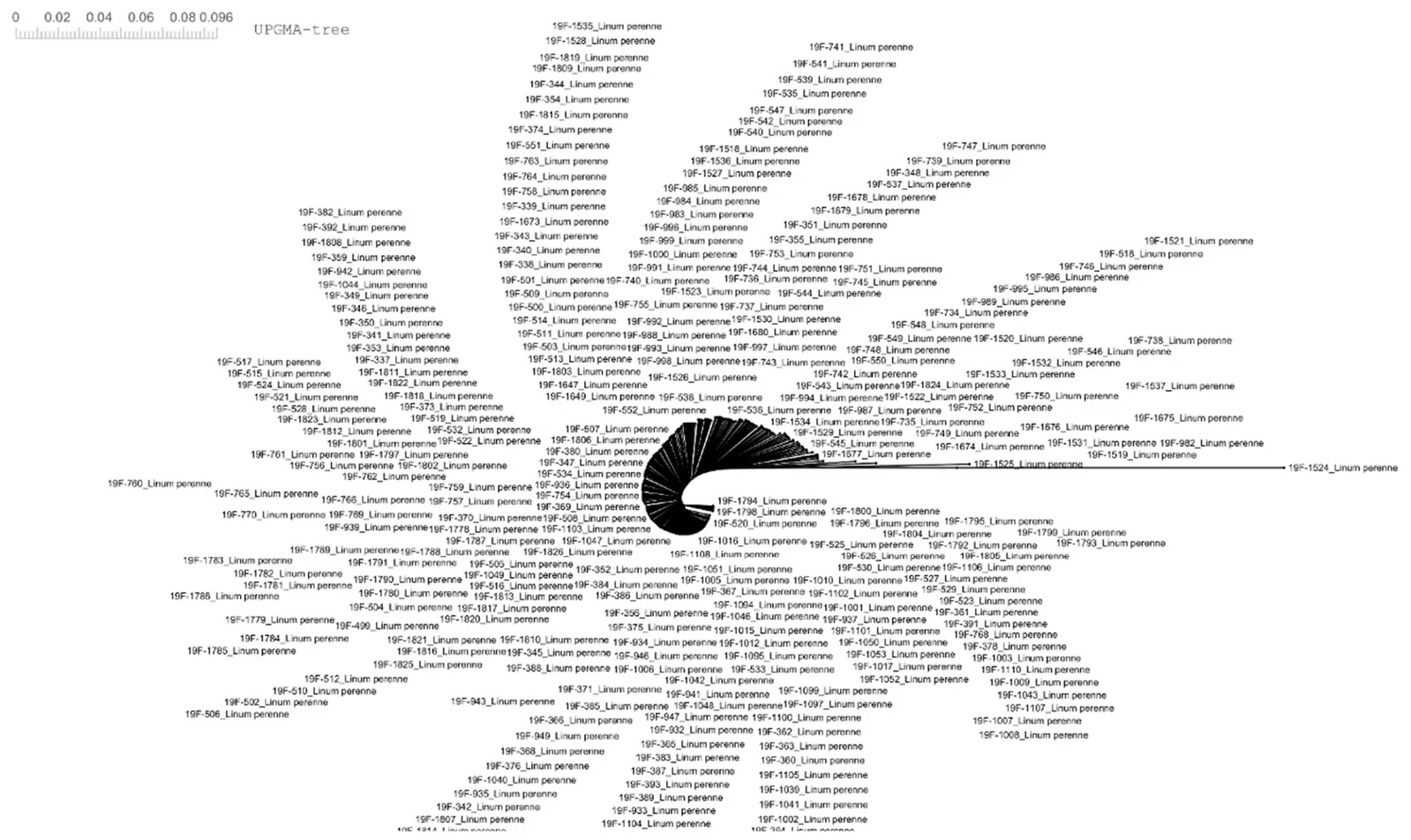
Unrooted phylogenetic tree (SplitsTrees) from single-nucleotide polymorphism (SNP) data for taxa in the perennial flax species, *Linum perenne* (Group 4).

Branching in the phylogenetic splits tree network (Figure 8) for the complete taxon dataset of Group 4 (*L. perenne*) complements the corresponding PCoA clustering patterns for the dataset (Figure 7a). Distribution of the *L. austriacum* (Group 2) and *L. lewisii* (Group 3) genotypes in the phylogenetic splits tree network also formed an unusual scorpioid curve shape (Figure 4 and Figure 6, respectively). Genotypes 19F-1524 and 19F-1525 (Table 1), constituting the long tail in Figure 8, are the pure taxon, *L. perenne* from USDA-GRIN accession PI 650323, originally collected from a wild population in Hungary. Most other pure taxon genotypes (19F-535 to 19F-753, 19F-982 to 19F-1000, 19F-1518 to 19F-1537, and 19F-1673 to 19F-2680; Supplemental Table S1) were also close to the tail or moving towards the beginning of the scorpioid curve. Genotypes within the “peripheral populations?” PCoA cluster (Figure 7b) were primarily intermixed at the base or beginning of the scorpioid curve in the phylogenetic splits tree network (Figure 8) and included *L. perenne* 19F-337 to 19F-534; 19F-754 to 19F-949; 19F-1001 to 19F-1110; 19F-1647; 19F-1649; and 19F-1778 to 19F-1826 (Supplemental Tables S1 and S2). These “peripheral population” genotypes were from a variety of sources and wild populations, including USDA-GRIN (PI 650327, PI 650328, PI 650329, PI 650326, PI 445972; Ames 21222, Ames 31374), CN (CN 10726, CN 19024, CN 107256, CN 107270), and commercial sources (AK Wildflower; ‘Blue Flax’). The genotypes within each of these accessions were in consensus, being all classified into the same groupings (pure taxon vs. “peripheral population”).

### 3.2. Structure

- Group 1.

For the three perennial flax species in this study, STRUCTURE 2.3.4 calculated the peak at ΔK = 2, with a small shoulder at ΔK = 4 (Figure 1b). The ΔK = 2 matches the separation of the potentially “peripheral populations” group of the *Linum* species from the individual taxa and mirrors the STRUCTURE bar plot (Figure 1c). The ΔK = 4 matches the separation of the putative interspecific hybrids and/or genotypes with possible admixture cluster from the clusters *of L. lewisii, L. perenne*, and *L. austriacum*. Both values are consistent with the PCoA analysis of the three perennial flax species (Figure 1a).

- Groups 2–4.

In all cases for Groups 2–4, STRUCTURE analyses for all of the individual taxa peaked at ΔK = 2 for *Linum austriacum* (Group 2; Figure 3b), *L. lewisii* (Group 3; Figure 5b), and *L. perenne* (Group 4; Figure 7b), with corresponding STRUCTURE bar plot divisions (Figure 3c, Figure 5c and Figure 7c, respectively). Thus, all are consistent with the corresponding PCoA analyses, where the individuals that are potentially hybrids are clustered separately from others of a species, forming two distinct groups of pure taxa and a “peripheral population”.

### 3.3. Analyses of Molecular Variance (AMOVAs)

In the Group 1 AMOVA for all three perennial species, there was greater genetic variation within (86%) than among populations (14%) (Table 1). A low ɸPT = 0.144 (p < 0.001; Table 1) was found for Group 1. Group 2 AMOVA for *L. austriacum* also had the highest genetic variation within (95%) rather than among populations (5%), with a correspondingly lowest ɸPT = 0.053 (*p* < 0.001; Table 1) for all individual species groups. In the Group 3 AMOVA for *L. lewisii*, the highest genetic diversity was likewise within (94%) rather than among populations (6%), with a calculated ɸPT = 0.064 (*p* < 0.01; Table 1). Group 4 AMOVA for *L. perenne* had a greater level of genetic variation within (89%) than among populations (11%; Table 1), with a calculated ɸPT = 0.107 (*p* < 0.001). Thus, for all groupings of perennial flax species under examination, within-population genetic variation was significantly higher than among populations.

## 4. Discussion

In this study, we examined genetic diversity and genetic structure using the DArTseqLD GBS approach within and among accessions (populations or seed lots) in three perennial flax species under domestication at the University of Minnesota breeding program [19,20,21,22,23,24,25,26,27], i.e., *Linum austriacum, L. lewisii*, and *L. perenne*. Similar to previous *Linum* genetic diversity studies [13,14,46,61], our germplasm was from various world seed bank sources, all of which have already been extensively characterized, bred, and selected for a variety of agronomic and horticultural traits, e.g., oilseed, fiber, pollinator services, cut flowers, and herbaceous perennials [19,20,21]. Our research with the 843 genotypes (with a minimum call rate of 90%) in the tripartite perennial taxa generated 9804 polymorphic SNP markers for the three perennial flax species (Group 1), similar to previous reports for low-density SNPs in annual flax, *L. usitatissimum* (3230 SNPs generated by GBS) [8], and other plant eudicot species [62,63]. These SNPs differentiated the tripartite perennial flax taxonomic taxa.

As expected, the number of SNPSs detected using DArTseqLD is much lower than the five high-density genetic maps of *L. usitatissimum*, containing > 100,000 SNPs (https://www.ncbi.nlm.nih.gov/datasets/genome/?taxon=4006, accessed on 9 August 2025), or that of *L. lewisii* (https://www.ncbi.nlm.nih.gov/datasets/genome/GCA_034768395.1/, accessed on 9 August 2025) [13,14]. The Canadian National Genebank for plant genetic resources for food and agriculture, Plant Gene Resources of Canada, core flax collection [28] contained ~1.7 million SNPs [9,10,11,12], contributing greatly to the understanding of the genetic structure of annual flax and marker association with yield-related traits. This drastic difference in SNPs between annual and perennial flax is more than likely due to the use of the annual flax genome (CDC Bethune) for SNP discovery. A comparative base genome from a perennial species was unavailable and not searchable at the time of this study and analyses (the *L. lewisii* genome was published thereafter). Another discrepancy between perennial and annual species of flax is the base chromosome numbers, although all four species are diploid. Cultivated annual flax is 2*n* = 2*x* = 30, whereas the three perennial species are diploid with a different base number, 2*n* = 2*x* = 18 [47]. This variation in chromosomes could mean that the perennial species genome varies from the annual flax genome (368 Mb, 85% captured). Indeed, the perennial *L. lewisii* genome is 643 Mb in size [13,14], several orders of magnitude greater than annual flax. Likewise, high-density (HD) GBS instead of LD (as used in this study) would likely generate hundreds of thousands of SNP markers. This will be pursued in future studies using the foundational information generated by LD SNP generation.

The PCoA genetic clustering results (Group 1) showed that high levels of variation existed among all three species (40.3%), with separation into two distinct groups, with complementary STRUCTURE ΔK = 2, 4, and q-matrix and bar plots distinguishing between species (Figure 1). Resulting AMOVA analyses supported that genetic variation occurred among genotypes (67%; Table 1), indicating that there was high genetic diversity among the different *Linum* species. These levels of genetic diversity are fundamental for continued species evolution and adaptation, as well as ensuring the divergent genetic makeup of *Linum* accessions within germplasm banks [31,61]. According to the STRUCTURE analysis, each Group 1 perennial species shows enough genetic variation among genotypes to warrant separation into two clusters. Population variation (13%) indicates that there is lower genetic variation observed within a single species, which is supported by the generated phylogenetic trees showing little clustering thereof.

**Figure 9:**
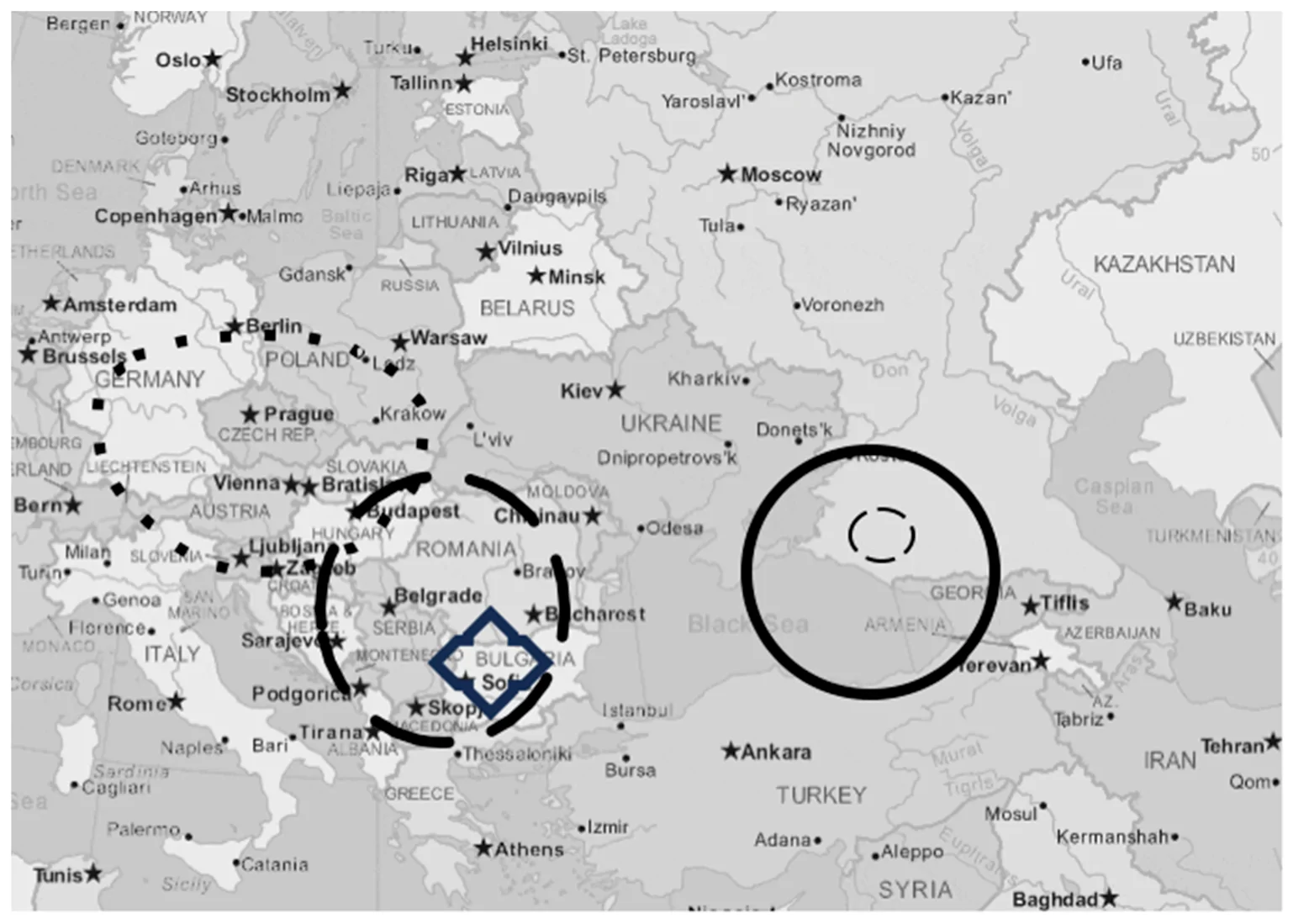
Map of putative *Linum austriacum* Centers of Origin across Eurasia (https://www.guideoftheworld.net). Key: solid circle: putative eastern Center of Origin (pure taxon); dashed circle: putative western Center of Origin (pure taxon); dotted circle: peripheral population; diamond: outlier peripheral population.

In each species’ SNP analyses (Groups 2–4), the separation of each pure taxon into two distinct groups was found (Figure 3a, Figure 5a and Figure 7a). Accessions of wild populations collected within the species’ ranges provided the predominant genotypes in the two groupings for each taxon. Specifically, in Group 2, *L. austriacum*, using the 1199 species-specific SNPs, we propose that there may be two distinct geographical putative Centers of Origin and/or diversity for this taxon based on the genetic structure of the accessions. The eastern core population with both positive PCoA Axis 1 and 2 values (Figure 3a; Supplemental Table S1) includes wild species populations in eastern Europe, specifically the Russian Federation’s central Caucasus regions of Yankuli (Stavropol hills), Elista, Kalmyk ARR, southeast of Stavropol (Beshpagin Sands), Marfivka, and Kherson Oblast, Ukraine, as well as Jil, Armenia (Figure 9). West of this core population is a second one for *L. austriacum,* all of which had positive PCoA Axis 1 but negative PCoA Axis 2 values (Figure 3a; Supplemental Table S1). This core population included collections from more westerly regions of eastern Europe, specifically Hungary (near Tatatszentgyorgy and Orgovany, the Great Hungarian Plain between Duna (Danube) and Tisza Rivers, Kiskunsag, Bács-Kiskun), Bulgaria (north of Albena as well as near Kaliakra, both of which are close to the Black Sea (Figure 9)). In one instance, a minority of genotypes within a Russian Federation population southeast of Stavropol (PI 440472) were in the western core population, while the remaining genotypes matched the eastern core population (Figure 9). One accession had no country of origin and was discounted. The separate geographical groupings of the pure *L. austriacum* taxon are similar to Vavilov’s determination of two putative geographic Centers of Origin for annual flax, *L. usitatissimum* [64], which was later confirmed to be Central Asia and the Mediterranean region in the primary gene pool [65,66]. Future research will examine additional genotypes within these *L. austriacum* pure taxon groups, as well as wider and increased sampling of the species’ germplasm in other gene banks and other *Linum* species, to test the rigor of the putative two Centers of Origin for this taxon. Thus, we present these as putative Centers of Origin, pending future analyses of an increased germplasm bank.

**Figure 10:**
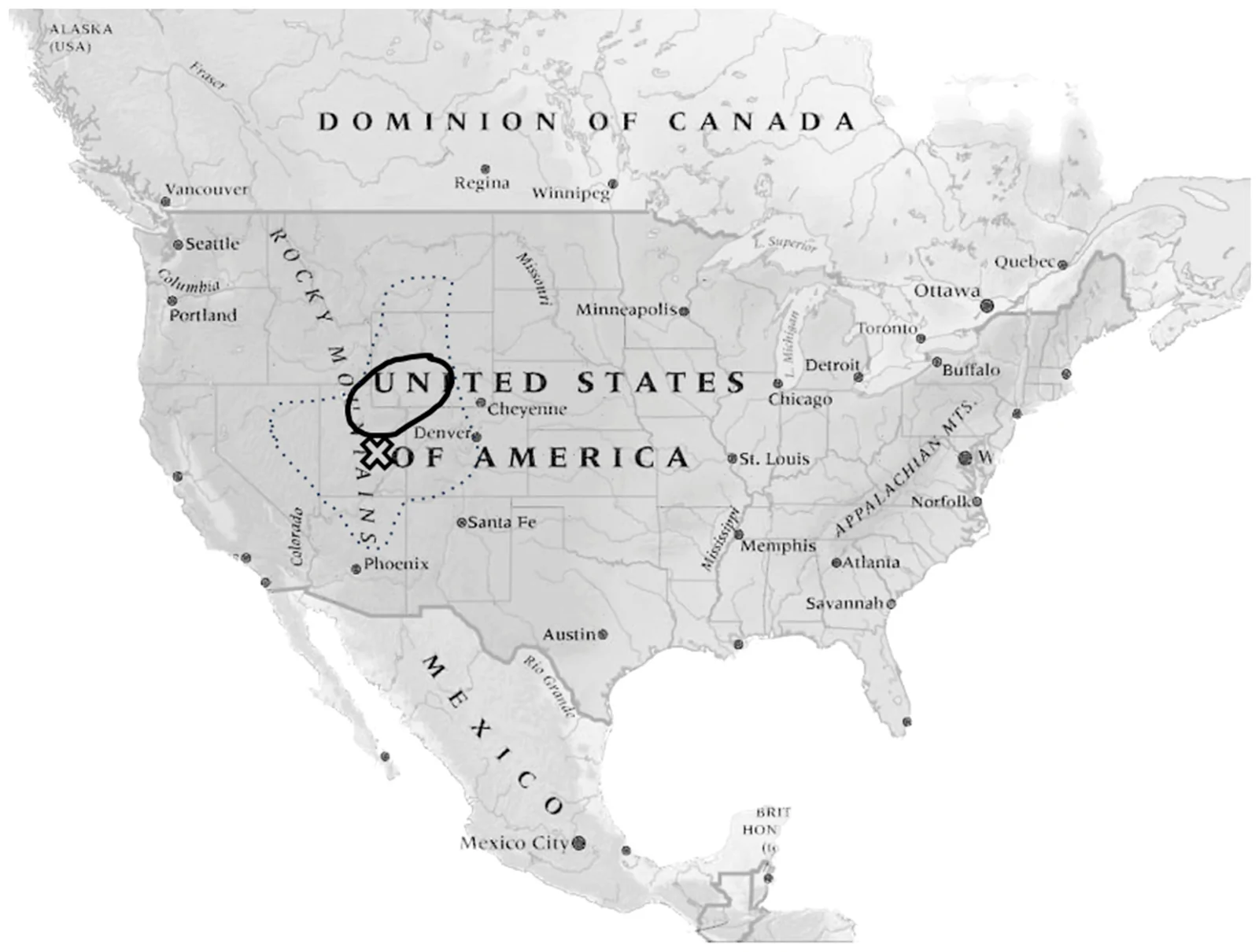
Map of proposed *Linum lewisii* Centers of Origin (using the map https://www.guideoftheworld.net/usa-canada-mexico-map, accessed on 9 August 2025). Key: solid line: northern putative Center of Origin (pure taxon) in the states of Wyoming and Utah; dotted line: southern putative Center of Origin (pure taxon) covering the states of Wyoming, Utah, Nevada, Arizona, Colorado, and Montana; X: outlier peripheral population location.

In Group 3, *L. lewisii*, using the 90 species-specific SNPs, we propose that there may be two distinct geographical putative Centers of Origin for this taxon (Figure 10) based on the genetic structure of the accessions. The central core population, with both positive PCoA Axis 1 and 2 values (Figure 5a; Supplemental Table S1), includes wild species populations in the states of Utah and Wyoming. A second core population has a much larger geographic spread, completely surrounding the other core population; these are accessions with positive PCoA Axis 1 but negative PCoA Axis 2 values (Figure 5a; Supplemental Table S1). These populations and cultivars (‘Maple Grove’) originated in the states of Wyoming, Utah, Nevada, Arizona, Colorado, and Montana (Figure 10). Many of the *L. lewisii* populations had an unknown origin (Supplemental Table S1) and were not included in any of the putative Centers of Origin for the pure taxon. The one “peripheral population” occurred in the state of Utah, slightly overlapping with both core populations (Figure 10). We acknowledge the limited sampling of *L. lewisii* germplasms whose native range extends from Mexico into northern Canada. Since our sampling is limited for this taxon, based on the germplasm sources used in the breeding program, the putative Centers of Origin are initial proposals that will require future extensive sampling of the entire *L. lewisii* germplasm collections throughout North America to make a final revision. This research awaits further study.

**Figure 11:**
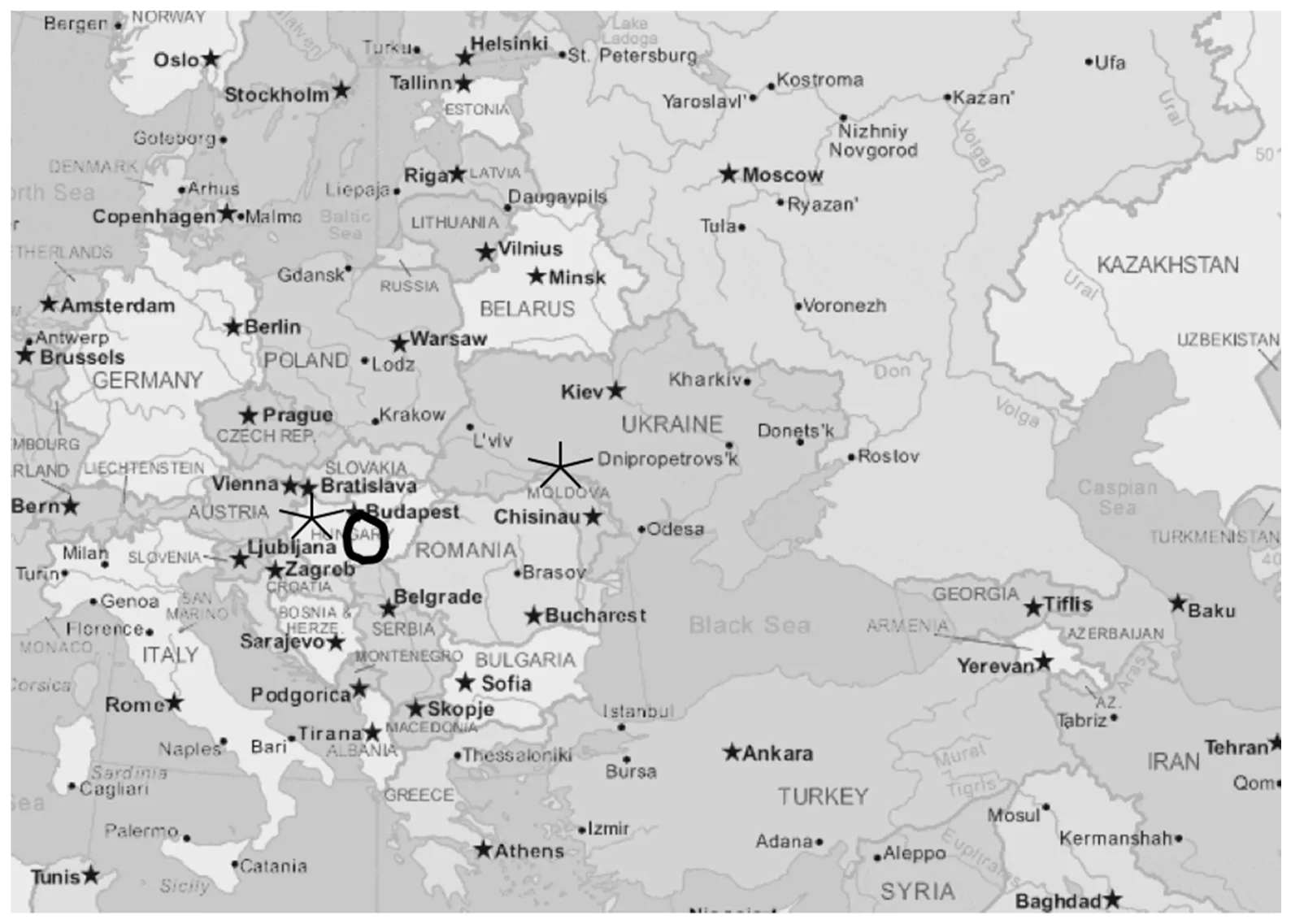
Map of putative *Linum perenne* Centers of Origin (https://www.guideoftheworld.net). Note: does not include naturalized populations in N. America. Key: solid circle: eastern putative Center of Origin (pure taxon); circle: western putative Center of Origin (pure taxon); large unfilled stars: peripheral populations.

*Linum perenne* is in the tertiary *Linum* gene pool [65,66]. Despite the large set of germplasms sequenced, in Group 4, *L. perenne*, with its 2716 species-specific SNPs, there is only one defined core population (Figure 11) in Hungary. This is due to the multiplicity of naturalized *L. perenne* populations in North America (Supplemental Table S1) that were discounted. Two outlier peripheral populations occurred in Moldova and near Lake Balaton, southwest of Budapest, Hungary (Figure 11). Obviously, given the low number of populations collected in the wild of Europe in our study, future analyses will need to be conducted to expand the geographic region for this core population, since the taxon is widespread from eastern Europe into Asia [32]. This will provide evidential data for potential reorganization of the actual, rather than putative, Centers of Origin for this species.

The occurrence of a distinct “peripheral population” cluster in the Group 1 germplasm pool, which extended beyond the pure taxa clusterings, included more *L. perenne* (226/309 = 73.1% of species total) than *L. lewisii* (95/276 = 34.4%) or *L. austriacum* (90/264 = 34.1%) genotypes (Supplemental Tables S1 and S2). Since these genotypes were distributed across our tested *Linum* germplasm sources (USDA GRIN, PI, or AMES; Canadian germplasm, CN; Alaska wildflowers; commercial sources; Supplemental Table S1), their genetic makeup appears to be widespread throughout perennial Linum taxa. Many of these populations and genotypes were from arboreta, were commercial cultivars, of otherwise unknown origin (Supplemental Table S1), or were naturalized populations outside of the native range(s), which could explain their diverse genetic makeup, distinguishing them from the pure taxa. In some instances, there have been reports of sympatric *Linum* species within their native ranges, e.g., *L. austriacum* was found in close proximity to *L. tenuifolium* L. and *L. corymbulosum* Reichenbon in the Crimean Peninsula, Sevastopol, Ukraine [28]. Additional reports of interspecies sympatry have included Türkiye [37]. Since the three perennial flax species studied herein all belong to cytogenetic Group I in *Linum* (2*n* = 2*x* = 18), all are capable of peripheral populationization [18]. They could have been planted sympatrically with other perennial flax species, which could have provided the opportunity for gene exchange. Likewise, since at least *L. perenne* and *L. austriacum* have subspecies, these may be misidentified accessions. For example, there are three *L. austriacum* subspecies, i.e., *L. austriacum* ssp. *austriacum*, *L. austriacum* ssp. *squamulosum*, and *L. austriacum* ssp. *collinum* [36]. Future research will clarify whether these accessions are of peripheral population origin. In a few instances, the “peripheral population” constituents were directly from derivatives of wild population collections in the species’ native ranges; as such, these could be seed lot mix-ups, misidentification of the correct taxa, or have evolved into new subspecies not previously identified. Most likely, none of these accessions would be advanced inbred lines or families, due to the unknown levels of selfing/outcrossing in *L. lewisii* (homostylous and self-compatible [13,44] or heterostylous [46]), or the presence of heterostyly and SI [34] in *L. austriacum* [61] and *L. perenne* [31]. Thus, the reason(s) for SNP differentiation in genetic sequences among these “peripheral population” genotypes are unknown at present. Distinct geographic source differentiation within *L. usitatissimum* was attributed to multiple centers of flax variation [12,38], and this, likewise, could be the case for the tripartite perennial flax studied herein. They could also contain any of the tetraploid (2*n* = 4*x* = 36) European variants within the *L. perenne* complex [31] or constitute peripheral populations with one or more *Linum* species (annual, biennial, or perennial) not included in this study. Possible gene flow from peripheral populationization among wild species relatives has been theorized in annual flax, *L. usitatissimum*, dependent on species’ sympatry and ploidy- and cross-compatibility [3]. Future research will focus on this question to determine any potential corollary species’ SNP alignment with this grouping. In particular, we would focus studies on cross-compatible species for *L. austriacum* and *L. perenne*, as outlined by [50] cf. Figure 9.

The existence of three genotypes in *L. austriacum* (19F-224, PI 650296-1; 19F-245, PI 650297-6; Figure 5a and Figure 9) and *L. lewisii* (19F-1577, Ames 32565-3; Figure 1a and Figure 10; Table 1) outside of their species’ taxa SNP alignment, categorized as “outlier peripheral populations”, is an additional anomaly in this study. When compared with the other tested genotypes within these accessions (PI 650296, plants 2–20; PI 650297, plants 1–5, 7–20; Ames 32565, plants 1–2, 4–20), all three “outlier peripheral population” genotypes (PI 650296, plant no. 1; PI 650297, plant no. 6; Ames 32565, plant no. 3) were outliers in SNP alignments. Previous research on native *L. austriacum* populations in Iran showed that 6/100 genotypes were from outside of their native populations [61], potentially from genetic drift, gene flow, etc. These are most likely genetic mix-ups within these three accessions: potential seed contaminants during seed harvest, cleaning, packaging, or storage in the respective germplasm banks. Such mix-ups are not uncommon in commercial agronomic and horticultural seed [67,68] or vegetative propagule production, and molecular markers are frequently used for purity checks in addition to standard physical purity checks [69]. Maintaining seed purity is a constant challenge for germplasm bank curators. This is particularly true when variants, sharing phenotypes similar to other genotypes in the same seed lot, cannot be distinguished in simple growouts or visualized during seed cleaning processes. Since their SNP alignment differentiated them from the “peripheral population” genotypes, their genetic makeup may constitute differing *Linum* species. Future research will focus on these three genotypes to determine potential SNP sources for these genetic variants.

SplitsTree cluster analyses of Group 1 showed N. American *L. lewisii* with an extended tail separated from the other two Eurasian species, *L. austriacum* and *L. perenne* (Figure 2). Interestingly, *L. lewisii* and *L. austriacum* were adjacent to each other whilst *L. perenne* was closest to only *L. austriacum*. This matches the regional genetic relationships from 149 randomly amplified polymorphic DNA (RAPD) markers previously reported [38] for *L. usitatissimum,* wherein the eastern European and Asian accessions were more closely aligned with N. American accessions, whereas western European ones were clustered further away. In our study species, *L. austriacum* is native to Türkiye and Iran, whereas *L. lewisii* is N. American and *L. perenne* is European.

The species-specific Group 2 (*L. austriacum*) phylogenetic SplitsTree cluster analyses showed a unique and unusual scorpioid curvature to the distribution of the genotypes (Figure 4). Typically, most genotypes within an accession had similar SNP configurations, such that they were adjacent in the SplitsTree. The two “outlier peripheral population” genotypes were at opposite ends of the scorpioid curve (Figure 4; box highlights), despite being adjacent in the PCoA (Figure 3a). Thus, while their SNPs distinctly differentiated them from the pure *L. austriacum* taxon, the similarity ceases thereafter. Previous studies of *L. austriacum* found significant genetic differentiation among populations and accessions with either ISSRs or EST-SSR markers [61]. The constituent “peripheral population” grouping’s relationship with the pure *L. austriacum* taxon is unclear and will require further SNP analyses and GWAS evaluations to determine why these remain a distinct grouping. Reports of varying genome sizes in the species’ wild populations in Iran, e.g., 1.88 pg to 3.17 pg [70], could account for SNP differences, as well as reports that style morphs (long- and short-styled) were responsible for different genome sizes [71]. Thus far, cpDNA variation has been assessed (https://www.ncbi.nlm.nih.gov/bioproject/768027; https://www.ncbi.nlm.nih.gov/bioproject/768026, both accessed on 9 August 2025), along with shotgun sequencing, to assess the phylogenetic relationships of 16 species within four botanical sections of the *Linum* genus (https://www.ncbi.nlm.nih.gov/bioproject/956870, accessed on 9 August 2025) [38]. DArTSeqHD analysis will also be employed to provide 100,000s of SNPs for genome sequencing. Likewise, future molecular analyses will be incorporated with phenotypic studies and incorporate style morph effects.

Significantly different levels of genetic variation occurred in *L. austriacum* populations than previously reported. Whereas 43% within- and 57% among-population total genetic variability had been found for *L. austriacum*, with ɸPT = 0.566 [61], in the present study, the levels were 95% for within- and 5% for among-population variability, with ɸPT = 0.053 (Table 1). These differences could be attributed to the germplasm sampling of seed-lot populations within seed germplasm banks, which represent a larger and more diverse array of genetic variation than previous studies [72]. Regardless, the high levels of genetic variability found within this species will provide ample opportunities for the breeding and selection of perennial flax for numerous crop domestication purposes [19,20,21,22].

The species-specific Group 3 (*L. lewisii*) phylogenetic SplitsTree cluster analyses (Figure 6) repeated the scorpioid configuration found in *L. austriacum* (Figure 4). Again, most of the n = 1–20 genotypes in each accession had similar SNP configurations, aligning them adjacent to each other in the SplitsTree. The sole “outlier peripheral population” genotype was at the beginning of the scorpioid curve (Figure 6; highlighted with a box), and was found with one of the *L. austriacum* also in this grouping. The “peripheral populations?” group was scattered throughout the SplitsTree, along with the pure *L. lewisii* taxon, indicating similarities in phylogeny despite the differentiated SNP configurations. In previous SNP research with *L. lewisii* (actual accession is unknown), nine SCAR or sequenced characterized marker groups were closely associated with *L. marginale*, both of which were then subgrouped with *L. flavum* and annual winter flax [73]. The wide geographic distribution, phenotypic differentiation, and genetic diversity of Lewis flax (*L. lewisii*) in western North America [13,14] match the findings in the SNP analyses. Correspondingly, “extensive” phenotypic and chemotypic trait variations in 35 wild populations, as well as *L. lewisii* ‘Maple Grove’, were recently reported. Analyses of the identified phenotypic (flowering indeterminacy, seed mass, and stem number) and/or chemotypic (alpha-linolenic acid) traits will be important in the continued future domestication of Lewis flax for oilseed, fiber, pollinator, and ornamental crop uses and will be aided by its small genome size of ~600 Mb [47].

Levels of genetic variation (AMOVA) were 94% within and 6% among populations at a ɸPT = 0.064 for *L. lewisii* (Table 1), similar to values found for *L. austriacum*. This is the first report of ɸPT for the species, so we have no comparative studies of genetic variation. Chloroplast DNA (https://www.ncbi.nlm.nih.gov/nuccore/HM590309.1 (accessed on 9 August 2026)) and ribosomal RNA sequences (https://www.ncbi.nlm.nih.gov/nuccore/MK066718.1 (accessed on 9 August 2026)), along with internal/external transcribed sequences (ITS, https://www.ncbi.nlm.nih.GOV/nuccore/KT453459.1 (accessed on 9 August 2026); ETS, https://www.ncbi.nlm.nih.gov/nuccore/KT453350.1 (accessed on 9 August 2026)), have been reported for *L. lewisii*. Additionally, six RNA-Seq of *L. lewisii* ‘Maple Grove’ (https://www.ncbi.nlm.nih.gov/sra/SRX21081773[accn] (accessed on 9 August 2026), etc.), two Illumina HiSeq 2000 or 2500 paired-end sequencing (https://www.ncbi.nlm.nih.gov/sra/ERX2099433[accn] (accessed on 9 August 2026), https://www.ncbi.nlm.nih.gov/sra/ERX5309872[accn] (accessed on 9 August 2026) of Norwegian arctic vascular flora, respectively), and one OmniC for chromosome-scale scaffolding of the genome (https://www.ncbi.nlm.nih.gov/sra/SRX21081767[accn] (accessed on 9 August 2026)) have been performed at North Dakota State University, along with whole-genome shotgun sequencing with random fragmentation at the University of Alberta (https://www.ncbi.nlm.nih.gov/sra/SRX716950[accn] (accessed on 9 August 2026)), all of which produced a genome size of 5.9–6.0 Gb. However, this is the first nuclear DNA GBS reported to date.

Overall, levels of genetic variation among a species’ individuals support the positive identification of accessions as specified by USDA GRIN for the *Linum* species *L. austriacum, L. lewisii,* and *L. perenne*. The three perennial *Linum* species had a majority of their genetic diversity among the three perennial species, indicating that gene flow between species would be a potential source of genetic variation in lacking perennial populations. Researchers have been studying the peripheral populationization of only select *Linum* species, with very few having been successful. Further studies with perennial species would be needed, taking into account the different ploidy levels, sexual compatibility, and other factors impacting hybridization potential. We aim to continue to use our method of ideotype breeding to capture our top traits for oilseed, cut flower, and garden ornamental flax.

## Figures and Tables

**Table 1:** Analyses of molecular variance (AMOVA) for the complete dataset of the three perennial *Linum* species and for each individual species: *L. austriacum, L. lewisii,* and *L. perenne*.

Groups	Partitioning	df	SS	MS	Est. Var.	%	ɸPT, Significance
Group 1 (All 3 species)	Among Pops	2	10,899.6	5449.8	19.0	14%	0.144 ***
	Within Pops	840	95,093.0	113.2	113.2	86%	
	Total	842	105,992.7		132.2	100%	
Group 2 (*L. austriacum*)	Among Pops	1	842.7	842.7	6.2	5%	0.053 ***
	Within Pops	259	28,793.4	111.2	111.2	95%	
	Total	260	29,636.1		117.4	100%	
Group 3 (*L. lewisii*)	Among Pops	1	84.2	84.2	0.6	6%	0.064 **
	Within Pops	271	2409.6	8.9	8.9	94%	
	Total	272	2493.8		9.5	100%	
Group 4 (*L. perenne*)	Among Pops	1	3076.0	3076.0	23.5	11%	0.107 ***
	Within Pops	307	60,462.6	196.9	196.9	89%	
	Total	308	63,538.7		220.5	100%	

*** *p* < 0.001, ** *p* < 0.01.

## Data Availability

Data are unavailable due to intellectual property restrictions.

## Supplementary Materials

The following supporting information can be downloaded at: https://www.mdpi.com/article/10.3390/genes17080978/s1, Supplemental Table S1: Perennial *Linum* tested from the USDA GRIN Accession (PI or Ames; GRIN-Global, 2022) codes and plant #, Identification code for analyses, passport Data (collection location), and species confirmation or interspecific hybrid designation; Supplement Table S2: Perennial *Linum* from the “hybrid” cluster with USDA determined species designation along with USDA accession code and identification code for analyses. Yellow highlights denote genotypes sorted for positive Ys for the top pure taxon cluster; all negatives would be in the lower cluster. Sheet 1, All three species hybrid cluster; Sheet 2: *Linum austriacum* nonhybrids; Sheet 3: *Linum lewisii* nonhybrids; Sheet 4: *Linum perenne* nonhybrids; Sheet 5: Reference sheet, all genotypes; Sheet 6: PCoA with all genotypes; Sheet 7: *Linum lewisii*; Sheet 8: *Linum perenne*; Sheet 9: *Linum austriacum*. Or from the Data Repository for the University of Minnesota (DRUM) site: https://hdl.handle.net/11299/166578 (accessed on 17 August 2026).

## Abbreviations

The following abbreviations are used in this manuscript:

a.i.	Active ingredient
AMOVA	Analysis of Molecular Variance
CLF	Constant liquid feed
CN	Plant Gene Resources of Canada
DArT	Diversity Arrays Technology
GRIN-Global	Germplasm Resource Information Network Global collection
HPS-HID	High-pressure sodium high-intensity discharge
LD	Low density
PI	Principal identifier
PCoA	Principal component analysis
QTL	Quantitative trait loci
SNP	Single-nucleotide polymorphism
UPGMA	Unweighted pair group method of analysis
USDA-GRIN	U.S. Department of Agriculture–Germplasm Resources Information Network
