# Supplementary material for: Genetic Structure of the Perennial Flax Trifecta: Linum austriacum, L. lewisii, L. perenne
Source: Genes (Basel). 2026 Aug 20;17(8):978. doi: 10.3390/genes17080978 (PMC13511843; doi:10.3390/genes17080978)
Supplement: Supplementary file 1 [file genes-17-00978-s001.zip › genes-4431642-supplementary.pdf]

Supplemental Tables.

Supplemental Table S1. Perennial *Linum* tested from the USDA GRIN Accession (PI or Ames; GRIN-Global, 2022) codes and plant #, Identification code for analyses, passport Data (collection location), and species confirmation or interspecific hybrid designation.

Linum austriacum			
**USDA GRIN Accession (PI or Ames; GRIN-Global, 2022) or Plant Gene Resources of Canada Genetic Resource Information Network (CN) codes - Plant #**	**Identification code for analyses**	**Passport Data (collection location)**	**SNP confirmation for species designation (x) or interspecific hybrid**
PI 502405-1	19F-1	Steppe, Askania-Nova Preserve, Ukraine	x
PI 502405-2	19F-2		x
PI 502405-3	19F-3		x
PI 502405-4	19F-4		x
PI 502405-5	19F-5		x
PI 502405-6	19F-6		x
PI 502405-7	19F-7		x
PI 502405-9	19F-8		x
PI 502405-10	19F-9		x
PI 502405-11	19F-10		x
PI 502405-12	19F-11		x
PI 502405-13	19F-12		x
PI 502405-16	19F-14		x
PI 502405-17	19F-15		x
PI 502405-19	19F-16		x
PI 502405-20	19F-17		x

PI 502410-1	19F-18	West, 250km of Elista, Kalmyk ARR Russian Federation	x
PI 502410-2	19F-19		x
PI 502410-3	19F-20		x
PI 502410-4	19F-21		x
PI 502410-5	19F-22		x
PI 502410-6	19F-23		x
PI 502410-7	19F-24		x
PI 502410-8	19F-25		x
PI 502410-9	19F-26		x
PI 502410-10	19F-27		x
PI 502410-11	19F-28		x
PI 502410-12	19F-29		x
PI 502410-14	19F-31		x
PI 502410-15	19F-32		x
PI 502410-18	19F-33		x
PI 502410-19	19F-34		x
PI 502410-20	19F-35		x
PI 650293-1	19F-36	Szarsomlyo hill above Nagyharsany, in limestone grassland. Sedo-Festucetum dalmatica, Villany-Siklos range, Hungary	Interspecific hybrid?
PI 650293-2	19F-37		Interspecific hybrid?
PI 650293-3	19F-38		Interspecific hybrid?
PI 650293-4	19F-39		Interspecific hybrid?

PI 650293-6	19F-41		Interspecific hybrid?
PI 650293-7	19F-42		Interspecific hybrid?
PI 650293-8	19F-43		Interspecific hybrid?
PI 650293-9	19F-44		Interspecific hybrid?
PI 650293-10	19F-45		Interspecific hybrid?
PI 650293-11	19F-46		Interspecific hybrid?
PI 650293-12	19F-47		Interspecific hybrid?
PI 650293-13	19F-48		Interspecific hybrid?
PI 650293-14	19F-49		Interspecific hybrid?
PI 650293-15	19F-50		Interspecific hybrid?
PI 650293-16	19F-51		Interspecific hybrid?
PI 650293-17	19F-52		Interspecific hybrid?
PI 650293-18	19F-53		Interspecific hybrid?
PI 650293-19	19F-54		Interspecific hybrid?
PI 650293-20	19F-55		Interspecific hybrid?
PI 650301-1	19F-96	Near Jil, Gegharkunik Marz, Armenia	x
PI 650301-4	19F-97		x
PI 650301-6	19F-98		x
PI 650301-7	19F-99		x
PI 650301-8	19F-100		x

PI 650301-10	19F-101		x
PI 650301-13	19F-102		x
PI 650301-14	19F-103		x
PI 650301-15	19F-104		x
PI 650301-18	19F-106		x
PI 650301-19	19F-107		x
PI 650301-20	19F-108		x
Ames 29749-1	19F-123	Opuk National Preserve, 20 km southeast of Marfivka, Ukraine	x
Ames 29749-3	19F-125		x
Ames 29749-4	19F-126		x
Ames 29749-5	19F-127		x
Ames 29749-7	19F-128		x
Ames 29749-8	19F-129		x
Ames 29749-9	19F-130		x
Ames 29749-10	19F-131		x
Ames 29749-11	19F-132		x
Ames 29749-12	19F-133		x
Ames 29749-14	19F-134		x
Ames 29749-15	19F-135		x
Ames 29749-16	19F-136		x
Ames 29749-17	19F-137		x
Ames 29749-19	19F-138		x
PI 440473-1	19F-139	Beshpagin Sands, 44km southeast of Stavropol, Russian Federation	x
PI 440473-2	19F-140		x
PI 440473-3	19F-141		x

PI 440473-6	19F-142		x
PI 440473-7	19F-143		x
PI 440473-9	19F-144		x
PI 440473-10	19F-145		x
PI 440473-11	19F-146		x
PI 440473-13	19F-147		x
PI 440473-14	19F-148		x
PI 440473-16	19F-149		x
PI 440473-17	19F-150		x
PI 440473-18	19F-151		x
PI 440473-19	19F-152		x
PI 440473-20	19F-153		x
PI 650294-1	19F-160	Yankuli, Stavropol hills, central Caucasus, Russian Federation	x
PI 650294-2	19F-161		x
PI 650294-3	19F-162		x
PI 650294-4	19F-163		x
PI 650294-5	19F-164		Interspecific hybrid?
PI 650294-6	19F-165		x
PI 650294-7	19F-166		x
PI 650294-8	19F-167		x
PI 650294-9	19F-168		x
PI 650294-10	19F-169		x
PI 650294-12	19F-170		x
PI 650294-13	19F-171		x
PI 650294-14	19F-172		x
PI 650294-15	19F-173		x
PI 650294-16	19F-174		x
PI 650294-17	19F-175		x

PI 650294-18	19F-176		x
PI 650294-19	19F-177		x
PI 650294-20	19F-178		x
PI 650299-1	19F-179	Along roadside near Kaliakra, close to the Black Sea, Dobrich, Bulgaria	x
PI 650299-4	19F-180		x
PI 650299-6	19F-181		x
PI 650299-8	19F-182		x
PI 650299-10	19F-183		x
PI 650299-12	19F-184		x
PI 650299-14	19F-185		x
PI 650299-15	19F-186		x
PI 650299-16	19F-187		x
PI 650299-17	19F-188		x
PI 650299-18	19F-189		x
PI 650299-19	19F-190		x
PI 650300-2	19F-191	Near Tatatszentgyorgy and Orgovany,, the Great Hungarian Plain between Duna (Danube) and Tisza Rivers, Kiskunsag, Bács-Kiskun, Hungary	x
PI 650300-4	19F-192		x
PI 650300-5	19F-193		x
PI 650300-6	19F-194		x
PI 650300-8	19F-195		x

PI 650300-9	19F-196		x
PI 650300-11	19F-197		x
PI 650300-12	19F-198		x
PI 650300-13	19F-199		x
PI 650300-14	19F-200		x
PI 650300-15	19F-201		x
PI 650300-16	19F-202		x
PI 650300-17	19F-203		x
PI 650300-18	19F-204		x
PI 650300-19	19F-205		x
PI 650300-20	19F-206		x
PI 650295-2	19F-207	Collected from Arboretum, but wild origin is Pogorze Przemyskie, Poland	Interspecific hybrid?
PI 650295-4	19F-208		Interspecific hybrid?
PI 650295-6	19F-209		Interspecific hybrid?
PI 650295-7	19F-210		Interspecific hybrid?
PI 650295-8	19F-211		Interspecific hybrid?
PI 650295-9	19F-212		Interspecific hybrid?
PI 650295-10	19F-213		Interspecific hybrid?
PI 650295-11	19F-214		Interspecific hybrid?
PI 650295-12	19F-215		Interspecific hybrid?

PI 650295-13	19F-216		Interspecific hybrid?
PI 650295-14	19F-217		Interspecific hybrid?
PI 650295-15	19F-218		Interspecific hybrid?
PI 650295-16	19F-219		Interspecific hybrid?
PI 650295-17	19F-220		Interspecific hybrid?
PI 650295-18	19F-221		Interspecific hybrid?
PI 650295-19	19F-222		Interspecific hybrid?
PI 650295-20	19F-223		Interspecific hybrid?
PI 650296-1	19F-224	above Albena, Dobrich, Bulgaria	outlier interspecific hybrid
PI 650296-2	19F-225		x
PI 650296-3	19F-226		x
PI 650296-5	19F-228		x
PI 650296-6	19F-229		x
PI 650296-7	19F-230		x
PI 650296-9	19F-231		x
PI 650296-10	19F-232		x
PI 650296-11	19F-233		x
PI 650296-12	19F-234		x
PI 650296-13	19F-235		x
PI 650296-14	19F-236		x
PI 650296-15	19F-237		x
PI 650296-16	19F-238		x
PI 650296-17	19F-239		x

PI 650296-18	19F-240		x
PI 650297-1	19F-241	SW of Galgenberges, field trail Gatersleben-Cochstedt, Germany	Interspecific hybrid?
PI 650297-2	19F-242		Interspecific hybrid?
PI 650297-3	19F-243		Interspecific hybrid?
PI 650297-4	19F-244		Interspecific hybrid?
PI 650297-5	19F-245		Outlier interspecific hybrid
PI 650297-6	19F-246		Interspecific hybrid?
PI 650297-7	19F-247		Interspecific hybrid?
PI 650297-8	19F-248		Interspecific hybrid?
PI 650297-9	19F-249		Interspecific hybrid?
PI 650297-10	19F-250		Interspecific hybrid?
PI 650297-11 rep1	19F-251		Interspecific hybrid?
PI 650297-11 rep 2	19F-252		Interspecific hybrid?
PI 650297-13	19F-253		Interspecific hybrid?

PI 650297-14	19F-254		Interspecific hybrid?
PI 650297-15	19F-255		Interspecific hybrid?
PI 650297-16	19F-256		Interspecific hybrid?
PI 650297-17	19F-257		Interspecific hybrid?
PI 650297-18	19F-258		Interspecific hybrid?
PI 650297-19	19F-259		Interspecific hybrid?
PI 650297-20	19F-260		Interspecific hybrid?
PI 650298-1	19F-771	Rocky hillside with a gentle slope, S-SW exposure. Alkalai soils (pH 9.5), Plovdiv, Bulgaria	x
PI 650298-6	19F-772		x
PI 650298-7	19F-773		x
PI 650298-8	19F-774		x
PI 650298-9	19F-775		x
PI 650298-10	19F-776		x
PI 650298-11	19F-777		x
PI 650298-12	19F-778		x
PI 650298-13	19F-779		x
PI 650298-14	19F-780		x
PI 650298-15	19F-781		x
PI 650298-16	19F-782		x
PI 650298-17	19F-783		x
PI 650298-18	19F-784		x

PI 650298-19	19F-785		x
PI 650298-20	19F-786		x
PI 650298-21	19F-787		x
PI 650298-22	19F-788		x
CN 107255-1	19F-1018	Wild material, no passport data	Interspecific hybrid?
CN 107255-2	19F-1019		Interspecific hybrid?
CN 107255-3	19F-1020		Interspecific hybrid?
CN 107255-4	19F-1021		Interspecific hybrid?
CN 107255-5	19F-1022		Interspecific hybrid?
CN 107255-6	19F-1023		Interspecific hybrid?
CN 107255-7	19F-1024		Interspecific hybrid?
CN 107255-8	19F-1025		Interspecific hybrid?
CN 107255-9	19F-1026		Interspecific hybrid?
CN 107255-10	19F-1027		Interspecific hybrid?
CN 107255-11	19F-1028		Interspecific hybrid?
CN 107255-12	19F-1029		Interspecific hybrid?
CN 107255-13	19F-1030		Interspecific hybrid?
CN 107255-14	19F-1031		Interspecific hybrid?

CN 107255-15	19F-1032		Interspecific hybrid?
CN 107255-17	19F-1033		Interspecific hybrid?
CN 107255-18	19F-1034		Interspecific hybrid?
CN 107255-19	19F-1035		Interspecific hybrid?
CN 107255-20	19F-1036		Interspecific hybrid?
CN 107288-1 Rep 1	19F-1661	Wild material, no passport data	Interspecific hybrid?
CN 107288-2 Rep 1	19F-1662		Interspecific hybrid?
CN 107288-3 Rep 1	19F-1663		Interspecific hybrid?
CN 107288-5 Rep 1	19F-1664		Interspecific hybrid?
CN 107288-6 Rep 1	19F-1665		x
CN 107288-8	19F-1666		x
CN 107288-9	19F-1667		x
CN 107288-10	19F-1668		x
CN 107288-11	19F-1669		x
CN 107288-12	19F-1670		x
CN 107288-13	19F-1671		x
CN 107288-14	19F-1672		x
PI 440472-1	19F-1707	Division 5, Sheep & Goat Farm, Ravine 4, 52km SE of Stavropol, Russian Federation	x
PI 440472-5	19F-1708		x

PI 440472-6	19F-1709		x
PI 440472-7	19F-1710		x
PI 440472-9	19F-1711		x
PI 440472-10	19F-1712		x
PI 440472-11	19F-1713		x
PI 440472-12	19F-1714		x
PI 440472-13	19F-1715		x
PI 440472-14	19F-1716		x
PI 440472-16	19F-1717		x
PI 440472-17	19F-1718		x
CN 107288-1 Rep 2	19F-1769	Wild material, no passport data	Interspecific hybrid?
CN 107288-2 Rep 2	19F-1770		Interspecific hybrid?
CN 107288-3 Rep 2	19F-1771		Interspecific hybrid?
CN 107288-4	19F-1772		Interspecific hybrid?
CN 107288-5 Rep 2	19F-1773		Interspecific hybrid?
CN 107288-6 Rep 2	19F-1774		Interspecific hybrid?
CN 107268-1	19F-1775		Interspecific hybrid?
CN 107268-2	19F-1776		Interspecific hybrid?
CN 107268-3	19F-1777		Interspecific hybrid?

Linum lewisii			
**USDA GRIN Accession (PI or Ames; GRIN-Global, 2022) or Plant Gene Resources of Canada Genetic Resource Information Network (CN) codes - Plant #**	**Identification code for analyses**	**Passport Data (collection location)**	**SNP confirmation for species designation (x) or interspecific hybrid**
Ames 27614-1 ('Maple Grove')	19F-56	Utah, USA	x
Ames 27614-2	19F-57		x
Ames 27614-3	19F-58		x
Ames 27614-4	19F-59		x
Ames 27614-5	19F-60		x
Ames 27614-6	19F-61		x
Ames 27614-7	19F-62		x
Ames 27614-8	19F-63		x
Ames 27614-10	19F-64		x
Ames 27614-12	19F-66		x
Ames 27614-13	19F-67		x
Ames 27614-14	19F-68		x
Ames 27614-15	19F-69		x
Ames 27614-16	19F-70		x
Ames 27614-18	19F-71		x
Ames 27614-20	19F-72		x
Ames 31360-1	19F-73	Arizona, USA	x
Ames 31360-4	19F-75		x
Ames 31360-5	19F-76		x
Ames 31360-6	19F-77		x
Ames 31360-8	19F-78		x

Ames 31360-11	19F-79		x
Ames 31360-15	19F-80		x
Ames 31360-16	19F-81		x
Ames 31360-18	19F-82		x
Ames 31361-7	19F-118	Oregon, USA	x
Ames 31361-8	19F-119		x
Ames 31361-12	19F-120		x
Ames 31361-13	19F-121		x
Ames 31361-14	19F-122		x
PI 522306-1	19F-261	Origin uncertain, N. America	Interspecific hybrid?
PI 522306-2	19F-262		Interspecific hybrid?
PI 522306-3	19F-263		Interspecific hybrid?
PI 522306-4	19F-264		Interspecific hybrid?
PI 522306-6	19F-266		Interspecific hybrid?
PI 522306-7	19F-267		Interspecific hybrid?
PI 522306-8	19F-268		Interspecific hybrid?
PI 522306-9	19F-269		Interspecific hybrid?
PI 522306-10	19F-270		Interspecific hybrid?
PI 522306-11	19F-271		Interspecific hybrid?
PI 522306-12	19F-272		Interspecific hybrid?
PI 522306-13	19F-273		Interspecific hybrid?

PI 522306-14	19F-274		Interspecific hybrid?
PI 522306-15	19F-275		Interspecific hybrid?
PI 522306-17	19F-276		Interspecific hybrid?
PI 522306-19	19F-277		Interspecific hybrid?
PI 522306-20	19F-278		outlier interspecific hybrid
PI 650320-1	19F-279	Utah, USA	Interspecific hybrid?
PI 650320-2	19F-280		Interspecific hybrid?
PI 650320-3	19F-281		Interspecific hybrid?
PI 650320-4	19F-282		Interspecific hybrid?
PI 650320-5	19F-283		Interspecific hybrid?
PI 650320-6	19F-284		x
PI 650320-8	19F-285		Interspecific hybrid?
PI 650320-10	19F-286		Interspecific hybrid?
PI 650320-11	19F-287		Interspecific hybrid?
PI 650320-12	19F-288		Interspecific hybrid?
PI 650320-13	19F-289		Interspecific hybrid?
PI 650320-14	19F-290		Interspecific hybrid?
PI 650320-15	19F-291		Interspecific hybrid?
PI 650320-16	19F-292		Interspecific hybrid?
PI 650320-17	19F-293		Interspecific hybrid?

PI 650320-19	19F-294		Interspecific hybrid?
PI 650320-20	19F-295		Interspecific hybrid?
Ames 29912-1	19F-296	Oregon, USA	x
Ames 29912-2	19F-297		x
Ames 29912-3	19F-298		x
Ames 29912-5	19F-299		x
Ames 29912-6	19F-300		x
Ames 29912-7	19F-301		x
Ames 29912-10	19F-302		x
Ames 29912-11	19F-303		x
Ames 29912-12	19F-304		x
Ames 29912-14	19F-305		x
Ames 29912-16	19F-306		x
Ames 29912-20	19F-308		x
Ames 32568-6	19F-309	Wyoming, USA	x
Ames 32568-8	19F-310		x
Ames 32568-9	19F-311		x
Ames 32568-15	19F-312		x
Ames 33352-1	19F-313	Nevada, USA	x
Ames 33352-3	19F-314		x
Ames 33352-6	19F-315		x
Ames 33352-7	19F-316		x
Ames 33352-10	19F-317		x
Ames 33352-13	19F-318		x
Ames 33352-14	19F-319		x
Ames 33352-15	19F-320		x
Ames 33352-18	19F-321		x
Ames 33352-20	19F-322		x
Ames 33353-1	19F-323	Arizona, USA	x
Ames 33353-2	19F-324		x

Ames 33353-4	19F-325		x
Ames 33353-6	19F-326		x
Ames 33353-8	19F-327		x
Ames 33353-9	19F-328		x
Ames 33353-10	19F-329		x
Ames 33353-11	19F-330		x
Ames 33353-12	19F-331		x
Ames 33353-13	19F-332		x
Ames 33353-15	19F-333		x
Ames 33353-16	19F-334		x
Ames 33353-17	19F-335		x
Ames 33353-20	19F-336		x
Ames 31366-7	19F-394	Arizona, USA	x
Ames 31366-9	19F-395		x
Ames 31366-10	19F-396		x
Ames 31366-13	19F-397		x
Ames 31366-15	19F-398		x
Ames 31366-16	19F-399		x
Ames 31366-17	19F-400		x
Ames 31369-2	19F-401	Nevada, USA	x
Ames 31369-4	19F-402		x
Ames 31369-8	19F-403		x
Ames 31369-9	19F-404		x
Ames 31369-12	19F-405		x
Ames 31369-15	19F-406		x
Ames 31361-1	19F-407	Oregon, USA	x
Ames 31361-3	19F-408		x
Ames 31361-4	19F-409		x
Ames 31361-9	19F-410		x
Ames 31361-10	19F-411		x
Ames 31361-12	19F-412		x

Ames 31361-14	19F-413		x
Ames 31361-15	19F-414		x
Ames 31361-16	19F-415		x
Ames 31361-17	19F-416		x
Ames 31361-18	19F-417		x
Ames 31361-20	19F-418		x
Ames 31365-1	19F-451	Colorado, USA	x
Ames 31365-2	19F-452		x
Ames 31365-5	19F-453		x
Ames 31365-8	19F-454		x
Ames 31365-10	19F-455		x
Ames 31365-11	19F-456		x
Ames 31365-14	19F-457		x
Ames 31365-17	19F-458		x
Ames 31365-18	19F-459		x
Ames 31370-1	19F-460	unknown; no passport data	x
Ames 31370-2	19F-461		x
Ames 31370-6	19F-462		x
Ames 31370-11	19F-463		x
Ames 31370-13	19F-465		x
Ames 31370-14	19F-466		x
Ames 31370-16	19F-467		x
Ames 31370-17	19F-468		x
Ames 31370-19	19F-469		x
Ames 32565-1 rep1	19F-470	Wyoming, USA	Interspecific hybrid?
Ames 32565-3 rep1	19F-471		Interspecific hybrid?
Ames 32565-4 rep1	19F-472		Interspecific hybrid?
Ames 32565-5 rep1	19F-473		Interspecific hybrid?

Ames 32565-6 rep1	19F-474		Interspecific hybrid?
Ames 32565-7 rep1	19F-475		Interspecific hybrid?
Ames 32565-8 rep1	19F-476		Interspecific hybrid?
Ames 32565-9 rep1	19F-477		Interspecific hybrid?
Ames 32565-10 rep1	19F-478		Interspecific hybrid?
Ames 32565-11 rep1	19F-479		Interspecific hybrid?
Ames 32565-12 rep1	19F-480		Interspecific hybrid?
Ames 32565-13 rep1	19F-481		Interspecific hybrid?
Ames 32565-14 rep1	19F-482		Interspecific hybrid?
Ames 32565-15 rep1	19F-483		Interspecific hybrid?
Ames 32565-16 rep1	19F-484		Interspecific hybrid?
Ames 32565-17 rep1	19F-485		Interspecific hybrid?
Ames 32565-18 rep1	19F-486		Interspecific hybrid?
Ames 32565-19 rep1	19F-487		Interspecific hybrid?
Ames 32565-20 rep 1	19F-488		Interspecific hybrid?
Ames 33354-1	19F-553	Nevada, USA	x
Ames 33354-2	19F-554		x
Ames 33354-6	19F-555		x
Ames 33354-9	19F-556		x
Ames 33354-10	19F-557		x
Ames 33354-14	19F-558		x
Ames 33354-17	19F-559		x
Ames 33354-19	19F-560		x

PI 522305-1	19F-561	origin is unknown	Interspecific hybrid?
PI 522305-2	19F-562		Interspecific hybrid?
PI 522305-3	19F-563		Interspecific hybrid?
PI 522305-4	19F-564		Interspecific hybrid?
PI 522305-6	19F-565		Interspecific hybrid?
PI 522305-8	19F-566		Interspecific hybrid?
PI 522305-9	19F-567		Interspecific hybrid?
PI 522305-12	19F-568		Interspecific hybrid?
PI 522305-13	19F-569		Interspecific hybrid?
PI 522305-15	19F-570		Interspecific hybrid?
PI 522305-16	19F-571		Interspecific hybrid?
PI 522305-17	19F-572		Interspecific hybrid?
PI 452487-1	19F-573	Montana, USA	x
PI 452487-2	19F-574		x
PI 452487-4	19F-575		x
PI 452487-5	19F-576		x
PI 452487-6	19F-577		x
PI 452487-7	19F-578		x
PI 452487-8	19F-579		x
PI 452487-9	19F-580		x
PI 452487-10	19F-581		x
PI 452487-11	19F-582		x
PI 452487-13	19F-583		x
PI 452487-17	19F-584		x

Ames 31368-1	19F-722	Nevada, USA	x
Ames 31368-2	19F-723		x
Ames 31368-4	19F-724		x
Ames 31368-5	19F-725		x
Ames 31368-6	19F-726		x
Ames 31368-7	19F-727		x
Ames 31368-10	19F-728		x
Ames 31368-13	19F-729		x
Ames 31368-14	19F-730		x
Ames 31368-16	19F-731		x
Ames 31368-17	19F-732		x
Ames 31368-19	19F-733		x
Ames 31364-3	19F-904	Colorado, USA	x
Ames 31364-4	19F-905		x
Ames 31364-5	19F-906		x
Ames 31364-7	19F-907		x
Ames 31364-8	19F-908		x
Ames 31364-11	19F-909		x
Ames 31364-15	19F-910		x
Ames 31364-16	19F-911		x
CN 107266-1	19F-1061	unknown	Interspecific hybrid?
CN 107266-2	19F-1062		Interspecific hybrid?
CN 107266-3	19F-1063		Interspecific hybrid?
CN 107266-4	19F-1064		Interspecific hybrid?
CN 107266-5	19F-1065		Interspecific hybrid?
CN 107266-6	19F-1066		Interspecific hybrid?
CN 107266-7	19F-1067		Interspecific hybrid?

CN 107266-8	19F-1068		Interspecific hybrid?
CN 107266-9	19F-1069		Interspecific hybrid?
CN 107266-10	19F-1070		Interspecific hybrid?
CN 107266-11	19F-1071		Interspecific hybrid?
CN 107266-12	19F-1072		Interspecific hybrid?
Ames 31371-1	19F-1563	unknown; no passport data	x
Ames 31371-2	19F-1564		x
Ames 31371-4	19F-1565		x
Ames 31371-6	19F-1566		x
Ames 31371-8	19F-1567		x
Ames 31371-9	19F-1568		x
Ames 31371-10	19F-1569		x
Ames 31371-14	19F-1570		x
Ames 31371-15	19F-1571		x
Ames 31372-1	19F-1572	Wyoming, USA	x
Ames 31372-7	19F-1573		x
Ames 31372-10	19F-1574		x
Ames 31372-11	19F-1575		x
Ames 32565-1 rep2	19F-1576	Wyoming, USA	Interspecific hybrid?
Ames 32565-3 rep2	19F-1577		outlier interspecific hybrid
Ames 32565-4 rep2	19F-1578		Interspecific hybrid?
Ames 32565-5 rep2	19F-1579		Interspecific hybrid?
Ames 32565-6 rep2	19F-1580		Interspecific hybrid?
Ames 32565-7 rep2	19F-1581		Interspecific hybrid?

Ames 32565-8 rep2	19F-1582		Interspecific hybrid?
Ames 32565-9 rep2	19F-1583		Interspecific hybrid?
Ames 32565-10 rep2	19F-1584		Interspecific hybrid?
Ames 32565-11 rep2	19F-1585		Interspecific hybrid?
Ames 32565-12 rep2	19F-1586		Interspecific hybrid?
Ames 32565-13 rep2	19F-1587		Interspecific hybrid?
Ames 32565-14 rep2	19F-1588		Interspecific hybrid?
Ames 32565-15 rep2	19F-1589		Interspecific hybrid?
Ames 32565-16 rep2	19F-1590		Interspecific hybrid?
Ames 32565-17 rep2	19F-1591		Interspecific hybrid?
Ames 32565-18 rep2	19F-1592		Interspecific hybrid?
Ames 32565-19 rep2	19F-1593		Interspecific hybrid?
Ames 32565-20 rep2	19F-1594		Interspecific hybrid?
Ames 32566-1	19F-1595	Utah, USA	x
Ames 32566-2	19F-1596		x
Ames 32566-3	19F-1597		x
Ames 32566-4	19F-1598		x
Ames 32566-6	19F-1599		x
Ames 32566-7	19F-1600		x
Ames 32566-8	19F-1601		x
Ames 32566-9	19F-1602		x
Ames 32566-10	19F-1603		x
Ames 32566-11	19F-1604		x
Ames 32566-14	19F-1605		x

Ames 32566-16	19F-1606		x
CN 107300-2	19F-1625	unknown	x
CN 107300-4	19F-1626		x

Linum perenne			
**USDA GRIN Accession (PI or Ames; GRIN-Global, 2022), Alaskan Wildflowers (AK) or Plant Gene Resources of Canada Genetic Resource Information Network (CN) codes - Plant #**	**Identification code for analyses**	**Passport Data (collection location)**	**SNP confirmation for species designation (x) or interspecific hybrid**
PI 650327-1	19F-337	near Vorosbereny, Lake Balaton area, N coast, Hungary	Interspecific hybrid?
PI 650327-2	19F-338		Interspecific hybrid?
PI 650327-3	19F-339		Interspecific hybrid?
PI 650327-4	19F-340		Interspecific hybrid?
PI 650327-6	19F-341		Interspecific hybrid?
PI 650327-7	19F-342		Interspecific hybrid?
PI 650327-8	19F-343		Interspecific hybrid?
PI 650327-9	19F-344		Interspecific hybrid?
PI 650327-10	19F-345		Interspecific hybrid?
PI 650327-11	19F-346		Interspecific hybrid?
PI 650327-12	19F-347		Interspecific hybrid?
PI 650327-13	19F-348		Interspecific hybrid?

PI 650327-14	19F-349		Interspecific hybrid?
PI 650327-15	19F-350		Interspecific hybrid?
PI 650327-16	19F-351		Interspecific hybrid?
PI 650327-17	19F-352		Interspecific hybrid?
PI 650327-18	19F-353		Interspecific hybrid?
PI 650327-19	19F-354		Interspecific hybrid?
PI 650327-20	19F-355		Interspecific hybrid?
PI 650328-1	19F-356	Sawtooth National Recreation Area, Idaho, USA (naturalized)	Interspecific hybrid?
PI 650328-2	19F-357		Interspecific hybrid?
PI 650328-3	19F-358		Interspecific hybrid?
PI 650328-4	19F-359		Interspecific hybrid?
PI 650328-6	19F-360		Interspecific hybrid?
PI 650328-7	19F-361		Interspecific hybrid?
PI 650328-8	19F-362		Interspecific hybrid?
PI 650328-9	19F-363		Interspecific hybrid?
PI 650328-10	19F-364		Interspecific hybrid?
PI 650328-11	19F-365		Interspecific hybrid?
PI 650328-12	19F-366		Interspecific hybrid?
PI 650328-13	19F-367		Interspecific hybrid?

PI 650328-14	19F-368		Interspecific hybrid?
PI 650328-15	19F-369		Interspecific hybrid?
PI 650328-16	19F-370		Interspecific hybrid?
PI 650328-17	19F-371		Interspecific hybrid?
PI 650328-18	19F-372		Interspecific hybrid?
PI 650328-19	19F-373		Interspecific hybrid?
PI 650328-20	19F-374		Interspecific hybrid?
PI 650329-1	19F-375	Bureau of Land Management building, Boise, Idaho, USA (naturalized)	Interspecific hybrid?
PI 650329-2	19F-376		Interspecific hybrid?
PI 650329-3	19F-377		Interspecific hybrid?
PI 650329-4	19F-378		Interspecific hybrid?
PI 650329-5	19F-379		Interspecific hybrid?
PI 650329-6	19F-380		Interspecific hybrid?
PI 650329-7	19F-381		Interspecific hybrid?
PI 650329-8	19F-382		Interspecific hybrid?
PI 650329-9	19F-383		Interspecific hybrid?
PI 650329-10	19F-384		Interspecific hybrid?
PI 650329-11	19F-385		Interspecific hybrid?

PI 650329-12	19F-386		Interspecific hybrid?
PI 650329-13	19F-387		Interspecific hybrid?
PI 650329-14	19F-388		Interspecific hybrid?
PI 650329-15	19F-389		Interspecific hybrid?
PI 650329-16	19F-390		Interspecific hybrid?
PI 650329-18	19F-391		Interspecific hybrid?
PI 650329-19	19F-392		Interspecific hybrid?
PI 650329-20	19F-393		Interspecific hybrid?
Ames 21222-1	19F-499	Moldova	Interspecific hybrid?
Ames 21222-2	19F-500		Interspecific hybrid?
Ames 21222-3	19F-501		Interspecific hybrid?
Ames 21222-4	19F-502		Interspecific hybrid?
Ames 21222-5	19F-503		Interspecific hybrid?
Ames 21222-6	19F-504		Interspecific hybrid?
Ames 21222-8	19F-505		Interspecific hybrid?
Ames 21222-12	19F-506		Interspecific hybrid?
Ames 21222-13	19F-507		Interspecific hybrid?
Ames 21222-14	19F-508		Interspecific hybrid?
Ames 21222-15	19F-509		Interspecific hybrid?
Ames 21222-16	19F-510		Interspecific hybrid?

Ames 21222-17	19F-511		Interspecific hybrid?
Ames 21222-18	19F-512		Interspecific hybrid?
Ames 21222-19	19F-513		Interspecific hybrid?
Ames 21222-20	19F-514		Interspecific hybrid?
Ames 31374-1	19F-515	Oregon, USA (naturalized)	Interspecific hybrid?
Ames 31374-2	19F-516		Interspecific hybrid?
Ames 31374-3	19F-517		Interspecific hybrid?
Ames 31374-4	19F-518		Interspecific hybrid?
Ames 31374-5	19F-519		Interspecific hybrid?
Ames 31374-6	19F-520		Interspecific hybrid?
Ames 31374-7	19F-521		Interspecific hybrid?
Ames 31374-8	19F-522		Interspecific hybrid?
Ames 31374-9	19F-523		Interspecific hybrid?
Ames 31374-10	19F-524		Interspecific hybrid?
Ames 31374-11	19F-525		Interspecific hybrid?
Ames 31374-12	19F-526		Interspecific hybrid?
Ames 31374-13	19F-527		Interspecific hybrid?
Ames 31374-14	19F-528		Interspecific hybrid?
Ames 31374-15	19F-529		Interspecific hybrid?
Ames 31374-16	19F-530		Interspecific hybrid?

Ames 31374-18	19F-532		Interspecific hybrid?
Ames 31374-19	19F-533		Interspecific hybrid?
Ames 31374-20	19F-534		Interspecific hybrid?
PI 650324-1	19F-535	Hungary	x
PI 650324-2	19F-536		x
PI 650324-3	19F-537		x
PI 650324-4	19F-538		x
PI 650324-5	19F-539		x
PI 650324-6	19F-540		x
PI 650324-7	19F-541		x
PI 650324-8	19F-542		x
PI 650324-9	19F-543		x
PI 650324-10	19F-544		x
PI 650324-11	19F-545		x
PI 650324-13	19F-546		x
PI 650324-14	19F-547		x
PI 650324-15	19F-548		x
PI 650324-16	19F-549		x
PI 650324-17	19F-550		x
PI 650324-18	19F-551		x
PI 650324-19	19F-552		x
PI 650325-1	19F-734	Orgovany, Hungary	x
PI 650325-2	19F-735		x
PI 650325-3	19F-736		x
PI 650325-4	19F-737		x
PI 650325-5	19F-738		x
PI 650325-6	19F-739		x
PI 650325-7	19F-740		x
PI 650325-8	19F-741		x

PI 650325-9	19F-742		x
PI 650325-10	19F-743		x
PI 650325-11	19F-744		x
PI 650325-12	19F-745		x
PI 650325-13	19F-746		x
PI 650325-14	19F-747		x
PI 650325-15	19F-748		x
PI 650325-16	19F-749		x
PI 650325-17	19F-750		x
PI 650325-18	19F-751		x
PI 650325-19	19F-752		x
PI 650325-20	19F-753		x
PI 650326-1	19F-754	Lake Balaton area, N coast, Hungary	Interspecific hybrid?
PI 650326-2	19F-755		Interspecific hybrid?
PI 650326-3	19F-756		Interspecific hybrid?
PI 650326-5	19F-757		Interspecific hybrid?
PI 650326-6	19F-758		Interspecific hybrid?
PI 650326-7	19F-759		Interspecific hybrid?
PI 650326-8	19F-760		Interspecific hybrid?
PI 650326-10	19F-761		Interspecific hybrid?
PI 650326-12	19F-762		Interspecific hybrid?
PI 650326-13	19F-763		Interspecific hybrid?
PI 650326-14	19F-764		Interspecific hybrid?
PI 650326-15	19F-765		Interspecific hybrid?

PI 650326-16	19F-766		Interspecific hybrid?
PI 650326-18	19F-768		Interspecific hybrid?
PI 650326-19	19F-769		Interspecific hybrid?
PI 650326-20	19F-770		Interspecific hybrid?
Blue Flax-1 Rep 1	19F-932	American Meadows Seed Co., USA (origin unknown)	Interspecific hybrid?
Blue Flax-2	19F-933		Interspecific hybrid?
Blue Flax-3	19F-934		Interspecific hybrid?
Blue Flax-4	19F-935		Interspecific hybrid?
Blue Flax-5	19F-936		Interspecific hybrid?
Blue Flax-6	19F-937		Interspecific hybrid?
Blue Flax-7	19F-938		Interspecific hybrid?
Blue Flax-8	19F-939		Interspecific hybrid?
Blue Flax-10	19F-940		Interspecific hybrid?
Blue Flax-11	19F-941		Interspecific hybrid?
Blue Flax-12	19F-942		Interspecific hybrid?
Blue Flax-13	19F-943		Interspecific hybrid?
Blue Flax-15	19F-945		Interspecific hybrid?
Blue Flax-16	19F-946		Interspecific hybrid?
Blue Flax-17	19F-947		Interspecific hybrid?

Blue Flax-19	19F-948		Interspecific hybrid?
Blue Flax-20	19F-949		Interspecific hybrid?
CN 107283-1	19F-982	Wild material, Hungary	x
CN 107283-2	19F-983		x
CN 107283-4	19F-984		x
CN 107283-5	19F-985		x
CN 107283-6	19F-986		x
CN 107283-7	19F-987		x
CN 107283-8	19F-988		x
CN 107283-9	19F-989		x
CN 107283-11	19F-991		x
CN 107283-12	19F-992		x
CN 107283-13	19F-993		x
CN 107283-14	19F-994		x
CN 107283-15	19F-995		x
CN 107283-16	19F-996		x
CN 107283-17	19F-997		x
CN 107283-18	19F-998		x
CN 107283-19	19F-999		x
CN 107283-20	19F-1000		x
PI 445972-1	19F-1001	South Dakota, USA (naturalized)	Interspecific hybrid?
PI 445972-2	19F-1002		Interspecific hybrid?
PI 445972-3	19F-1003		Interspecific hybrid?
PI 445972-4	19F-1004		Interspecific hybrid?
PI 445972-5	19F-1005		Interspecific hybrid?
PI 445972-6	19F-1006		Interspecific hybrid?

PI 445972-7	19F-1007		Interspecific hybrid?
PI 445972-8	19F-1008		Interspecific hybrid?
PI 445972-9	19F-1009		Interspecific hybrid?
PI 445972-10	19F-1010		Interspecific hybrid?
PI 445972-11	19F-1011		Interspecific hybrid?
PI 445972-12	19F-1012		Interspecific hybrid?
PI 445972-13	19F-1013		Interspecific hybrid?
PI 445972-14	19F-1014		Interspecific hybrid?
PI 445972-15	19F-1015		Interspecific hybrid?
PI 445972-16	19F-1016		Interspecific hybrid?
PI 445972-17	19F-1017		Interspecific hybrid?
Blue Flax-1 Rep 2	19F-1039	American Meadows Seed Co., USA (origin unknown)	Interspecific hybrid?
AK wildflowers-2	19F-1040	Alaska, USA (naturalized), (origin unknown)	Interspecific hybrid?
AK wildflowers-3	19F-1041		Interspecific hybrid?
AK wildflowers-4	19F-1042		Interspecific hybrid?
AK wildflowers-5	19F-1043		Interspecific hybrid?
AK wildflowers-6	19F-1044		Interspecific hybrid?
AK wildflowers-7	19F-1045		Interspecific hybrid?
AK wildflowers-8	19F-1046		Interspecific hybrid?

AK wildflowers-9	19F-1047		Interspecific hybrid?
AK wildflowers-10	19F-1048		Interspecific hybrid?
AK wildflowers-11	19F-1049		Interspecific hybrid?
AK wildflowers-12	19F-1050		Interspecific hybrid?
AK wildflowers-13	19F-1051		Interspecific hybrid?
AK wildflowers-14	19F-1052		Interspecific hybrid?
AK wildflowers-15	19F-1053		Interspecific hybrid?
PI 445972-2	19F-1094	South Dakota, USA (naturalized)	Interspecific hybrid?
PI 445972-3	19F-1095		Interspecific hybrid?
PI 445972-4	19F-1096		Interspecific hybrid?
PI 445972-5	19F-1097		Interspecific hybrid?
PI 445972-6	19F-1098		Interspecific hybrid?
PI 445972-7	19F-1099		Interspecific hybrid?
PI 445972-8	19F-1100		Interspecific hybrid?
PI 445972-9	19F-1101		Interspecific hybrid?
PI 445972-10	19F-1102		Interspecific hybrid?
PI 445972-11	19F-1103		Interspecific hybrid?
PI 445972-12	19F-1104		Interspecific hybrid?
PI 445972-13	19F-1105		Interspecific hybrid?
PI 445972-14	19F-1106		Interspecific hybrid?

PI 445972-15	19F-1107		Interspecific hybrid?
PI 445972-16	19F-1108		Interspecific hybrid?
PI 445972-18	19F-1109		Interspecific hybrid?
PI 445972-20	19F-1110		Interspecific hybrid?
PI 650323-1	19F-1518	Hungary	x
PI 650323-2	19F-1519		x
PI 650323-3	19F-1520		x
PI 650323-4	19F-1521		x
PI 650323-5	19F-1522		x
PI 650323-6	19F-1523		x
PI 650323-7	19F-1524		x
PI 650323-8	19F-1525		x
PI 650323-9	19F-1526		x
PI 650323-10	19F-1527		x
PI 650323-11	19F-1528		x
PI 650323-12	19F-1529		x
PI 650323-13	19F-1530		x
PI 650323-14	19F-1531		x
PI 650323-15	19F-1532		x
PI 650323-16	19F-1533		x
PI 650323-17	19F-1534		x
PI 650323-18	19F-1535		x
PI 650323-19	19F-1536		x
PI 650323-20	19F-1537		x
CN 107261-1	19F-1647	wild material, origin unknown	Interspecific hybrid?
CN 107261-4	19F-1649		Interspecific hybrid?
CN 19179-1	19F-1673	wild material, origin unknown	x

CN 19179-2	19F-1674		x
CN 19179-3	19F-1675		x
CN 19179-5	19F-1676		x
CN 19179-8	19F-1677		x
CN 107282-1	19F-1678	wild material, origin unknown	x
CN 107282-2	19F-1679		x
CN 107282-5	19F-1680		x
CN 19024-1, 'Himmelszelt'	19F-1778	origin unknown	Interspecific hybrid?
CN 19024-2	19F-1779		Interspecific hybrid?
CN 19024-3	19F-1780		Interspecific hybrid?
CN 19024-4	19F-1781		Interspecific hybrid?
CN 19024-5	19F-1782		Interspecific hybrid?
CN 19024-6	19F-1783		Interspecific hybrid?
CN 19024-7	19F-1784		Interspecific hybrid?
CN 19024-8	19F-1785		Interspecific hybrid?
CN 19024-9	19F-1786		Interspecific hybrid?
CN 19024-10	19F-1787		Interspecific hybrid?
CN 19024-11	19F-1788		Interspecific hybrid?
CN 19024-12	19F-1789		Interspecific hybrid?
CN 19024-13	19F-1790		Interspecific hybrid?
CN 19024-14	19F-1791		Interspecific hybrid?
CN 107256-1	19F-1792	Wild material, origin unknown	Interspecific hybrid?

CN 107256-2	19F-1793		Interspecific hybrid?
CN 107256-3	19F-1794		Interspecific hybrid?
CN 107256-4	19F-1795		Interspecific hybrid?
CN 107256-5	19F-1796		Interspecific hybrid?
CN 107256-6	19F-1797		Interspecific hybrid?
CN 107256-7	19F-1798		Interspecific hybrid?
CN 107256-8	19F-1799		Interspecific hybrid?
CN 107256-10	19F-1800		Interspecific hybrid?
CN 107256-11	19F-1801		Interspecific hybrid?
CN 107256-12	19F-1802		Interspecific hybrid?
CN 107256-13	19F-1803		Interspecific hybrid?
CN 107256-14	19F-1804		Interspecific hybrid?
CN 107256-15	19F-1805		Interspecific hybrid?
CN 107256-16	19F-1806		Interspecific hybrid?
CN 107256-17	19F-1807		Interspecific hybrid?
CN 107256-18	19F-1808		Interspecific hybrid?
CN 107270-1	19F-1809	Wild material, origin unknown	Interspecific hybrid?
CN 107270-2	19F-1810		Interspecific hybrid?
CN 107270-4	19F-1811		Interspecific hybrid?
CN 107270-5	19F-1812		Interspecific hybrid?

CN 107270-6	19F-1813	Interspecific hybrid?
CN 107270-7	19F-1814	Interspecific hybrid?
CN 107270-8	19F-1815	Interspecific hybrid?
CN 107270-9	19F-1816	Interspecific hybrid?
CN 107270-10	19F-1817	Interspecific hybrid?
CN 107270-11	19F-1818	Interspecific hybrid?
CN 107270-13	19F-1819	Interspecific hybrid?
CN 107270-14	19F-1820	Interspecific hybrid?
CN 107270-15	19F-1821	Interspecific hybrid?
CN 107270-16	19F-1822	Interspecific hybrid?
CN 107270-17	19F-1823	Interspecific hybrid?
CN 107270-18	19F-1824	Interspecific hybrid?
CN 107270-19	19F-1825	Interspecific hybrid?
CN 107270-20	19F-1826	Interspecific hybrid?

Supplemental Table S2. Perennial *Linum* from the “hybrid” cluster with USDA determined species designation along with USDA accession code and identification code for analyses. Yellow highlights denote genotypes sorted for positive Ys for the top pure taxon cluster; all negatives would be in the lower cluster.

Table S2 – Sheet 1, All three species hybrid cluster

USDA determined species	GRIN - Plant number	Identification code
Linum austriacum	CN 107255-1	19F-1018
Linum austriacum	CN 107255-2	19F-1019
Linum austriacum	CN 107255-3	19F-1020
Linum austriacum	CN 107255-4	19F-1021
Linum austriacum	CN 107255-5	19F-1022
Linum austriacum	CN 107255-6	19F-1023
Linum austriacum	CN 107255-7	19F-1024
Linum austriacum	CN 107255-8	19F-1025
Linum austriacum	CN 107255-9	19F-1026
Linum austriacum	CN 107255-10	19F-1027
Linum austriacum	CN 107255-11	19F-1028
Linum austriacum	CN 107255-12	19F-1029
Linum austriacum	CN 107255-13	19F-1030
Linum austriacum	CN 107255-14	19F-1031
Linum austriacum	CN 107255-15	19F-1032
Linum austriacum	CN 107255-17	19F-1033
Linum austriacum	CN 107255-18	19F-1034
Linum austriacum	CN 107255-19	19F-1035
Linum austriacum	CN 107255-20	19F-1036
Linum austriacum	PI 650294-5	19F-164
Linum austriacum	CN 107288-1	19F-1661
Linum austriacum	CN 107288-2	19F-1662
Linum austriacum	CN 107288-3	19F-1663
Linum austriacum	CN 107288-5	19F-1664

Linum austriacum	CN 107288-1	19F-1769
Linum austriacum	CN 107288-2	19F-1770
Linum austriacum	CN 107288-3	19F-1771
Linum austriacum	CN 107288-4	19F-1772
Linum austriacum	CN 107288-5	19F-1773
Linum austriacum	CN 107288-6	19F-1774
Linum austriacum	CN 107268-1	19F-1775
Linum austriacum	CN 107268-2	19F-1776
Linum austriacum	CN 107268-3	19F-1777
Linum austriacum	PI 650295-2	19F-207
Linum austriacum	PI 650295-4	19F-208
Linum austriacum	PI 650295-6	19F-209
Linum austriacum	PI 650295-7	19F-210
Linum austriacum	PI 650295-8	19F-211
Linum austriacum	PI 650295-9	19F-212
Linum austriacum	PI 650295-10	19F-213
Linum austriacum	PI 650295-11	19F-214
Linum austriacum	PI 650295-12	19F-215
Linum austriacum	PI 650295-13	19F-216
Linum austriacum	PI 650295-14	19F-217
Linum austriacum	PI 650295-15	19F-218
Linum austriacum	PI 650295-16	19F-219
Linum austriacum	PI 650295-17	19F-220
Linum austriacum	PI 650295-18	19F-221
Linum austriacum	PI 650295-19	19F-222
Linum austriacum	PI 650295-20	19F-223
Linum austriacum	PI 650296-1	19F-224
Linum austriacum	PI 650297-1	19F-241
Linum austriacum	PI 650297-2	19F-242
Linum austriacum	PI 650297-3	19F-243
Linum austriacum	PI 650297-4	19F-244

Linum austriacum	PI 650297-5	19F-245
Linum austriacum	PI 650297-6	19F-246
Linum austriacum	PI 650297-7	19F-247
Linum austriacum	PI 650297-8	19F-248
Linum austriacum	PI 650297-9	19F-249
Linum austriacum	PI 650297-10	19F-250
Linum austriacum	PI 650297-11	19F-251
Linum austriacum	PI 650297-11	19F-252
Linum austriacum	PI 650297-13	19F-253
Linum austriacum	PI 650297-14	19F-254
Linum austriacum	PI 650297-15	19F-255
Linum austriacum	PI 650297-16	19F-256
Linum austriacum	PI 650297-17	19F-257
Linum austriacum	PI 650297-18	19F-258
Linum austriacum	PI 650297-19	19F-259
Linum austriacum	PI 650297-20	19F-260
Linum austriacum	PI 650293-1	19F-36
Linum austriacum	PI 650293-2	19F-37
Linum austriacum	PI 650293-3	19F-38
Linum austriacum	PI 650293-4	19F-39
Linum austriacum	PI 650293-6	19F-41
Linum austriacum	PI 650293-7	19F-42
Linum austriacum	PI 650293-8	19F-43
Linum austriacum	PI 650293-9	19F-44
Linum austriacum	PI 650293-10	19F-45
Linum austriacum	PI 650293-11	19F-46
Linum austriacum	PI 650293-12	19F-47
Linum austriacum	PI 650293-13	19F-48
Linum austriacum	PI 650293-14	19F-49
Linum austriacum	PI 650293-15	19F-50
Linum austriacum	PI 650293-16	19F-51

Linum austriacum	PI 650293-17	19F-52
Linum austriacum	PI 650293-18	19F-53
Linum austriacum	PI 650293-19	19F-54
Linum austriacum	PI 650293-20	19F-55
Linum lewisii	CN 107266-1	19F-1061
Linum lewisii	CN 107266-2	19F-1062
Linum lewisii	CN 107266-3	19F-1063
Linum lewisii	CN 107266-4	19F-1064
Linum lewisii	CN 107266-5	19F-1065
Linum lewisii	CN 107266-6	19F-1066
Linum lewisii	CN 107266-7	19F-1067
Linum lewisii	CN 107266-8	19F-1068
Linum lewisii	CN 107266-9	19F-1069
Linum lewisii	CN 107266-10	19F-1070
Linum lewisii	CN 107266-11	19F-1071
Linum lewisii	CN 107266-12	19F-1072
Linum lewisii	Ames 32565-1	19F-1576
Linum lewisii	Ames 32565-3	19F-1577
Linum lewisii	Ames 32565-4	19F-1578
Linum lewisii	Ames 32565-5	19F-1579
Linum lewisii	Ames 32565-6	19F-1580
Linum lewisii	Ames 32565-7	19F-1581
Linum lewisii	Ames 32565-8	19F-1582
Linum lewisii	Ames 32565-9	19F-1583
Linum lewisii	Ames 32565-10	19F-1584
Linum lewisii	Ames 32565-11	19F-1585
Linum lewisii	Ames 32565-12	19F-1586
Linum lewisii	Ames 32565-13	19F-1587
Linum lewisii	Ames 32565-14	19F-1588
Linum lewisii	Ames 32565-15	19F-1589
Linum lewisii	Ames 32565-16	19F-1590

Linum lewisii	Ames 32565-17	19F-1591
Linum lewisii	Ames 32565-18	19F-1592
Linum lewisii	Ames 32565-19	19F-1593
Linum lewisii	Ames 32565-20	19F-1594
Linum lewisii	PI 522306-1	19F-261
Linum lewisii	PI 522306-2	19F-262
Linum lewisii	PI 522306-3	19F-263
Linum lewisii	PI 522306-4	19F-264
Linum lewisii	PI 522306-6	19F-266
Linum lewisii	PI 522306-7	19F-267
Linum lewisii	PI 522306-8	19F-268
Linum lewisii	PI 522306-9	19F-269
Linum lewisii	PI 522306-10	19F-270
Linum lewisii	PI 522306-11	19F-271
Linum lewisii	PI 522306-12	19F-272
Linum lewisii	PI 522306-13	19F-273
Linum lewisii	PI 522306-14	19F-274
Linum lewisii	PI 522306-15	19F-275
Linum lewisii	PI 522306-17	19F-276
Linum lewisii	PI 522306-19	19F-277
Linum lewisii	PI 522306-20	19F-278
Linum lewisii	PI 650320-1	19F-279
Linum lewisii	PI 650320-2	19F-280
Linum lewisii	PI 650320-3	19F-281
Linum lewisii	PI 650320-4	19F-282
Linum lewisii	PI 650320-5	19F-283
Linum lewisii	PI 650320-6	19F-284
Linum lewisii	PI 650320-8	19F-285
Linum lewisii	PI 650320-10	19F-286
Linum lewisii	PI 650320-11	19F-287
Linum lewisii	PI 650320-12	19F-288

Linum lewisii	PI 650320-13	19F-289
Linum lewisii	PI 650320-14	19F-290
Linum lewisii	PI 650320-15	19F-291
Linum lewisii	PI 650320-16	19F-292
Linum lewisii	PI 650320-17	19F-293
Linum lewisii	PI 650320-19	19F-294
Linum lewisii	PI 650320-20	19F-295
Linum lewisii	Ames 32565-1	19F-470
Linum lewisii	Ames 32565-3	19F-471
Linum lewisii	Ames 32565-4	19F-472
Linum lewisii	Ames 32565-5	19F-473
Linum lewisii	Ames 32565-6	19F-474
Linum lewisii	Ames 32565-7	19F-475
Linum lewisii	Ames 32565-8	19F-476
Linum lewisii	Ames 32565-9	19F-477
Linum lewisii	Ames 32565-10	19F-478
Linum lewisii	Ames 32565-11	19F-479
Linum lewisii	Ames 32565-12	19F-480
Linum lewisii	Ames 32565-13	19F-481
Linum lewisii	Ames 32565-14	19F-482
Linum lewisii	Ames 32565-15	19F-483
Linum lewisii	Ames 32565-16	19F-484
Linum lewisii	Ames 32565-17	19F-485
Linum lewisii	Ames 32565-18	19F-486
Linum lewisii	Ames 32565-19	19F-487
Linum lewisii	Ames 32565-20	19F-488
Linum lewisii	PI 522305-1	19F-561
Linum lewisii	PI 522305-2	19F-562
Linum lewisii	PI 522305-3	19F-563
Linum lewisii	PI 522305-4	19F-564
Linum lewisii	PI 522305-6	19F-565

Linum lewisii	PI 522305-8	19F-566
Linum lewisii	PI 522305-9	19F-567
Linum lewisii	PI 522305-12	19F-568
Linum lewisii	PI 522305-13	19F-569
Linum lewisii	PI 522305-15	19F-570
Linum lewisii	PI 522305-16	19F-571
Linum lewisii	PI 522305-17	19F-572
Linum perenne	PI 445972-1	19F-1001
Linum perenne	PI 445972-2	19F-1002
Linum perenne	PI 445972-3	19F-1003
Linum perenne	PI 445972-4	19F-1004
Linum perenne	PI 445972-5	19F-1005
Linum perenne	PI 445972-6	19F-1006
Linum perenne	PI 445972-7	19F-1007
Linum perenne	PI 445972-8	19F-1008
Linum perenne	PI 445972-9	19F-1009
Linum perenne	PI 445972-10	19F-1010
Linum perenne	PI 445972-11	19F-1011
Linum perenne	PI 445972-12	19F-1012
Linum perenne	PI 445972-13	19F-1013
Linum perenne	PI 445972-14	19F-1014
Linum perenne	PI 445972-15	19F-1015
Linum perenne	PI 445972-16	19F-1016
Linum perenne	PI 445972-17	19F-1017
Linum perenne	Blue Flax-1	19F-1039
Linum perenne	AK wildflowers-2	19F-1040
Linum perenne	AK wildflowers-3	19F-1041
Linum perenne	AK wildflowers-4	19F-1042
Linum perenne	AK wildflowers-5	19F-1043
Linum perenne	AK wildflowers-6	19F-1044
Linum perenne	AK wildflowers-7	19F-1045

Linum perenne	AK wildflowers-8	19F-1046
Linum perenne	AK wildflowers-9	19F-1047
Linum perenne	AK wildflowers-10	19F-1048
Linum perenne	AK wildflowers-11	19F-1049
Linum perenne	AK wildflowers-12	19F-1050
Linum perenne	AK wildflowers-13	19F-1051
Linum perenne	AK wildflowers-14	19F-1052
Linum perenne	AK wildflowers-15	19F-1053
Linum perenne	PI 445972-2	19F-1094
Linum perenne	PI 445972-3	19F-1095
Linum perenne	PI 445972-4	19F-1096
Linum perenne	PI 445972-5	19F-1097
Linum perenne	PI 445972-6	19F-1098
Linum perenne	PI 445972-7	19F-1099
Linum perenne	PI 445972-8	19F-1100
Linum perenne	PI 445972-9	19F-1101
Linum perenne	PI 445972-10	19F-1102
Linum perenne	PI 445972-11	19F-1103
Linum perenne	PI 445972-12	19F-1104
Linum perenne	PI 445972-13	19F-1105
Linum perenne	PI 445972-14	19F-1106
Linum perenne	PI 445972-15	19F-1107
Linum perenne	PI 445972-16	19F-1108
Linum perenne	PI 445972-18	19F-1109
Linum perenne	PI 445972-20	19F-1110
Linum perenne	CN 107261-1	19F-1647
Linum perenne	CN 107261-4	19F-1649
Linum perenne	CN 19024-1	19F-1778
Linum perenne	CN 19024-2	19F-1779
Linum perenne	CN 19024-3	19F-1780
Linum perenne	CN 19024-4	19F-1781

Linum perenne	CN 19024-5	19F-1782
Linum perenne	CN 19024-6	19F-1783
Linum perenne	CN 19024-7	19F-1784
Linum perenne	CN 19024-8	19F-1785
Linum perenne	CN 19024-9	19F-1786
Linum perenne	CN 19024-10	19F-1787
Linum perenne	CN 19024-11	19F-1788
Linum perenne	CN 19024-12	19F-1789
Linum perenne	CN 19024-13	19F-1790
Linum perenne	CN 19024-14	19F-1791
Linum perenne	CN 107256-1	19F-1792
Linum perenne	CN 107256-2	19F-1793
Linum perenne	CN 107256-3	19F-1794
Linum perenne	CN 107256-4	19F-1795
Linum perenne	CN 107256-5	19F-1796
Linum perenne	CN 107256-6	19F-1797
Linum perenne	CN 107256-7	19F-1798
Linum perenne	CN 107256-8	19F-1799
Linum perenne	CN 107256-10	19F-1800
Linum perenne	CN 107256-11	19F-1801
Linum perenne	CN 107256-12	19F-1802
Linum perenne	CN 107256-13	19F-1803
Linum perenne	CN 107256-14	19F-1804
Linum perenne	CN 107256-15	19F-1805
Linum perenne	CN 107256-16	19F-1806
Linum perenne	CN 107256-17	19F-1807
Linum perenne	CN 107256-18	19F-1808
Linum perenne	CN 107270-1	19F-1809
Linum perenne	CN 107270-2	19F-1810
Linum perenne	CN 107270-4	19F-1811
Linum perenne	CN 107270-5	19F-1812

Linum perenne	CN 107270-6	19F-1813
Linum perenne	CN 107270-7	19F-1814
Linum perenne	CN 107270-8	19F-1815
Linum perenne	CN 107270-9	19F-1816
Linum perenne	CN 107270-10	19F-1817
Linum perenne	CN 107270-11	19F-1818
Linum perenne	CN 107270-13	19F-1819
Linum perenne	CN 107270-14	19F-1820
Linum perenne	CN 107270-15	19F-1821
Linum perenne	CN 107270-16	19F-1822
Linum perenne	CN 107270-17	19F-1823
Linum perenne	CN 107270-18	19F-1824
Linum perenne	CN 107270-19	19F-1825
Linum perenne	CN 107270-20	19F-1826
Linum perenne	PI 650327-1	19F-337
Linum perenne	PI 650327-2	19F-338
Linum perenne	PI 650327-3	19F-339
Linum perenne	PI 650327-4	19F-340
Linum perenne	PI 650327-6	19F-341
Linum perenne	PI 650327-7	19F-342
Linum perenne	PI 650327-8	19F-343
Linum perenne	PI 650327-9	19F-344
Linum perenne	PI 650327-10	19F-345
Linum perenne	PI 650327-11	19F-346
Linum perenne	PI 650327-12	19F-347
Linum perenne	PI 650327-13	19F-348
Linum perenne	PI 650327-14	19F-349
Linum perenne	PI 650327-15	19F-350
Linum perenne	PI 650327-16	19F-351
Linum perenne	PI 650327-17	19F-352
Linum perenne	PI 650327-18	19F-353

Linum perenne	PI 650327-19	19F-354
Linum perenne	PI 650327-20	19F-355
Linum perenne	PI 650328-1	19F-356
Linum perenne	PI 650328-2	19F-357
Linum perenne	PI 650328-3	19F-358
Linum perenne	PI 650328-4	19F-359
Linum perenne	PI 650328-6	19F-360
Linum perenne	PI 650328-7	19F-361
Linum perenne	PI 650328-8	19F-362
Linum perenne	PI 650328-9	19F-363
Linum perenne	PI 650328-10	19F-364
Linum perenne	PI 650328-11	19F-365
Linum perenne	PI 650328-12	19F-366
Linum perenne	PI 650328-13	19F-367
Linum perenne	PI 650328-14	19F-368
Linum perenne	PI 650328-15	19F-369
Linum perenne	PI 650328-16	19F-370
Linum perenne	PI 650328-17	19F-371
Linum perenne	PI 650328-18	19F-372
Linum perenne	PI 650328-19	19F-373
Linum perenne	PI 650328-20	19F-374
Linum perenne	PI 650329-1	19F-375
Linum perenne	PI 650329-2	19F-376
Linum perenne	PI 650329-3	19F-377
Linum perenne	PI 650329-4	19F-378
Linum perenne	PI 650329-5	19F-379
Linum perenne	PI 650329-6	19F-380
Linum perenne	PI 650329-7	19F-381
Linum perenne	PI 650329-8	19F-382
Linum perenne	PI 650329-9	19F-383
Linum perenne	PI 650329-10	19F-384

Linum perenne	PI 650329-11	19F-385
Linum perenne	PI 650329-12	19F-386
Linum perenne	PI 650329-13	19F-387
Linum perenne	PI 650329-14	19F-388
Linum perenne	PI 650329-15	19F-389
Linum perenne	PI 650329-16	19F-390
Linum perenne	PI 650329-18	19F-391
Linum perenne	PI 650329-19	19F-392
Linum perenne	PI 650329-20	19F-393
Linum perenne	Ames 21222-1	19F-499
Linum perenne	Ames 21222-2	19F-500
Linum perenne	Ames 21222-3	19F-501
Linum perenne	Ames 21222-4	19F-502
Linum perenne	Ames 21222-5	19F-503
Linum perenne	Ames 21222-6	19F-504
Linum perenne	Ames 21222-8	19F-505
Linum perenne	Ames 21222-12	19F-506
Linum perenne	Ames 21222-13	19F-507
Linum perenne	Ames 21222-14	19F-508
Linum perenne	Ames 21222-15	19F-509
Linum perenne	Ames 21222-16	19F-510
Linum perenne	Ames 21222-17	19F-511
Linum perenne	Ames 21222-18	19F-512
Linum perenne	Ames 21222-19	19F-513
Linum perenne	Ames 21222-20	19F-514
Linum perenne	Ames 31374-1	19F-515
Linum perenne	Ames 31374-2	19F-516
Linum perenne	Ames 31374-3	19F-517
Linum perenne	Ames 31374-4	19F-518
Linum perenne	Ames 31374-5	19F-519
Linum perenne	Ames 31374-6	19F-520

Linum perenne	Ames 31374-7	19F-521
Linum perenne	Ames 31374-8	19F-522
Linum perenne	Ames 31374-9	19F-523
Linum perenne	Ames 31374-10	19F-524
Linum perenne	Ames 31374-11	19F-525
Linum perenne	Ames 31374-12	19F-526
Linum perenne	Ames 31374-13	19F-527
Linum perenne	Ames 31374-14	19F-528
Linum perenne	Ames 31374-15	19F-529
Linum perenne	Ames 31374-16	19F-530
Linum perenne	Ames 31374-18	19F-532
Linum perenne	Ames 31374-19	19F-533
Linum perenne	Ames 31374-20	19F-534
Linum perenne	PI 650326-1	19F-754
Linum perenne	PI 650326-2	19F-755
Linum perenne	PI 650326-3	19F-756
Linum perenne	PI 650326-5	19F-757
Linum perenne	PI 650326-6	19F-758
Linum perenne	PI 650326-7	19F-759
Linum perenne	PI 650326-8	19F-760
Linum perenne	PI 650326-10	19F-761
Linum perenne	PI 650326-12	19F-762
Linum perenne	PI 650326-13	19F-763
Linum perenne	PI 650326-14	19F-764
Linum perenne	PI 650326-15	19F-765
Linum perenne	PI 650326-16	19F-766
Linum perenne	PI 650326-18	19F-768
Linum perenne	PI 650326-19	19F-769
Linum perenne	PI 650326-20	19F-770
Linum perenne	Blue Flax-1	19F-932
Linum perenne	Blue Flax-2	19F-933

Linum perenne	Blue Flax-3	19F-934
Linum perenne	Blue Flax-4	19F-935
Linum perenne	Blue Flax-5	19F-936
Linum perenne	Blue Flax-6	19F-937
Linum perenne	Blue Flax-7	19F-938
Linum perenne	Blue Flax-8	19F-939
Linum perenne	Blue Flax-10	19F-940
Linum perenne	Blue Flax-11	19F-941
Linum perenne	Blue Flax-12	19F-942
Linum perenne	Blue Flax-13	19F-943
Linum perenne	Blue Flax-15	19F-945
Linum perenne	Blue Flax-16	19F-946
Linum perenne	Blue Flax-17	19F-947
Linum perenne	Blue Flax-19	19F-948
Linum perenne	Blue Flax-20	19F-949

NOTE: This is all of the individuals in the "hybrid cluster"

Table S2 – Sheet 2: *Linum austriacum* nonhybrids

ID	X	Y		sorted for positive Ys for the top pure taxon cluster (yellow); all negatives would be in the lowe[illegible]
19F-170	1.82843	2.510413	PI 650294-12	Yankuli, Stavropol hills, central Caucasus, Russian Federation
19F-204	2.580958	-5.38386	PI 650300-18	Near Tatatszentgyorgy and Orgovany, the Great Hungarian Plain between Duna (Danube) and Tisza Rivers, Hungary
19F-172	3.011518	4.558782	PI 650294-14	Yankuli, Stavropol hills, central Caucasus, Russian Federation
19F-205	3.071823	-5.75345	PI 650300-19	Near Tatatszentgyorgy and Orgovany, the Great Hungarian Plain between Duna (Danube) and Tisza Rivers, Hungary
19F-206	3.325507	-6.5256	PI 650300-20	Near Tatatszentgyorgy and Orgovany, the Great Hungarian Plain between Duna (Danube) and Tisza Rivers, Hungary
19F-203	3.406324	-7.08055	PI 650300-17	Near Tatatszentgyorgy and Orgovany, the Great Hungarian Plain between Duna (Danube) and Tisza Rivers, Hungary
19F-1665	3.409435	-8.13823	CN 107288-6	Wild material, no passport data
19F-196	3.476454	-5.30576	PI 650300-9	Near Tatatszentgyorgy and Orgovany, the Great Hungarian Plain between Duna (Danube) and Tisza Rivers, Hungary
19F-1710	3.478214	-8.33836	PI 440472-7	Division 5, Sheep & Goat Farm, Ravine 4, 52km SE of Stavropol, Russian Federation
19F-1666	3.48195	-8.32901	CN 107288-8	Wild material, no passport data
19F-1708	3.493067	-8.34321	PI 440472-5	Division 5, Sheep & Goat Farm, Ravine 4, 52km SE of Stavropol, Russian Federation
19F-1711	3.512367	-8.32399	PI 440472-9	Division 5, Sheep & Goat Farm, Ravine 4, 52km SE of Stavropol, Russian Federation
19F-1709	3.561159	-8.07519	PI 440472-6	Division 5, Sheep & Goat Farm, Ravine 4, 52km SE of Stavropol, Russian Federation
19F-1668	3.566166	-8.15608	CN 107288-10	Wild material, no passport data
19F-1707	3.57163	-8.41585	PI 440472-1	Division 5, Sheep & Goat Farm, Ravine 4, 52km SE of Stavropol, Russian Federation
19F-198	3.593367	-5.9315	PI 650300-12	Near Tatatszentgyorgy and Orgovany, the Great Hungarian Plain between Duna (Danube) and Tisza Rivers, Hungary
19F-1671	3.639809	-8.14671	CN 107288-13	Wild material, no passport data
19F-1670	3.64971	-8.13162	CN 107288-12	Wild material, no passport data
19F-1669	3.662393	-8.33896	CN 107288-11	Wild material, no passport data
19F-1667	3.761633	-8.27993	CN 107288-9	Wild material, no passport data
19F-1672	3.788779	-8.19979	CN 107288-14	Wild material, no passport data
19F-175	3.800549	4.314707	PI 650294-17	Yankuli, Stavropol hills, central Caucasus, Russian Federation

19F-195	3.806291	-6.78745	PI 650300-8	Near Tatatszentgyorgy and Orgovany,, the Great Hungarian Plain between Duna (Danube) and Tisza Rivers, Hungary
19F-202	3.869636	-7.02095	PI 650300-16	Near Tatatszentgyorgy and Orgovany,, the Great Hungarian Plain between Duna (Danube) and Tisza Rivers, Hungary
19F-193	3.889428	-6.09129	PI 650300-5	Near Tatatszentgyorgy and Orgovany,, the Great Hungarian Plain between Duna (Danube) and Tisza Rivers, Hungary
19F-171	3.897632	5.551877	PI 650294-13	Yankuli, Stavropol hills, central Caucasus, Russian Federation
19F-191	3.94473	-6.53005	PI 650300-2	Near Tatatszentgyorgy and Orgovany,, the Great Hungarian Plain between Duna (Danube) and Tisza Rivers, Hungary
19F-200	3.984481	-6.06752	PI 650300-14	Near Tatatszentgyorgy and Orgovany,, the Great Hungarian Plain between Duna (Danube) and Tisza Rivers, Hungary
19F-229	3.994105	-7.71794	PI 650296-6	above Albena, Dobrich, Bulgaria
19F-199	4.063869	-6.11533	PI 650300-13	Near Tatatszentgyorgy and Orgovany,, the Great Hungarian Plain between Duna (Danube) and Tisza Rivers, Hungary
19F-161	4.208326	3.333272	PI 650294-2	Yankuli, Stavropol hills, central Caucasus, Russian Federation
19F-201	4.215318	-6.23977	PI 650300-15	Near Tatatszentgyorgy and Orgovany,, the Great Hungarian Plain between Duna (Danube) and Tisza Rivers, Hungary
19F-194	4.341557	-6.18761	PI 650300-6	Near Tatatszentgyorgy and Orgovany,, the Great Hungarian Plain between Duna (Danube) and Tisza Rivers, Hungary
19F-184	4.623118	-8.24203	PI 650299-12	Along roadside near Kaliakra, close to the Black Sea, Dobrich, Bulgaria
19F-197	4.653536	-6.36129	PI 650300-11	Near Tatatszentgyorgy and Orgovany,, the Great Hungarian Plain between Duna (Danube) and Tisza Rivers, Hungary
19F-192	4.847271	-6.72244	PI 650300-4	Near Tatatszentgyorgy and Orgovany,, the Great Hungarian Plain between Duna (Danube) and Tisza Rivers, Hungary
19F-231	4.869807	-7.79015	PI 650296-9	above Albena, Dobrich, Bulgaria
19F-228	4.992906	-8.46573	PI 650296-5	above Albena, Dobrich, Bulgaria
19F-182	5.171035	-8.82286	PI 650299-8	Along roadside near Kaliakra, close to the Black Sea, Dobrich, Bulgaria
19F-226	5.268392	-9.77027	PI 650296-3	above Albena, Dobrich, Bulgaria
19F-189	5.686977	-8.21076	PI 650299-18	Along roadside near Kaliakra, close to the Black Sea, Dobrich, Bulgaria
19F-167	5.789956	4.789597	PI 650294-8	Yankuli, Stavropol hills, central Caucasus, Russian Federation
19F-237	5.825835	-9.59521	PI 650296-15	above Albena, Dobrich, Bulgaria
19F-233	5.914249	-8.887	PI 650296-11	above Albena, Dobrich, Bulgaria
19F-163	6.105814	5.278111	PI 650294-4	Yankuli, Stavropol hills, central Caucasus, Russian Federation

19F-238	6.183976	-9.12072	PI 650296-16	above Albena, Dobrich, Bulgaria
19F-179	6.210416	-9.80902	PI 650299-1	Along roadside near Kaliakra, close to the Black Sea, Dobrich, Bulgaria
19F-234	6.210925	-10.6996	PI 650296-12	above Albena, Dobrich, Bulgaria
19F-239	6.240084	-10.3312	PI 650296-17	above Albena, Dobrich, Bulgaria
19F-169	6.247467	6.365638	PI 650294-10	Yankuli, Stavropol hills, central Caucasus, Russian Federation
19F-232	6.252154	-9.11457	PI 650296-10	above Albena, Dobrich, Bulgaria
19F-180	6.280111	-8.91386	PI 650299-4	Along roadside near Kaliakra, close to the Black Sea, Dobrich, Bulgaria
19F-185	6.333907	-9.40794	PI 650299-14	Along roadside near Kaliakra, close to the Black Sea, Dobrich, Bulgaria
19F-779	6.498349	-10.9628	PI 650298-13	Along roadside near Kaliakra, close to the Black Sea, Dobrich, Bulgaria
19F-225	6.502232	-10.4895	PI 650296-2	above Albena, Dobrich, Bulgaria
19F-230	6.529567	-10.2496	PI 650296-7	above Albena, Dobrich, Bulgaria
19F-778	6.552594	-12.0315	PI 650298-12	Rocky hillside with a gentle slope, S-SW exposure. Alkalai soils (pH 9.5), Plovdiv, Bulgaria
19F-236	6.558853	-9.86344	PI 650296-14	above Albena, Dobrich, Bulgaria
19F-240	6.560599	-9.93762	PI 650296-18	above Albena, Dobrich, Bulgaria
19F-788	6.578896	-11.2499	PI 650298-22	Rocky hillside with a gentle slope, S-SW exposure. Alkalai soils (pH 9.5), Plovdiv, Bulgaria
19F-174	6.635664	4.816848	PI 650294-16	Yankuli, Stavropol hills, central Caucasus, Russian Federation
19F-190	6.727648	-9.93705	PI 650299-19	Along roadside near Kaliakra, close to the Black Sea, Dobrich, Bulgaria
19F-771	6.755727	-11.3185	PI 650298-1	Rocky hillside with a gentle slope, S-SW exposure. Alkalai soils (pH 9.5), Plovdiv, Bulgaria
19F-787	6.769199	-11.1715	PI 650298-21	Rocky hillside with a gentle slope, S-SW exposure. Alkalai soils (pH 9.5), Plovdiv, Bulgaria
19F-162	6.793954	4.883493	PI 650294-3	Yankuli, Stavropol hills, central Caucasus, Russian Federation
19F-776	6.8231	-11.0799	PI 650298-10	Rocky hillside with a gentle slope, S-SW exposure. Alkalai soils (pH 9.5), Plovdiv, Bulgaria
19F-783	6.857547	-11.3841	PI 650298-17	Rocky hillside with a gentle slope, S-SW exposure. Alkalai soils (pH 9.5), Plovdiv, Bulgaria
19F-780	6.859853	-11.4729	PI 650298-14	Rocky hillside with a gentle slope, S-SW exposure. Alkalai soils (pH 9.5), Plovdiv, Bulgaria
19F-187	6.916951	-10.0796	PI 650299-16	Along roadside near Kaliakra, close to the Black Sea, Dobrich, Bulgaria
19F-786	6.958319	-11.6095	PI 650298-20	Rocky hillside with a gentle slope, S-SW exposure. Alkalai soils (pH 9.5), Plovdiv, Bulgaria
19F-772	7.017397	-11.2086	PI 650298-6	Rocky hillside with a gentle slope, S-SW exposure. Alkalai soils (pH 9.5), Plovdiv, Bulgaria
19F-774	7.027994	-11.1564	PI 650298-8	Rocky hillside with a gentle slope, S-SW exposure. Alkalai soils (pH 9.5), Plovdiv, Bulgaria
19F-183	7.043137	-10.1623	PI 650299-10	Along roadside near Kaliakra, close to the Black Sea, Dobrich, Bulgaria
19F-785	7.04726	-10.945	PI 650298-19	Rocky hillside with a gentle slope, S-SW exposure. Alkalai soils (pH 9.5), Plovdiv, Bulgaria
19F-178	7.052718	5.209998	PI 650294-20	Yankuli, Stavropol hills, central Caucasus, Russian Federation
19F-784	7.067653	-11.4772	PI 650298-18	Rocky hillside with a gentle slope, S-SW exposure. Alkalai soils (pH 9.5), Plovdiv, Bulgaria

19F-165	7.069114	5.636772	PI 650294-6	Yankuli, Stavropol hills, central Caucasus, Russian Federation
19F-777	7.073199	-10.8512	PI 650298-11	Rocky hillside with a gentle slope, S-SW exposure. Alkalai soils (pH 9.5), Plovdiv, Bulgaria
19F-235	7.14904	-10.3563	PI 650296-13	Rocky hillside with a gentle slope, S-SW exposure. Alkalai soils (pH 9.5), Plovdiv, Bulgaria
19F-773	7.150842	-12.4302	PI 650298-7	Rocky hillside with a gentle slope, S-SW exposure. Alkalai soils (pH 9.5), Plovdiv, Bulgaria
19F-188	7.153881	-9.82038	PI 650299-17	Along roadside near Kaliakra, close to the Black Sea, Dobrich, Bulgaria
19F-775	7.177075	-11.8397	PI 650298-9	Rocky hillside with a gentle slope, S-SW exposure. Alkalai soils (pH 9.5), Plovdiv, Bulgaria
19F-781	7.182394	-12.3488	PI 650298-15	Rocky hillside with a gentle slope, S-SW exposure. Alkalai soils (pH 9.5), Plovdiv, Bulgaria
19F-782	7.183536	-11.8307	PI 650298-16	Rocky hillside with a gentle slope, S-SW exposure. Alkalai soils (pH 9.5), Plovdiv, Bulgaria
19F-181	7.380352	-10.1489	PI 650299-6	Along roadside near Kaliakra, close to the Black Sea, Dobrich, Bulgaria
19F-177	7.567126	4.992136	PI 650294-19	Yankuli, Stavropol hills, central Caucasus, Russian Federation
19F-186	7.623017	-9.95576	PI 650299-15	Along roadside near Kaliakra, close to the Black Sea, Dobrich, Bulgaria
19F-173	7.685656	3.998954	PI 650294-15	Yankuli, Stavropol hills, central Caucasus, Russian Federation
19F-100	8.293054	3.307444	PI 650301-8	Near Jil, Gegharkunik Marz, Armenia
19F-108	8.375809	3.426992	PI 650301-20	Near Jil, Gegharkunik Marz, Armenia
19F-168	8.388267	5.197662	PI 650294-9	Yankuli, Stavropol hills, central Caucasus, Russian Federation
19F-125	8.463146	4.30013	Ames 29749-3	Opuk National Preserve, 20 km southeast of Marfivka, Ukraine
19F-98	8.529219	3.466327	PI 650301-6	Near Jil, Gegharkunik Marz, Armenia
19F-106	8.534933	3.836947	PI 650301-18	Near Jil, Gegharkunik Marz, Armenia
19F-97	8.609585	3.190929	PI 650301-4	Near Jil, Gegharkunik Marz, Armenia
19F-107	8.610059	3.173307	PI 650301-19	Near Jil, Gegharkunik Marz, Armenia
19F-129	8.643056	4.753846	Ames 29749-8	Opuk National Preserve, 20 km southeast of Marfivka, Ukraine
19F-99	8.71985	3.629846	PI 650301-7	Near Jil, Gegharkunik Marz, Armenia
19F-104	8.73552	3.289827	PI 650301-15	Near Jil, Gegharkunik Marz, Armenia
19F-96	8.78499	3.356305	PI 650301-1	Near Jil, Gegharkunik Marz, Armenia
19F-101	8.808962	3.540755	PI 650301-10	Near Jil, Gegharkunik Marz, Armenia
19F-102	8.891746	3.063821	PI 650301-13	Near Jil, Gegharkunik Marz, Armenia
19F-103	8.949089	3.429096	PI 650301-14	Near Jil, Gegharkunik Marz, Armenia
19F-131	9.001613	4.950144	Ames 29749-10	Opuk National Preserve, 20 km southeast of Marfivka, Ukraine
19F-136	9.123618	5.437795	Ames 29749-16	Opuk National Preserve, 20 km southeast of Marfivka, Ukraine

19F-132	9.214548	5.574792	Ames 29749-11	Opuk National Preserve, 20 km southeast of Marfivka, Ukraine
19F-176	9.287154	4.546578	PI 650294-18	Yankuli, Stavropol hills, central Caucasus, Russian Federation
19F-135	9.343345	5.196106	Ames 29749-15	Opuk National Preserve, 20 km southeast of Marfivka, Ukraine
19F-123	9.395751	5.411065	Ames 29749-1	Opuk National Preserve, 20 km southeast of Marfivka, Ukraine
19F-166	9.514784	5.133895	PI 650294-7	Yankuli, Stavropol hills, central Caucasus, Russian Federation
19F-134	9.5692	5.829631	Ames 29749-14	Opuk National Preserve, 20 km southeast of Marfivka, Ukraine
19F-31	9.635225	7.148608	PI 502410-14	West, 250km of Elista, Kalmyk ARR Russian Federation
19F-138	9.70523	5.602839	Ames 29749-19	Opuk National Preserve, 20 km southeast of Marfivka, Ukraine
19F-127	9.72505	5.878376	Ames 29749-5	Opuk National Preserve, 20 km southeast of Marfivka, Ukraine
19F-133	9.737934	5.428748	Ames 29749-12	Opuk National Preserve, 20 km southeast of Marfivka, Ukraine
19F-130	9.811036	6.021393	Ames 29749-9	Opuk National Preserve, 20 km southeast of Marfivka, Ukraine
19F-5	9.85467	5.443542	PI 502405-5	Steppe, Askania-Nova Preserve, Ukraine
19F-128	9.877043	6.405153	Ames 29749-7	Opuk National Preserve, 20 km southeast of Marfivka, Ukraine
19F-16	9.898054	5.884945	PI 502405-19	Steppe, Askania-Nova Preserve, Ukraine
19F-17	9.934896	4.790996	PI 502405-20	Steppe, Askania-Nova Preserve, Ukraine
19F-137	9.976577	5.866197	Ames 29749-17	Opuk National Preserve, 20 km southeast of Marfivka, Ukraine
19F-21	10.23032	6.40065	PI 502410-4	West, 250km of Elista, Kalmyk ARR Russian Federation
19F-1718	10.24915	6.270392	PI 440472-17	Division 5, Sheep & Goat Farm, Ravine 4, 52km SE of Stavropol, Russian Federation
19F-1716	10.34261	6.360195	PI 440472-14	Division 5, Sheep & Goat Farm, Ravine 4, 52km SE of Stavropol, Russian Federation
19F-1713	10.4352	6.761519	PI 440472-11	Division 5, Sheep & Goat Farm, Ravine 4, 52km SE of Stavropol, Russian Federation
19F-10	10.45073	5.929765	PI 502405-11	Steppe, Askania-Nova Preserve, Ukraine
19F-1712	10.46489	6.353941	PI 440472-10	Division 5, Sheep & Goat Farm, Ravine 4, 52km SE of Stavropol, Russian Federation
19F-14	10.49403	6.222952	PI 502405-16	Steppe, Askania-Nova Preserve, Ukraine
19F-146	10.58036	6.53133	PI 440473-11	Beshpagin Sands, 44km southeast of Stavropol, Russian Federation
19F-18	10.58309	6.916456	PI 502410-1	West, 250km of Elista, Kalmyk ARR Russian Federation
19F-12	10.59034	6.140631	PI 502405-13	Steppe, Askania-Nova Preserve, Ukraine
19F-4	10.59259	6.175617	PI 502405-4	Steppe, Askania-Nova Preserve, Ukraine
19F-3	10.59883	5.321727	PI 502405-3	Steppe, Askania-Nova Preserve, Ukraine

19F-11	10.61505	6.327481	PI 502405-12	Steppe, Askania-Nova Preserve, Ukraine
19F-9	10.66037	6.312192	PI 502405-10	Steppe, Askania-Nova Preserve, Ukraine
19F-24	10.68999	7.360933	PI 502410-7	West, 250km of Elista, Kalmyk ARR Russian Federation
19F-7	10.69886	6.063113	PI 502405-7	Steppe, Askania-Nova Preserve, Ukraine
19F-28	10.73223	7.379081	PI 502410-11	West, 250km of Elista, Kalmyk ARR Russian Federation
19F-15	10.74202	5.681497	PI 502405-17	Steppe, Askania-Nova Preserve, Ukraine
19F-1	10.74522	6.067308	PI 502405-1	Steppe, Askania-Nova Preserve, Ukraine
19F-8	10.79925	6.149478	PI 502405-9	Steppe, Askania-Nova Preserve, Ukraine
19F-150	10.81523	7.135966	PI 440473-17	Beshpagin Sands, 44km southeast of Stavropol, Russian Federation
19F-152	10.83833	6.762848	PI 440473-19	Beshpagin Sands, 44km southeast of Stavropol, Russian Federation
19F-147	10.89548	6.700188	PI 440473-13	Beshpagin Sands, 44km southeast of Stavropol, Russian Federation
19F-148	10.91002	6.852918	PI 440473-14	Beshpagin Sands, 44km southeast of Stavropol, Russian Federation
19F-139	10.94918	5.833585	PI 440473-1	Beshpagin Sands, 44km southeast of Stavropol, Russian Federation
19F-23	10.96086	7.25547	PI 502410-6	West, 250km of Elista, Kalmyk ARR Russian Federation
19F-20	10.96209	6.952391	PI 502410-3	West, 250km of Elista, Kalmyk ARR Russian Federation
19F-1714	10.96252	6.523035	PI 440472-12	Division 5, Sheep & Goat Farm, Ravine 4, 52km SE of Stavropol, Russian Federation
19F-141	10.96929	6.732931	PI 440473-3	Beshpagin Sands, 44km southeast of Stavropol, Russian Federation
19F-6	10.98448	6.198505	PI 502405-6	Steppe, Askania-Nova Preserve, Ukraine
19F-149	10.99082	7.119913	PI 440473-16	Beshpagin Sands, 44km southeast of Stavropol, Russian Federation
19F-151	10.99086	6.511578	PI 440473-18	Beshpagin Sands, 44km southeast of Stavropol, Russian Federation
19F-1715	11.00366	6.630582	PI 440472-13	Division 5, Sheep & Goat Farm, Ravine 4, 52km SE of Stavropol, Russian Federation
19F-26	11.01134	7.016679	PI 502410-9	West, 250km of Elista, Kalmyk ARR Russian Federation
19F-153	11.0417	6.690534	PI 440473-20	Beshpagin Sands, 44km southeast of Stavropol, Russian Federation
19F-1717	11.0688	7.367447	PI 440472-16	Division 5, Sheep & Goat Farm, Ravine 4, 52km SE of Stavropol, Russian Federation
19F-140	11.12913	6.752604	PI 440473-2	Beshpagin Sands, 44km southeast of Stavropol, Russian Federation
19F-27	11.16523	7.03808	PI 502410-10	West, 250km of Elista, Kalmyk ARR Russian Federation
19F-144	11.16549	6.832028	PI 440473-9	Beshpagin Sands, 44km southeast of Stavropol, Russian Federation
19F-142	11.17614	6.260318	PI 440473-6	Beshpagin Sands, 44km southeast of Stavropol, Russian Federation
19F-25	11.18925	7.591532	PI 502410-8	West, 250km of Elista, Kalmyk ARR Russian Federation
19F-145	11.20696	7.229028	PI 440473-10	Beshpagin Sands, 44km southeast of Stavropol, Russian Federation
19F-33	11.2156	7.038182	PI 502410-18	West, 250km of Elista, Kalmyk ARR Russian Federation

19F-32	11.43161	7.730515	PI 502410-15	West, 250km of Elista, Kalmyk ARR Russian Federation
19F-143	11.48042	6.911452	PI 440473-7	Beshpagin Sands, 44km southeast of Stavropol, Russian Federation
19F-29	11.55944	7.226443	PI 502410-12	West, 250km of Elista, Kalmyk ARR Russian Federation
19F-34	11.56393	7.470999	PI 502410-19	West, 250km of Elista, Kalmyk ARR Russian Federation
19F-19	11.69687	6.996209	PI 502410-2	West, 250km of Elista, Kalmyk ARR Russian Federation
19F-35	11.8062	7.592266	PI 502410-20	West, 250km of Elista, Kalmyk ARR Russian Federation
19F-22	11.83904	7.422438	PI 502410-5	West, 250km of Elista, Kalmyk ARR Russian Federation

Table S2 – Sheet 3: *Linum lewisii* nonhybrids

id	X	Y		sorted for positive Ys for the top pure taxon cluster (yellow); all negatives would be in the lower cluster
19F-1061	1.74698	2.017117	CN 107266-1	Origin uncertain, N. America
19F-1062	1.768126	2.125344	CN 107266-2	Origin uncertain, N. America
19F-1063	2.018983	2.348309	CN 107266-3	Origin uncertain, N. America
19F-1064	1.995991	2.12298	CN 107266-4	Origin uncertain, N. America
19F-1065	1.988324	2.002364	CN 107266-5	Origin uncertain, N. America
19F-1066	1.988324	2.002364	CN 107266-6	Origin uncertain, N. America
19F-1067	1.541594	2.366243	CN 107266-7	Origin uncertain, N. America
19F-1068	1.903609	2.344113	CN 107266-8	Origin uncertain, N. America
19F-1069	2.00129	2.111408	CN 107266-9	Origin uncertain, N. America
19F-1070	2.032359	2.335623	CN 107266-10	Origin uncertain, N. America
19F-1071	2.037247	2.44578	CN 107266-11	Origin uncertain, N. America
19F-1072	1.988324	2.002364	CN 107266-12	Origin uncertain, N. America
19F-1563	1.781365	-1.25236	Ames 31371-1	Origin uncertain, N. America
19F-1564	1.501244	-1.62247	Ames 31371-2	Origin uncertain, N. America
19F-1565	1.729611	-1.72638	Ames 31371-4	Origin uncertain, N. America

19F-1566	1.737689	-1.72749	Ames 31371-6	Origin uncertain, N. America
19F-1567	1.850283	-1.73375	Ames 31371-8	Origin uncertain, N. America
19F-1568	1.850283	-1.73375	Ames 31371-9	Origin uncertain, N. America
19F-1569	1.374538	-1.6937	Ames 31371-10	Origin uncertain, N. America
19F-1570	1.970955	-1.74113	Ames 31371-14	Origin uncertain, N. America
19F-1571	1.858361	-1.73487	Ames 31371-15	Origin uncertain, N. America
19F-1572	1.742057	-1.72681	Ames 31372-1	Wyoming, USA
19F-1573	1.858463	-1.73457	Ames 31372-7	Wyoming, USA
19F-1574	1.850283	-1.73375	Ames 31372-10	Wyoming, USA
19F-1575	1.367596	-1.70425	Ames 31372-11	Wyoming, USA
19F-1576	2.037247	2.44578	Ames 32565-1	Wyoming, USA
19F-1578	2.130552	2.218939	Ames 32565-4	Wyoming, USA
19F-1579	1.988324	2.002364	Ames 32565-5	Wyoming, USA
19F-1580	1.815091	2.704273	Ames 32565-6	Wyoming, USA
19F-1581	2.157762	2.201656	Ames 32565-7	Wyoming, USA
19F-1582	2.074124	2.788775	Ames 32565-8	Wyoming, USA
19F-1583	2.028443	2.336858	Ames 32565-9	Wyoming, USA

19F-1584	2.210461	2.87613	Ames 32565-10	Wyoming, USA
19F-1585	2.144953	2.32936	Ames 32565-11	Wyoming, USA
19F-1586	2.144953	2.32936	Ames 32565-12	Wyoming, USA
19F-1587	2.073603	2.679303	Ames 32565-13	Wyoming, USA
19F-1588	1.988324	2.002364	Ames 32565-14	Wyoming, USA
19F-1589	1.953452	2.796152	Ames 32565-15	Wyoming, USA
19F-1590	2.089789	2.883507	Ames 32565-16	Wyoming, USA
19F-1591	2.073603	2.679303	Ames 32565-17	Wyoming, USA
19F-1592	1.916575	2.453157	Ames 32565-18	Wyoming, USA
19F-1593	2.074124	2.788775	Ames 32565-19	Wyoming, USA
19F-1594	1.748399	2.904391	Ames 32565-20	Wyoming, USA
19F-1595	1.970955	-1.74113	Ames 32566-1	Utah, USA
19F-1596	1.75905	-1.63856	Ames 32566-2	Utah, USA
19F-1597	1.608939	-1.719	Ames 32566-3	Utah, USA
19F-1598	1.653278	-1.7222	Ames 32566-4	Utah, USA
19F-1599	1.874099	-1.73542	Ames 32566-6	Utah, USA
19F-1600	1.970955	-1.74113	Ames 32566-7	Utah, USA

19F-1601	1.496345	-1.71274	Ames 32566-8	Utah, USA
19F-1602	1.851565	-1.73534	Ames 32566-9	Utah, USA
19F-1603	1.186736	-1.22221	Ames 32566-10	Utah, USA
19F-1604	1.970955	-1.74113	Ames 32566-11	Utah, USA
19F-1605	1.512659	-1.71507	Ames 32566-14	Utah, USA
19F-1606	1.621385	-1.71943	Ames 32566-16	Utah, USA
19F-1625	1.742057	-1.72681	CN 107300-2	Origin uncertain, N. America
19F-1626	1.850283	-1.73375	CN 107300-4	Origin uncertain, N. America
19F-261	1.799286	2.42002	PI 522306-1	Origin uncertain, N. America
19F-262	1.969117	2.890884	PI 522306-2	Origin uncertain, N. America
19F-263	1.740219	2.905208	PI 522306-3	Origin uncertain, N. America
19F-264	1.903609	2.344113	PI 522306-4	Origin uncertain, N. America
19F-266	0.284943	2.877459	PI 522306-6	Origin uncertain, N. America
19F-267	1.536899	2.326842	PI 522306-7	Origin uncertain, N. America
19F-268	2.070074	2.76909	PI 522306-8	Origin uncertain, N. America
19F-269	0.941762	2.363642	PI 522306-9	Origin uncertain, N. America
19F-270	2.210461	2.87613	PI 522306-10	Origin uncertain, N. America
19F-271	1.848445	2.89826	PI 522306-11	Origin uncertain, N. America
19F-272	1.940486	2.687108	PI 522306-12	Origin uncertain, N. America
19F-273	2.210461	2.87613	PI 522306-13	Origin uncertain, N. America
19F-274	1.815185	2.515079	PI 522306-14	Origin uncertain, N. America
19F-275	2.061158	2.679732	PI 522306-15	Origin uncertain, N. America
19F-276	1.957575	2.775437	PI 522306-17	Origin uncertain, N. America
19F-277	1.832911	2.802665	PI 522306-19	Origin uncertain, N. America

19F-278	2.089789	2.883507	PI 522306-20	Origin uncertain, N. America
19F-279	2.032359	2.335623	PI 650320-1	Utah, USA
19F-280	2.18183	2.672355	PI 650320-2	Utah, USA
19F-281	0.525354	1.733418	PI 650320-3	Utah, USA
19F-282	1.711588	2.701433	PI 650320-4	Utah, USA
19F-283	2.036727	2.336308	PI 650320-5	Utah, USA
19F-284	2.144953	2.32936	PI 650320-6	Utah, USA
19F-285	1.953452	2.796152	PI 650320-8	Utah, USA
19F-286	2.033197	2.426095	PI 650320-10	Utah, USA
19F-287	1.707322	2.701045	PI 650320-11	Utah, USA
19F-288	2.144953	2.32936	PI 650320-12	Utah, USA
19F-289	1.212371	2.384133	PI 650320-13	Utah, USA
19F-290	2.061158	2.679732	PI 650320-14	Utah, USA
19F-291	2.190746	2.761714	PI 650320-15	Utah, USA
19F-292	1.603882	2.817854	PI 650320-16	Utah, USA
19F-293	1.981562	2.890455	PI 650320-17	Utah, USA
19F-294	1.638814	2.024291	PI 650320-19	Utah, USA
19F-295	1.845225	2.8031	PI 650320-20	Utah, USA
19F-309	1.613101	-1.71888	Ames 32568-6	Wyoming, USA
19F-310	1.612365	-0.78031	Ames 32568-8	Wyoming, USA
19F-311	1.850283	-1.73375	Ames 32568-9	Wyoming, USA
19F-312	1.738527	-1.63702	Ames 32568-15	Wyoming, USA
19F-313	1.733672	-1.72658	Ames 33352-1	Nevada, USA
19F-314	1.850283	-1.73375	Ames 33352-3	Nevada, USA
19F-315	1.492328	-1.71183	Ames 33352-6	Nevada, USA
19F-316	1.970955	-1.74113	Ames 33352-7	Nevada, USA
19F-317	1.733672	-1.72658	Ames 33352-10	Nevada, USA
19F-318	1.488268	-1.71162	Ames 33352-13	Nevada, USA
19F-319	1.621077	-1.72032	Ames 33352-14	Nevada, USA

19F-320	1.733672	-1.72658	Ames 33352-15	Nevada, USA
19F-321	1.617855	-1.62964	Ames 33352-18	Nevada, USA
19F-322	1.970955	-1.74113	Ames 33352-20	Nevada, USA
19F-325	1.970955	-1.74113	Ames 33353-4	Arizona, USA
19F-326	1.970955	-1.74113	Ames 33353-6	Arizona, USA
19F-327	1.970955	-1.74113	Ames 33353-8	Arizona, USA
19F-328	1.970955	-1.74113	Ames 33353-9	Arizona, USA
19F-329	1.970955	-1.74113	Ames 33353-10	Arizona, USA
19F-330	1.970955	-1.74113	Ames 33353-11	Arizona, USA
19F-331	1.850453	-1.73597	Ames 33353-12	Arizona, USA
19F-332	1.970955	-1.74113	Ames 33353-13	Arizona, USA
19F-333	1.850283	-1.73375	Ames 33353-15	Arizona, USA
19F-334	1.970955	-1.74113	Ames 33353-16	Arizona, USA
19F-335	1.73807	-1.7314	Ames 33353-17	Arizona, USA
19F-336	1.901685	-1.26897	Ames 33353-20	Arizona, USA
19F-394	1.979871	-1.65177	Ames 31366-7	Arizona, USA
19F-395	1.979871	-1.65177	Ames 31366-9	Arizona, USA
19F-396	1.970955	-1.74113	Ames 31366-10	Arizona, USA
19F-397	1.850283	-1.73375	Ames 31366-13	Arizona, USA
19F-398	2.030831	-1.19069	Ames 31366-15	Arizona, USA
19F-399	1.979871	-1.65177	Ames 31366-16	Arizona, USA
19F-400	1.851565	-1.73534	Ames 31366-17	Arizona, USA

19F-401	1.500406	-1.71294	Ames 31369-2	Nevada, USA
19F-402	1.965657	-1.72956	Ames 31369-4	Nevada, USA
19F-403	1.850283	-1.73375	Ames 31369-8	Nevada, USA
19F-404	1.733672	-1.72658	Ames 31369-9	Nevada, USA
19F-405	1.970955	-1.74113	Ames 31369-12	Nevada, USA
19F-406	1.850283	-1.73375	Ames 31369-15	Nevada, USA
19F-451	1.970955	-1.74113	Ames 31365-1	Colorado, USA
19F-452	1.850283	-1.73375	Ames 31365-2	Colorado, USA
19F-453	2.021916	-1.28005	Ames 31365-5	Colorado, USA
19F-454	1.618532	-1.72138	Ames 31365-8	Colorado, USA
19F-455	1.850283	-1.73375	Ames 31365-10	Colorado, USA
19F-456	2.021916	-1.28005	Ames 31365-11	Colorado, USA
19F-457	1.965657	-1.72956	Ames 31365-14	Colorado, USA
19F-458	2.021916	-1.28005	Ames 31365-17	Colorado, USA
19F-459	1.729611	-1.72638	Ames 31365-18	Colorado, USA
19F-460	1.60946	-1.71804	Ames 31370-1	Origin uncertain, N. America
19F-461	1.970955	-1.74113	Ames 31370-2	Origin uncertain, N. America
19F-462	1.770695	-1.87243	Ames 31370-6	Origin uncertain, N. America
19F-463	1.850283	-1.73375	Ames 31370-11	Origin uncertain, N. America
19F-465	1.729611	-1.72638	Ames 31370-13	Origin uncertain, N. America
19F-466	1.970955	-1.74113	Ames 31370-14	Origin uncertain, N. America
19F-467	1.970955	-1.74113	Ames 31370-16	Origin uncertain, N. America
19F-468	1.970955	-1.74113	Ames 31370-17	Origin uncertain, N. America
19F-469	1.850588	-1.73528	Ames 31370-19	Origin uncertain, N. America

19F-470	2.170728	2.3107	Ames 32565-1	Wyoming, USA
19F-471	2.061679	2.68069	Ames 32565-3	Wyoming, USA
19F-472	2.194796	2.781399	Ames 32565-4	Wyoming, USA
19F-473	1.83226	2.694057	Ames 32565-5	Wyoming, USA
19F-474	2.18183	2.672355	Ames 32565-6	Wyoming, USA
19F-475	1.754127	2.028261	Ames 32565-7	Wyoming, USA
19F-476	2.144953	2.32936	Ames 32565-8	Wyoming, USA
19F-477	1.906389	2.354573	Ames 32565-9	Wyoming, USA
19F-478	2.18183	2.672355	Ames 32565-10	Wyoming, USA
19F-479	2.130552	2.218939	Ames 32565-11	Wyoming, USA
19F-480	2.061158	2.679732	Ames 32565-12	Wyoming, USA
19F-481	2.157919	2.438404	Ames 32565-13	Wyoming, USA
19F-482	2.024281	2.336736	Ames 32565-14	Wyoming, USA
19F-483	1.940486	2.687108	Ames 32565-15	Wyoming, USA
19F-484	2.05586	2.691304	Ames 32565-16	Wyoming, USA
19F-485	2.031949	2.457353	Ames 32565-17	Wyoming, USA
19F-486	2.18183	2.672355	Ames 32565-18	Wyoming, USA
19F-487	2.082304	2.787958	Ames 32565-19	Wyoming, USA
19F-488	2.18183	2.672355	Ames 32565-20	Wyoming, USA
19F-553	1.608939	-1.719	Ames 33354-1	Nevada, USA
19F-554	1.970955	-1.74113	Ames 33354-2	Nevada, USA
19F-555	1.613	-1.71921	Ames 33354-6	Nevada, USA
19F-556	1.729611	-1.72638	Ames 33354-9	Nevada, USA
19F-557	1.729611	-1.72638	Ames 33354-10	Nevada, USA

19F-558	1.500406	-1.71294	Ames 33354-14	Nevada, USA
19F-559	1.850283	-1.73375	Ames 33354-17	Nevada, USA
19F-56	0.89335	-1.67639	Ames 27614-1	Utah, USA
19F-560	1.492328	-1.71183	Ames 33354-19	Nevada, USA
19F-561	2.144953	2.32936	PI 522305-1	unknown origin, N. America
19F-562	2.037247	2.44578	PI 522305-2	unknown origin, N. America
19F-563	2.144953	2.32936	PI 522305-3	unknown origin, N. America
19F-564	2.144953	2.32936	PI 522305-4	unknown origin, N. America
19F-565	1.988324	2.002364	PI 522305-6	unknown origin, N. America
19F-566	1.944648	2.68723	PI 522305-8	unknown origin, N. America
19F-567	2.061158	2.679732	PI 522305-9	unknown origin, N. America
19F-568	2.025563	2.335146	PI 522305-12	unknown origin, N. America
19F-569	1.903609	2.344113	PI 522305-13	unknown origin, N. America
19F-57	1.859199	-1.6444	Ames 27614-2	unknown origin, N. America
19F-570	2.18183	2.672355	PI 522305-15	unknown origin, N. America
19F-571	1.605383	1.918104	PI 522305-16	unknown origin, N. America
19F-572	2.144953	2.32936	PI 522305-17	unknown origin, N. America
19F-573	1.634262	-1.73185	PI 452487-1	Montana, USA
19F-574	1.392919	-1.71709	PI 452487-2	Montana, USA
19F-575	1.634262	-1.73185	PI 452487-4	Montana, USA
19F-576	0.910231	-1.68759	PI 452487-5	Montana, USA
19F-577	1.513571	-1.72605	PI 452487-6	Montana, USA
19F-578	1.522506	-1.63511	PI 452487-7	Montana, USA
19F-579	1.272247	-1.70972	PI 452487-8	Montana, USA
19F-580	1.28042	-1.71075	PI 452487-9	Montana, USA
19F-581	1.634262	-1.73185	PI 452487-10	Montana, USA
19F-582	1.16402	-1.70277	PI 452487-11	Montana, USA
19F-583	1.272247	-1.70972	PI 452487-13	Montana, USA
19F-584	1.522506	-1.63511	PI 452487-17	Montana, USA

19F-59	1.383949	-1.7061	Ames 27614-4	Utah, USA ('Maple Grove')
19F-60	1.608939	-1.719	Ames 27614-5	Utah, USA ('Maple Grove')
19F-61	1.850283	-1.73375	Ames 27614-6	Utah, USA ('Maple Grove')
19F-62	0.985029	-0.73363	Ames 27614-7	Utah, USA ('Maple Grove')
19F-63	1.850283	-1.73375	Ames 27614-8	Utah, USA ('Maple Grove')
19F-64	1.376511	-1.61489	Ames 27614-10	Utah, USA ('Maple Grove')
19F-66	1.608939	-1.719	Ames 27614-12	Utah, USA ('Maple Grove')
19F-67	1.729611	-1.72638	Ames 27614-13	Utah, USA ('Maple Grove')
19F-68	1.979871	-1.65177	Ames 27614-14	Utah, USA ('Maple Grove')
19F-69	1.729611	-1.72638	Ames 27614-15	Utah, USA ('Maple Grove')
19F-70	1.742057	-1.72681	Ames 27614-16	Utah, USA ('Maple Grove')
19F-71	1.729611	-1.72638	Ames 27614-18	Utah, USA ('Maple Grove')
19F-72	1.496578	-1.71241	Ames 27614-20	Utah, USA ('Maple Grove')
19F-722	1.500508	-1.71265	Ames 31368-1	Nevada, USA
19F-723	1.733672	-1.72658	Ames 31368-2	Nevada, USA
19F-724	1.733672	-1.72658	Ames 31368-4	Nevada, USA
19F-725	1.745869	-1.72831	Ames 31368-5	Nevada, USA
19F-726	1.613	-1.71921	Ames 31368-6	Nevada, USA
19F-727	1.970955	-1.74113	Ames 31368-7	Nevada, USA
19F-728	1.970955	-1.74113	Ames 31368-10	Nevada, USA
19F-729	1.492328	-1.71183	Ames 31368-13	Nevada, USA
19F-730	1.858463	-1.73457	Ames 31368-14	Nevada, USA
19F-731	1.850283	-1.73375	Ames 31368-16	Nevada, USA
19F-732	1.384102	-1.70488	Ames 31368-17	Nevada, USA

19F-733	1.737791	-1.7272	Ames 31368-19	Nevada, USA
19F-76	0.833216	-1.10477	Ames 31360-5	Arizona, USA
19F-79	0.861653	-0.80411	Ames 31360-11	Arizona, USA
19F-80	1.318938	-1.14775	Ames 31360-15	Arizona, USA
19F-81	1.529265	-1.41233	Ames 31360-16	Arizona, USA
19F-82	0.768083	-0.72802	Ames 31360-18	Arizona, USA
19F-904	2.021916	-1.28005	Ames 31364-3	Colorado, USA
19F-905	2.021916	-1.28005	Ames 31364-4	Colorado, USA
19F-906	1.737791	-1.7272	Ames 31364-5	Colorado, USA
19F-907	1.911372	-1.17632	Ames 31364-7	Colorado, USA
19F-908	1.729611	-1.72638	Ames 31364-8	Colorado, USA
19F-909	1.556698	-1.24548	Ames 31364-11	Colorado, USA
19F-910	1.970955	-1.74113	Ames 31364-15	Colorado, USA
19F-911	1.858463	-1.73457	Ames 31364-16	Colorado, USA
19F-1577	-2.00416	1.252296	Ames 31364-17	Wyoming, USA
19F-58	-1.48086	-1.43693	Ames 27614-3	Utah, USA
19F-75	-1.30143	-0.98235	Ames 31360-4	Arizona, USA
19F-73	-0.76325	-1.57664	Ames 31360-1	Arizona, USA
19F-77	-0.17605	-0.58274	Ames 31360-6	Arizona, USA

Table S2 – Sheet 4: *Linum perenne* nonhybrids

id	X	Y		sort for positive Ys for the top pure taxon cluster (yellow); all negatives would be in the lower cluster
19F-1000	31.52255	0.977164	CN 107283-20	Hungary, wild material
19F-1518	25.8963	-1.08874	PI 650323-1	Hungary, wild material
19F-1519	22.849	-1.35255	PI 650323-2	Hungary, wild material
19F-1520	24.85907	-0.73475	PI 650323-3	Hungary, wild material
19F-1521	24.27273	-1.66341	PI 650323-4	Hungary, wild material
19F-1522	24.80394	-1.62552	PI 650323-5	Hungary, wild material
19F-1523	23.04484	-2.32478	PI 650323-6	Hungary, wild material
19F-1524	19.77988	-0.12764	PI 650323-7	Hungary, wild material
19F-1525	23.38564	-1.67544	PI 650323-8	Hungary, wild material
19F-1526	23.85059	-0.60822	PI 650323-9	Hungary, wild material
19F-1527	25.26849	-1.90629	PI 650323-10	Hungary, wild material
19F-1528	24.11788	-1.54242	PI 650323-11	Hungary, wild material
19F-1529	25.98317	-1.89474	PI 650323-12	Hungary, wild material
19F-1530	25.26169	-1.42593	PI 650323-13	Hungary, wild material
19F-1531	24.10427	-1.16221	PI 650323-14	Hungary, wild material

19F-1532	23.56066	-1.27549	PI 650323-15	Hungary, wild material
19F-1533	23.3909	-1.91098	PI 650323-16	Hungary, wild material
19F-1534	24.49112	-2.37999	PI 650323-17	Hungary, wild material
19F-1535	24.03299	-1.82629	PI 650323-18	Hungary, wild material
19F-1536	25.06649	-0.08438	PI 650323-19	Hungary, wild material
19F-1537	24.09152	-0.9364	PI 650323-20	Hungary, wild material
19F-1673	9.9854	-2.34781	CN 19179-1	wild material, origin unknown
19F-1674	13.46116	-1.85637	CN 19179-2	wild material, origin unknown
19F-1675	12.15641	-2.45629	CN 19179-3	wild material, origin unknown
19F-1676	13.48068	-2.07815	CN 19179-5	wild material, origin unknown
19F-1677	13.43312	-2.7157	CN 19179-8	wild material, origin unknown
19F-1678	29.00762	1.269076	CN 107282-1	origin unknown
19F-1679	26.3307	1.099167	CN 107282-2	origin unknown
19F-1680	30.63805	0.853683	CN 107282-5	origin unknown
19F-535	29.2475	2.021721	PI 650324-1	Hungary
19F-536	28.9606	0.764095	PI 650324-2	Hungary
19F-537	28.94712	0.783398	PI 650324-3	Hungary
19F-538	29.37836	1.909507	PI 650324-4	Hungary
19F-539	31.1627	0.763316	PI 650324-5	Hungary
19F-540	28.99489	0.246097	PI 650324-6	Hungary

19F-541	27.38398	-0.09433	PI 650324-7	Hungary
19F-542	30.8368	0.870235	PI 650324-8	Hungary
19F-543	29.26651	0.4494	PI 650324-9	Hungary
19F-544	29.18701	0.96306	PI 650324-10	Hungary
19F-545	29.43242	1.06201	PI 650324-11	Hungary
19F-546	29.83394	0.119839	PI 650324-13	Hungary
19F-547	30.43173	1.982679	PI 650324-14	Hungary
19F-548	31.28996	0.852843	PI 650324-15	Hungary
19F-549	30.59815	0.491098	PI 650324-16	Hungary
19F-550	30.74086	0.865954	PI 650324-17	Hungary
19F-551	29.15057	0.605928	PI 650324-18	Hungary
19F-552	30.58386	1.298252	PI 650324-19	Hungary
19F-734	30.08331	0.605783	PI 650325-1	Orgovany, Hungary
19F-735	29.39365	0.740221	PI 650325-2	Orgovany, Hungary
19F-736	30.16705	0.582883	PI 650325-3	Orgovany, Hungary
19F-737	29.44704	0.84518	PI 650325-4	Orgovany, Hungary
19F-738	30.14545	1.172644	PI 650325-5	Orgovany, Hungary
19F-739	31.03583	1.242654	PI 650325-6	Orgovany, Hungary
19F-740	29.35899	0.190061	PI 650325-7	Orgovany, Hungary
19F-741	28.70332	0.083346	PI 650325-8	Orgovany, Hungary
19F-742	31.01578	1.766125	PI 650325-9	Orgovany, Hungary
19F-743	29.60023	0.268039	PI 650325-10	Orgovany, Hungary
19F-744	29.36192	0.687409	PI 650325-11	Orgovany, Hungary
19F-745	28.90013	0.723603	PI 650325-12	Orgovany, Hungary
19F-746	28.81811	-0.26537	PI 650325-13	Orgovany, Hungary
19F-747	28.94249	1.451356	PI 650325-14	Orgovany, Hungary
19F-748	28.96412	0.726783	PI 650325-15	Orgovany, Hungary
19F-749	30.2461	0.951718	PI 650325-16	Orgovany, Hungary
19F-750	30.50875	1.58073	PI 650325-17	Orgovany, Hungary
19F-751	28.55376	0.768646	PI 650325-18	Orgovany, Hungary
19F-752	30.13975	0.936483	PI 650325-19	Orgovany, Hungary

19F-753	28.69913	0.7531	PI 650325-20	Orgovany, Hungary
19F-982	28.89947	0.871391	CN 107283-1	Hungary, wild material
19F-983	30.43909	1.42507	CN 107283-2	Hungary, wild material
19F-984	30.5791	0.885213	CN 107283-4	Hungary, wild material
19F-985	29.53939	0.55079	CN 107283-5	Hungary, wild material
19F-986	30.87382	1.433049	CN 107283-6	Hungary, wild material
19F-987	30.87111	0.910323	CN 107283-7	Hungary, wild material
19F-988	30.97803	1.073189	CN 107283-8	Hungary, wild material
19F-989	31.46649	1.376266	CN 107283-9	Hungary, wild material
19F-991	31.70029	1.038898	CN 107283-11	Hungary, wild material
19F-992	29.19852	0.74228	CN 107283-12	Hungary, wild material
19F-993	30.43574	1.53033	CN 107283-13	Hungary, wild material
19F-994	31.4378	0.820805	CN 107283-14	Hungary, wild material
19F-995	31.78889	0.895302	CN 107283-15	Hungary, wild material
19F-996	31.44082	0.995287	CN 107283-16	Hungary, wild material
19F-997	30.49596	0.628406	CN 107283-17	Hungary, wild material
19F-998	31.71519	0.578498	CN 107283-18	Hungary, wild material
19F-999	32.0172	1.317852	CN 107283-19	Hungary, wild material

Table S2 – Sheet 5: Reference sheet, all genotypes

Identification code for analyses	Species	GRIN
19F-251	*Linum austriacum*	PI 650297-11
19F-252	*Linum austriacum*	PI 650297-11
19F-253	*Linum austriacum*	PI 650297-13
19F-254	*Linum austriacum*	PI 650297-14
19F-255	*Linum austriacum*	PI 650297-15
19F-256	*Linum austriacum*	PI 650297-16
19F-257	*Linum austriacum*	PI 650297-17
19F-258	*Linum austriacum*	PI 650297-18
19F-259	*Linum austriacum*	PI 650297-19
19F-260	*Linum austriacum*	PI 650297-20
19F-771	*Linum austriacum*	PI 650298-1
19F-772	*Linum austriacum*	PI 650298-6
19F-773	*Linum austriacum*	PI 650298-7
19F-774	*Linum austriacum*	PI 650298-8
19F-775	*Linum austriacum*	PI 650298-9
19F-776	*Linum austriacum*	PI 650298-10
19F-777	*Linum austriacum*	PI 650298-11
19F-778	*Linum austriacum*	PI 650298-12
19F-779	*Linum austriacum*	PI 650298-13
19F-780	*Linum austriacum*	PI 650298-14
19F-781	*Linum austriacum*	PI 650298-15
19F-782	*Linum austriacum*	PI 650298-16
19F-783	*Linum austriacum*	PI 650298-17
19F-784	*Linum austriacum*	PI 650298-18
19F-785	*Linum austriacum*	PI 650298-19
19F-786	*Linum austriacum*	PI 650298-20

19F-787	*Linum austriacum*	PI 650298-21
19F-788	*Linum austriacum*	PI 650298-22
19F-1018	*Linum austriacum*	CN 107255-1
19F-1019	*Linum austriacum*	CN 107255-2
19F-1020	*Linum austriacum*	CN 107255-3
19F-1021	*Linum austriacum*	CN 107255-4
19F-1022	*Linum austriacum*	CN 107255-5
19F-1023	*Linum austriacum*	CN 107255-6
19F-1024	*Linum austriacum*	CN 107255-7
19F-1025	*Linum austriacum*	CN 107255-8
19F-1026	*Linum austriacum*	CN 107255-9
19F-1027	*Linum austriacum*	CN 107255-10
19F-1028	*Linum austriacum*	CN 107255-11
19F-1029	*Linum austriacum*	CN 107255-12
19F-1030	*Linum austriacum*	CN 107255-13
19F-1031	*Linum austriacum*	CN 107255-14
19F-1032	*Linum austriacum*	CN 107255-15
19F-1033	*Linum austriacum*	CN 107255-17
19F-1034	*Linum austriacum*	CN 107255-18
19F-1035	*Linum austriacum*	CN 107255-19
19F-1036	*Linum austriacum*	CN 107255-20
19F-1661	*Linum austriacum*	CN 107288-1
19F-1662	*Linum austriacum*	CN 107288-2
19F-1663	*Linum austriacum*	CN 107288-3
19F-1664	*Linum austriacum*	CN 107288-5
19F-1665	*Linum austriacum*	CN 107288-6
19F-1666	*Linum austriacum*	CN 107288-8
19F-1667	*Linum austriacum*	CN 107288-9
19F-1668	*Linum austriacum*	CN 107288-10

19F-1669	*Linum austriacum*	CN 107288-11
19F-1670	*Linum austriacum*	CN 107288-12
19F-1671	*Linum austriacum*	CN 107288-13
19F-1672	*Linum austriacum*	CN 107288-14
19F-1707	*Linum austriacum*	PI 440472-1
19F-1708	*Linum austriacum*	PI 440472-5
19F-1709	*Linum austriacum*	PI 440472-6
19F-1710	*Linum austriacum*	PI 440472-7
19F-1711	*Linum austriacum*	PI 440472-9
19F-1712	*Linum austriacum*	PI 440472-10
19F-1713	*Linum austriacum*	PI 440472-11
19F-1714	*Linum austriacum*	PI 440472-12
19F-1715	*Linum austriacum*	PI 440472-13
19F-1716	*Linum austriacum*	PI 440472-14
19F-1717	*Linum austriacum*	PI 440472-16
19F-1718	*Linum austriacum*	PI 440472-17
19F-1769	*Linum austriacum*	CN 107288-1
19F-1770	*Linum austriacum*	CN 107288-2
19F-1771	*Linum austriacum*	CN 107288-3
19F-1772	*Linum austriacum*	CN 107288-4
19F-1773	*Linum austriacum*	CN 107288-5
19F-1774	*Linum austriacum*	CN 107288-6
19F-1775	*Linum austriacum*	CN 107268-1
19F-1776	*Linum austriacum*	CN 107268-2
19F-1777	*Linum austriacum*	CN 107268-3
19F-1	*Linum austriacum*	PI 502405-1
19F-2	*Linum austriacum*	PI 502405-2
19F-3	*Linum austriacum*	PI 502405-3
19F-4	*Linum austriacum*	PI 502405-4
19F-5	*Linum austriacum*	PI 502405-5
19F-6	*Linum austriacum*	PI 502405-6

19F-7	*Linum austriacum*	PI 502405-7
19F-8	*Linum austriacum*	PI 502405-9
19F-9	*Linum austriacum*	PI 502405-10
19F-10	*Linum austriacum*	PI 502405-11
19F-11	*Linum austriacum*	PI 502405-12
19F-12	*Linum austriacum*	PI 502405-13
19F-14	*Linum austriacum*	PI 502405-16
19F-15	*Linum austriacum*	PI 502405-17
19F-16	*Linum austriacum*	PI 502405-19
19F-17	*Linum austriacum*	PI 502405-20
19F-18	*Linum austriacum*	PI 502410-1
19F-19	*Linum austriacum*	PI 502410-2
19F-20	*Linum austriacum*	PI 502410-3
19F-21	*Linum austriacum*	PI 502410-4
19F-22	*Linum austriacum*	PI 502410-5
19F-23	*Linum austriacum*	PI 502410-6
19F-24	*Linum austriacum*	PI 502410-7
19F-25	*Linum austriacum*	PI 502410-8
19F-26	*Linum austriacum*	PI 502410-9
19F-27	*Linum austriacum*	PI 502410-10
19F-28	*Linum austriacum*	PI 502410-11
19F-29	*Linum austriacum*	PI 502410-12
19F-31	*Linum austriacum*	PI 502410-14
19F-32	*Linum austriacum*	PI 502410-15
19F-33	*Linum austriacum*	PI 502410-18
19F-34	*Linum austriacum*	PI 502410-19
19F-35	*Linum austriacum*	PI 502410-20
19F-36	*Linum austriacum*	PI 650293-1
19F-37	*Linum austriacum*	PI 650293-2
19F-38	*Linum austriacum*	PI 650293-3
19F-39	*Linum austriacum*	PI 650293-4
19F-41	*Linum austriacum*	PI 650293-6
19F-42	*Linum austriacum*	PI 650293-7
19F-43	*Linum austriacum*	PI 650293-8
19F-44	*Linum austriacum*	PI 650293-9
19F-45	*Linum austriacum*	PI 650293-10

19F-46	*Linum austriacum*	PI 650293-11
19F-47	*Linum austriacum*	PI 650293-12
19F-48	*Linum austriacum*	PI 650293-13
19F-49	*Linum austriacum*	PI 650293-14
19F-50	*Linum austriacum*	PI 650293-15
19F-51	*Linum austriacum*	PI 650293-16
19F-52	*Linum austriacum*	PI 650293-17
19F-53	*Linum austriacum*	PI 650293-18
19F-54	*Linum austriacum*	PI 650293-19
19F-55	*Linum austriacum*	PI 650293-20
19F-96	*Linum austriacum*	PI 650301-1
19F-97	*Linum austriacum*	PI 650301-4
19F-98	*Linum austriacum*	PI 650301-6
19F-99	*Linum austriacum*	PI 650301-7
19F-100	*Linum austriacum*	PI 650301-8
19F-101	*Linum austriacum*	PI 650301-10
19F-102	*Linum austriacum*	PI 650301-13
19F-103	*Linum austriacum*	PI 650301-14
19F-104	*Linum austriacum*	PI 650301-15
19F-106	*Linum austriacum*	PI 650301-18
19F-107	*Linum austriacum*	PI 650301-19
19F-108	*Linum austriacum*	PI 650301-20
19F-123	*Linum austriacum*	Ames 29749-1
19F-125	*Linum austriacum*	Ames 29749-3
19F-126	*Linum austriacum*	Ames 29749-4
19F-127	*Linum austriacum*	Ames 29749-5
19F-128	*Linum austriacum*	Ames 29749-7
19F-129	*Linum austriacum*	Ames 29749-8
19F-130	*Linum austriacum*	Ames 29749-9
19F-131	*Linum austriacum*	Ames 29749-10
19F-132	*Linum austriacum*	Ames 29749-11
19F-133	*Linum austriacum*	Ames 29749-12
19F-134	*Linum austriacum*	Ames 29749-14
19F-135	*Linum austriacum*	Ames 29749-15
19F-136	*Linum austriacum*	Ames 29749-16
19F-137	*Linum austriacum*	Ames 29749-17

19F-138	*Linum austriacum*	Ames 29749-19
19F-139	*Linum austriacum*	PI 440473-1
19F-140	*Linum austriacum*	PI 440473-2
19F-141	*Linum austriacum*	PI 440473-3
19F-142	*Linum austriacum*	PI 440473-6
19F-143	*Linum austriacum*	PI 440473-7
19F-144	*Linum austriacum*	PI 440473-9
19F-145	*Linum austriacum*	PI 440473-10
19F-146	*Linum austriacum*	PI 440473-11
19F-147	*Linum austriacum*	PI 440473-13
19F-148	*Linum austriacum*	PI 440473-14
19F-149	*Linum austriacum*	PI 440473-16
19F-150	*Linum austriacum*	PI 440473-17
19F-151	*Linum austriacum*	PI 440473-18
19F-152	*Linum austriacum*	PI 440473-19
19F-153	*Linum austriacum*	PI 440473-20
19F-160	*Linum austriacum*	PI 650294-1
19F-161	*Linum austriacum*	PI 650294-2
19F-162	*Linum austriacum*	PI 650294-3
19F-163	*Linum austriacum*	PI 650294-4
19F-164	*Linum austriacum*	PI 650294-5
19F-165	*Linum austriacum*	PI 650294-6
19F-166	*Linum austriacum*	PI 650294-7
19F-167	*Linum austriacum*	PI 650294-8
19F-168	*Linum austriacum*	PI 650294-9
19F-169	*Linum austriacum*	PI 650294-10
19F-170	*Linum austriacum*	PI 650294-12
19F-171	*Linum austriacum*	PI 650294-13
19F-172	*Linum austriacum*	PI 650294-14
19F-173	*Linum austriacum*	PI 650294-15
19F-174	*Linum austriacum*	PI 650294-16
19F-175	*Linum austriacum*	PI 650294-17
19F-176	*Linum austriacum*	PI 650294-18
19F-177	*Linum austriacum*	PI 650294-19
19F-178	*Linum austriacum*	PI 650294-20
19F-179	*Linum austriacum*	PI 650299-1

19F-180	*Linum austriacum*	PI 650299-4
19F-181	*Linum austriacum*	PI 650299-6
19F-182	*Linum austriacum*	PI 650299-8
19F-183	*Linum austriacum*	PI 650299-10
19F-184	*Linum austriacum*	PI 650299-12
19F-185	*Linum austriacum*	PI 650299-14
19F-186	*Linum austriacum*	PI 650299-15
19F-187	*Linum austriacum*	PI 650299-16
19F-188	*Linum austriacum*	PI 650299-17
19F-189	*Linum austriacum*	PI 650299-18
19F-190	*Linum austriacum*	PI 650299-19
19F-191	*Linum austriacum*	PI 650300-2
19F-192	*Linum austriacum*	PI 650300-4
19F-193	*Linum austriacum*	PI 650300-5
19F-194	*Linum austriacum*	PI 650300-6
19F-195	*Linum austriacum*	PI 650300-8
19F-196	*Linum austriacum*	PI 650300-9
19F-197	*Linum austriacum*	PI 650300-11
19F-198	*Linum austriacum*	PI 650300-12
19F-199	*Linum austriacum*	PI 650300-13
19F-200	*Linum austriacum*	PI 650300-14
19F-201	*Linum austriacum*	PI 650300-15
19F-202	*Linum austriacum*	PI 650300-16
19F-203	*Linum austriacum*	PI 650300-17
19F-204	*Linum austriacum*	PI 650300-18
19F-205	*Linum austriacum*	PI 650300-19
19F-206	*Linum austriacum*	PI 650300-20
19F-207	*Linum austriacum*	PI 650295-2
19F-208	*Linum austriacum*	PI 650295-4
19F-209	*Linum austriacum*	PI 650295-6
19F-210	*Linum austriacum*	PI 650295-7
19F-211	*Linum austriacum*	PI 650295-8
19F-212	*Linum austriacum*	PI 650295-9
19F-213	*Linum austriacum*	PI 650295-10
19F-214	*Linum austriacum*	PI 650295-11
19F-215	*Linum austriacum*	PI 650295-12

19F-216	*Linum austriacum*	PI 650295-13
19F-217	*Linum austriacum*	PI 650295-14
19F-218	*Linum austriacum*	PI 650295-15
19F-219	*Linum austriacum*	PI 650295-16
19F-220	*Linum austriacum*	PI 650295-17
19F-221	*Linum austriacum*	PI 650295-18
19F-222	*Linum austriacum*	PI 650295-19
19F-223	*Linum austriacum*	PI 650295-20
19F-224	*Linum austriacum*	PI 650296-1
19F-225	*Linum austriacum*	PI 650296-2
19F-226	*Linum austriacum*	PI 650296-3
19F-228	*Linum austriacum*	PI 650296-5
19F-229	*Linum austriacum*	PI 650296-6
19F-230	*Linum austriacum*	PI 650296-7
19F-231	*Linum austriacum*	PI 650296-9
19F-232	*Linum austriacum*	PI 650296-10
19F-233	*Linum austriacum*	PI 650296-11
19F-234	*Linum austriacum*	PI 650296-12
19F-235	*Linum austriacum*	PI 650296-13
19F-236	*Linum austriacum*	PI 650296-14
19F-237	*Linum austriacum*	PI 650296-15
19F-238	*Linum austriacum*	PI 650296-16
19F-239	*Linum austriacum*	PI 650296-17
19F-240	*Linum austriacum*	PI 650296-18
19F-241	*Linum austriacum*	PI 650297-1
19F-242	*Linum austriacum*	PI 650297-2
19F-243	*Linum austriacum*	PI 650297-3
19F-244	*Linum austriacum*	PI 650297-4
19F-245	*Linum austriacum*	PI 650297-5
19F-246	*Linum austriacum*	PI 650297-6
19F-247	*Linum austriacum*	PI 650297-7
19F-248	*Linum austriacum*	PI 650297-8
19F-249	*Linum austriacum*	PI 650297-9
19F-250	*Linum austriacum*	PI 650297-10
19F-56	*Linum lewisii*	Ames 27614-1
19F-57	*Linum lewisii*	Ames 27614-2

19F-58	*Linum lewisii*	Ames 27614-3
19F-59	*Linum lewisii*	Ames 27614-4
19F-60	*Linum lewisii*	Ames 27614-5
19F-61	*Linum lewisii*	Ames 27614-6
19F-62	*Linum lewisii*	Ames 27614-7
19F-63	*Linum lewisii*	Ames 27614-8
19F-64	*Linum lewisii*	Ames 27614-10
19F-66	*Linum lewisii*	Ames 27614-12
19F-67	*Linum lewisii*	Ames 27614-13
19F-68	*Linum lewisii*	Ames 27614-14
19F-69	*Linum lewisii*	Ames 27614-15
19F-70	*Linum lewisii*	Ames 27614-16
19F-71	*Linum lewisii*	Ames 27614-18
19F-72	*Linum lewisii*	Ames 27614-20
19F-73	*Linum lewisii*	Ames 31360-1
19F-75	*Linum lewisii*	Ames 31360-4
19F-76	*Linum lewisii*	Ames 31360-5
19F-77	*Linum lewisii*	Ames 31360-6
19F-78	*Linum lewisii*	Ames 31360-8
19F-79	*Linum lewisii*	Ames 31360-11
19F-80	*Linum lewisii*	Ames 31360-15
19F-81	*Linum lewisii*	Ames 31360-16
19F-82	*Linum lewisii*	Ames 31360-18
19F-118	*Linum lewisii*	Ames 31361-7
19F-119	*Linum lewisii*	Ames 31361-8
19F-120	*Linum lewisii*	Ames 31361-12
19F-121	*Linum lewisii*	Ames 31361-13
19F-122	*Linum lewisii*	Ames 31361-14
19F-261	*Linum lewisii*	PI 522306-1
19F-262	*Linum lewisii*	PI 522306-2
19F-263	*Linum lewisii*	PI 522306-3
19F-264	*Linum lewisii*	PI 522306-4
19F-266	*Linum lewisii*	PI 522306-6
19F-267	*Linum lewisii*	PI 522306-7
19F-268	*Linum lewisii*	PI 522306-8
19F-269	*Linum lewisii*	PI 522306-9

19F-270	*Linum lewisii*	PI 522306-10
19F-271	*Linum lewisii*	PI 522306-11
19F-272	*Linum lewisii*	PI 522306-12
19F-273	*Linum lewisii*	PI 522306-13
19F-274	*Linum lewisii*	PI 522306-14
19F-275	*Linum lewisii*	PI 522306-15
19F-276	*Linum lewisii*	PI 522306-17
19F-277	*Linum lewisii*	PI 522306-19
19F-278	*Linum lewisii*	PI 522306-20
19F-279	*Linum lewisii*	PI 650320-1
19F-280	*Linum lewisii*	PI 650320-2
19F-281	*Linum lewisii*	PI 650320-3
19F-282	*Linum lewisii*	PI 650320-4
19F-283	*Linum lewisii*	PI 650320-5
19F-284	*Linum lewisii*	PI 650320-6
19F-285	*Linum lewisii*	PI 650320-8
19F-286	*Linum lewisii*	PI 650320-10
19F-287	*Linum lewisii*	PI 650320-11
19F-288	*Linum lewisii*	PI 650320-12
19F-289	*Linum lewisii*	PI 650320-13
19F-290	*Linum lewisii*	PI 650320-14
19F-291	*Linum lewisii*	PI 650320-15
19F-292	*Linum lewisii*	PI 650320-16
19F-293	*Linum lewisii*	PI 650320-17
19F-294	*Linum lewisii*	PI 650320-19
19F-295	*Linum lewisii*	PI 650320-20
19F-296	*Linum lewisii*	Ames 29912-1
19F-297	*Linum lewisii*	Ames 29912-2
19F-298	*Linum lewisii*	Ames 29912-3
19F-299	*Linum lewisii*	Ames 29912-5
19F-300	*Linum lewisii*	Ames 29912-6
19F-301	*Linum lewisii*	Ames 29912-7
19F-302	*Linum lewisii*	Ames 29912-10
19F-303	*Linum lewisii*	Ames 29912-11
19F-304	*Linum lewisii*	Ames 29912-12
19F-305	*Linum lewisii*	Ames 29912-14

19F-306	*Linum lewisii*	Ames 29912-16
19F-308	*Linum lewisii*	Ames 29912-20
19F-309	*Linum lewisii*	Ames 32568-6
19F-310	*Linum lewisii*	Ames 32568-8
19F-311	*Linum lewisii*	Ames 32568-9
19F-312	*Linum lewisii*	Ames 32568-15
19F-313	*Linum lewisii*	Ames 33352-1
19F-314	*Linum lewisii*	Ames 33352-3
19F-315	*Linum lewisii*	Ames 33352-6
19F-316	*Linum lewisii*	Ames 33352-7
19F-317	*Linum lewisii*	Ames 33352-10
19F-318	*Linum lewisii*	Ames 33352-13
19F-319	*Linum lewisii*	Ames 33352-14
19F-320	*Linum lewisii*	Ames 33352-15
19F-321	*Linum lewisii*	Ames 33352-18
19F-322	*Linum lewisii*	Ames 33352-20
19F-323	*Linum lewisii*	Ames 33353-1
19F-324	*Linum lewisii*	Ames 33353-2
19F-325	*Linum lewisii*	Ames 33353-4
19F-326	*Linum lewisii*	Ames 33353-6
19F-327	*Linum lewisii*	Ames 33353-8
19F-328	*Linum lewisii*	Ames 33353-9
19F-329	*Linum lewisii*	Ames 33353-10
19F-330	*Linum lewisii*	Ames 33353-11
19F-331	*Linum lewisii*	Ames 33353-12
19F-332	*Linum lewisii*	Ames 33353-13
19F-333	*Linum lewisii*	Ames 33353-15
19F-334	*Linum lewisii*	Ames 33353-16
19F-335	*Linum lewisii*	Ames 33353-17
19F-336	*Linum lewisii*	Ames 33353-20
19F-394	*Linum lewisii*	Ames 31366-7
19F-395	*Linum lewisii*	Ames 31366-9
19F-396	*Linum lewisii*	Ames 31366-10
19F-397	*Linum lewisii*	Ames 31366-13
19F-398	*Linum lewisii*	Ames 31366-15
19F-399	*Linum lewisii*	Ames 31366-16

19F-400	*Linum lewisii*	Ames 31366-17
19F-401	*Linum lewisii*	Ames 31369-2
19F-402	*Linum lewisii*	Ames 31369-4
19F-403	*Linum lewisii*	Ames 31369-8
19F-404	*Linum lewisii*	Ames 31369-9
19F-405	*Linum lewisii*	Ames 31369-12
19F-406	*Linum lewisii*	Ames 31369-15
19F-407	*Linum lewisii*	Ames 31361-1
19F-408	*Linum lewisii*	Ames 31361-3
19F-409	*Linum lewisii*	Ames 31361-4
19F-410	*Linum lewisii*	Ames 31361-9
19F-411	*Linum lewisii*	Ames 31361-10
19F-412	*Linum lewisii*	Ames 31361-12
19F-413	*Linum lewisii*	Ames 31361-14
19F-414	*Linum lewisii*	Ames 31361-15
19F-415	*Linum lewisii*	Ames 31361-16
19F-416	*Linum lewisii*	Ames 31361-17
19F-417	*Linum lewisii*	Ames 31361-18
19F-418	*Linum lewisii*	Ames 31361-20
19F-451	*Linum lewisii*	Ames 31365-1
19F-452	*Linum lewisii*	Ames 31365-2
19F-453	*Linum lewisii*	Ames 31365-5
19F-454	*Linum lewisii*	Ames 31365-8
19F-455	*Linum lewisii*	Ames 31365-10
19F-456	*Linum lewisii*	Ames 31365-11
19F-457	*Linum lewisii*	Ames 31365-14
19F-458	*Linum lewisii*	Ames 31365-17
19F-459	*Linum lewisii*	Ames 31365-18
19F-460	*Linum lewisii*	Ames 31370-1
19F-461	*Linum lewisii*	Ames 31370-2
19F-462	*Linum lewisii*	Ames 31370-6
19F-463	*Linum lewisii*	Ames 31370-11
19F-465	*Linum lewisii*	Ames 31370-13
19F-466	*Linum lewisii*	Ames 31370-14
19F-467	*Linum lewisii*	Ames 31370-16
19F-468	*Linum lewisii*	Ames 31370-17

19F-469	*Linum lewisii*	Ames 31370-19
19F-470	*Linum lewisii*	Ames 32565-1
19F-471	*Linum lewisii*	Ames 32565-3
19F-472	*Linum lewisii*	Ames 32565-4
19F-473	*Linum lewisii*	Ames 32565-5
19F-474	*Linum lewisii*	Ames 32565-6
19F-475	*Linum lewisii*	Ames 32565-7
19F-476	*Linum lewisii*	Ames 32565-8
19F-477	*Linum lewisii*	Ames 32565-9
19F-478	*Linum lewisii*	Ames 32565-10
19F-479	*Linum lewisii*	Ames 32565-11
19F-480	*Linum lewisii*	Ames 32565-12
19F-481	*Linum lewisii*	Ames 32565-13
19F-482	*Linum lewisii*	Ames 32565-14
19F-483	*Linum lewisii*	Ames 32565-15
19F-484	*Linum lewisii*	Ames 32565-16
19F-485	*Linum lewisii*	Ames 32565-17
19F-486	*Linum lewisii*	Ames 32565-18
19F-487	*Linum lewisii*	Ames 32565-19
19F-488	*Linum lewisii*	Ames 32565-20
19F-553	*Linum lewisii*	Ames 33354-1
19F-554	*Linum lewisii*	Ames 33354-2
19F-555	*Linum lewisii*	Ames 33354-6
19F-556	*Linum lewisii*	Ames 33354-9
19F-557	*Linum lewisii*	Ames 33354-10
19F-558	*Linum lewisii*	Ames 33354-14
19F-559	*Linum lewisii*	Ames 33354-17
19F-560	*Linum lewisii*	Ames 33354-19
19F-561	*Linum lewisii*	PI 522305-1
19F-562	*Linum lewisii*	PI 522305-2
19F-563	*Linum lewisii*	PI 522305-3
19F-564	*Linum lewisii*	PI 522305-4
19F-565	*Linum lewisii*	PI 522305-6
19F-566	*Linum lewisii*	PI 522305-8
19F-567	*Linum lewisii*	PI 522305-9
19F-568	*Linum lewisii*	PI 522305-12

19F-569	*Linum lewisii*	PI 522305-13
19F-570	*Linum lewisii*	PI 522305-15
19F-571	*Linum lewisii*	PI 522305-16
19F-572	*Linum lewisii*	PI 522305-17
19F-573	*Linum lewisii*	PI 452487-1
19F-574	*Linum lewisii*	PI 452487-2
19F-575	*Linum lewisii*	PI 452487-4
19F-576	*Linum lewisii*	PI 452487-5
19F-577	*Linum lewisii*	PI 452487-6
19F-578	*Linum lewisii*	PI 452487-7
19F-579	*Linum lewisii*	PI 452487-8
19F-580	*Linum lewisii*	PI 452487-9
19F-581	*Linum lewisii*	PI 452487-10
19F-582	*Linum lewisii*	PI 452487-11
19F-583	*Linum lewisii*	PI 452487-13
19F-584	*Linum lewisii*	PI 452487-17
19F-722	*Linum lewisii*	Ames 31368-1
19F-723	*Linum lewisii*	Ames 31368-2
19F-724	*Linum lewisii*	Ames 31368-4
19F-725	*Linum lewisii*	Ames 31368-5
19F-726	*Linum lewisii*	Ames 31368-6
19F-727	*Linum lewisii*	Ames 31368-7
19F-728	*Linum lewisii*	Ames 31368-10
19F-729	*Linum lewisii*	Ames 31368-13
19F-730	*Linum lewisii*	Ames 31368-14
19F-731	*Linum lewisii*	Ames 31368-16
19F-732	*Linum lewisii*	Ames 31368-17
19F-733	*Linum lewisii*	Ames 31368-19
19F-904	*Linum lewisii*	Ames 31364-3
19F-905	*Linum lewisii*	Ames 31364-4
19F-906	*Linum lewisii*	Ames 31364-5
19F-907	*Linum lewisii*	Ames 31364-7
19F-908	*Linum lewisii*	Ames 31364-8
19F-909	*Linum lewisii*	Ames 31364-11
19F-910	*Linum lewisii*	Ames 31364-15
19F-911	*Linum lewisii*	Ames 31364-16

19F-1061	*Linum lewisii*	CN 107266-1
19F-1062	*Linum lewisii*	CN 107266-2
19F-1063	*Linum lewisii*	CN 107266-3
19F-1064	*Linum lewisii*	CN 107266-4
19F-1065	*Linum lewisii*	CN 107266-5
19F-1066	*Linum lewisii*	CN 107266-6
19F-1067	*Linum lewisii*	CN 107266-7
19F-1068	*Linum lewisii*	CN 107266-8
19F-1069	*Linum lewisii*	CN 107266-9
19F-1070	*Linum lewisii*	CN 107266-10
19F-1071	*Linum lewisii*	CN 107266-11
19F-1072	*Linum lewisii*	CN 107266-12
19F-1563	*Linum lewisii*	Ames 31371-1
19F-1564	*Linum lewisii*	Ames 31371-2
19F-1565	*Linum lewisii*	Ames 31371-4
19F-1566	*Linum lewisii*	Ames 31371-6
19F-1567	*Linum lewisii*	Ames 31371-8
19F-1568	*Linum lewisii*	Ames 31371-9
19F-1569	*Linum lewisii*	Ames 31371-10
19F-1570	*Linum lewisii*	Ames 31371-14
19F-1571	*Linum lewisii*	Ames 31371-15
19F-1572	*Linum lewisii*	Ames 31372-1
19F-1573	*Linum lewisii*	Ames 31372-7
19F-1574	*Linum lewisii*	Ames 31372-10
19F-1575	*Linum lewisii*	Ames 31372-11
19F-1576	*Linum lewisii*	Ames 32565-1
19F-1577	*Linum lewisii*	Ames 32565-3
19F-1578	*Linum lewisii*	Ames 32565-4
19F-1579	*Linum lewisii*	Ames 32565-5
19F-1580	*Linum lewisii*	Ames 32565-6
19F-1581	*Linum lewisii*	Ames 32565-7
19F-1582	*Linum lewisii*	Ames 32565-8
19F-1583	*Linum lewisii*	Ames 32565-9
19F-1584	*Linum lewisii*	Ames 32565-10
19F-1585	*Linum lewisii*	Ames 32565-11
19F-1586	*Linum lewisii*	Ames 32565-12

19F-1587	*Linum lewisii*	Ames 32565-13
19F-1588	*Linum lewisii*	Ames 32565-14
19F-1589	*Linum lewisii*	Ames 32565-15
19F-1590	*Linum lewisii*	Ames 32565-16
19F-1591	*Linum lewisii*	Ames 32565-17
19F-1592	*Linum lewisii*	Ames 32565-18
19F-1593	*Linum lewisii*	Ames 32565-19
19F-1594	*Linum lewisii*	Ames 32565-20
19F-1595	*Linum lewisii*	Ames 32566-1
19F-1596	*Linum lewisii*	Ames 32566-2
19F-1597	*Linum lewisii*	Ames 32566-3
19F-1598	*Linum lewisii*	Ames 32566-4
19F-1599	*Linum lewisii*	Ames 32566-6
19F-1600	*Linum lewisii*	Ames 32566-7
19F-1601	*Linum lewisii*	Ames 32566-8
19F-1602	*Linum lewisii*	Ames 32566-9
19F-1603	*Linum lewisii*	Ames 32566-10
19F-1604	*Linum lewisii*	Ames 32566-11
19F-1605	*Linum lewisii*	Ames 32566-14
19F-1606	*Linum lewisii*	Ames 32566-16
19F-1625	*Linum lewisii*	CN 107300-2
19F-1626	*Linum lewisii*	CN 107300-4
19F-1794	*Linum perenne*	CN 107256-3
19F-1795	*Linum perenne*	CN 107256-4
19F-1796	*Linum perenne*	CN 107256-5
19F-1797	*Linum perenne*	CN 107256-6
19F-1798	*Linum perenne*	CN 107256-7
19F-1799	*Linum perenne*	CN 107256-8
19F-1800	*Linum perenne*	CN 107256-10
19F-1801	*Linum perenne*	CN 107256-11
19F-1802	*Linum perenne*	CN 107256-12
19F-1803	*Linum perenne*	CN 107256-13
19F-1804	*Linum perenne*	CN 107256-14
19F-1805	*Linum perenne*	CN 107256-15
19F-1806	*Linum perenne*	CN 107256-16
19F-1807	*Linum perenne*	CN 107256-17

19F-1808	*Linum perenne*	CN 107256-18
19F-1809	*Linum perenne*	CN 107270-1
19F-1810	*Linum perenne*	CN 107270-2
19F-1811	*Linum perenne*	CN 107270-4
19F-1812	*Linum perenne*	CN 107270-5
19F-1813	*Linum perenne*	CN 107270-6
19F-1814	*Linum perenne*	CN 107270-7
19F-1815	*Linum perenne*	CN 107270-8
19F-1816	*Linum perenne*	CN 107270-9
19F-1817	*Linum perenne*	CN 107270-10
19F-1818	*Linum perenne*	CN 107270-11
19F-1819	*Linum perenne*	CN 107270-13
19F-1820	*Linum perenne*	CN 107270-14
19F-1821	*Linum perenne*	CN 107270-15
19F-1822	*Linum perenne*	CN 107270-16
19F-1823	*Linum perenne*	CN 107270-17
19F-1824	*Linum perenne*	CN 107270-18
19F-1825	*Linum perenne*	CN 107270-19
19F-1826	*Linum perenne*	CN 107270-20
19F-337	*Linum perenne*	PI 650327-1
19F-338	*Linum perenne*	PI 650327-2
19F-339	*Linum perenne*	PI 650327-3
19F-340	*Linum perenne*	PI 650327-4
19F-341	*Linum perenne*	PI 650327-6
19F-342	*Linum perenne*	PI 650327-7
19F-343	*Linum perenne*	PI 650327-8
19F-344	*Linum perenne*	PI 650327-9
19F-345	*Linum perenne*	PI 650327-10
19F-346	*Linum perenne*	PI 650327-11
19F-347	*Linum perenne*	PI 650327-12
19F-348	*Linum perenne*	PI 650327-13
19F-349	*Linum perenne*	PI 650327-14
19F-350	*Linum perenne*	PI 650327-15
19F-351	*Linum perenne*	PI 650327-16
19F-352	*Linum perenne*	PI 650327-17
19F-353	*Linum perenne*	PI 650327-18

19F-354	*Linum perenne*	PI 650327-19
19F-355	*Linum perenne*	PI 650327-20
19F-356	*Linum perenne*	PI 650328-1
19F-357	*Linum perenne*	PI 650328-2
19F-358	*Linum perenne*	PI 650328-3
19F-359	*Linum perenne*	PI 650328-4
19F-360	*Linum perenne*	PI 650328-6
19F-361	*Linum perenne*	PI 650328-7
19F-362	*Linum perenne*	PI 650328-8
19F-363	*Linum perenne*	PI 650328-9
19F-364	*Linum perenne*	PI 650328-10
19F-365	*Linum perenne*	PI 650328-11
19F-366	*Linum perenne*	PI 650328-12
19F-367	*Linum perenne*	PI 650328-13
19F-368	*Linum perenne*	PI 650328-14
19F-369	*Linum perenne*	PI 650328-15
19F-370	*Linum perenne*	PI 650328-16
19F-371	*Linum perenne*	PI 650328-17
19F-372	*Linum perenne*	PI 650328-18
19F-373	*Linum perenne*	PI 650328-19
19F-374	*Linum perenne*	PI 650328-20
19F-375	*Linum perenne*	PI 650329-1
19F-376	*Linum perenne*	PI 650329-2
19F-377	*Linum perenne*	PI 650329-3
19F-378	*Linum perenne*	PI 650329-4
19F-379	*Linum perenne*	PI 650329-5
19F-380	*Linum perenne*	PI 650329-6
19F-381	*Linum perenne*	PI 650329-7
19F-382	*Linum perenne*	PI 650329-8
19F-383	*Linum perenne*	PI 650329-9
19F-384	*Linum perenne*	PI 650329-10
19F-385	*Linum perenne*	PI 650329-11
19F-386	*Linum perenne*	PI 650329-12
19F-387	*Linum perenne*	PI 650329-13
19F-388	*Linum perenne*	PI 650329-14
19F-389	*Linum perenne*	PI 650329-15

19F-390	*Linum perenne*	PI 650329-16
19F-391	*Linum perenne*	PI 650329-18
19F-392	*Linum perenne*	PI 650329-19
19F-393	*Linum perenne*	PI 650329-20
19F-499	*Linum perenne*	Ames 21222-1
19F-500	*Linum perenne*	Ames 21222-2
19F-501	*Linum perenne*	Ames 21222-3
19F-502	*Linum perenne*	Ames 21222-4
19F-503	*Linum perenne*	Ames 21222-5
19F-504	*Linum perenne*	Ames 21222-6
19F-505	*Linum perenne*	Ames 21222-8
19F-506	*Linum perenne*	Ames 21222-12
19F-507	*Linum perenne*	Ames 21222-13
19F-508	*Linum perenne*	Ames 21222-14
19F-509	*Linum perenne*	Ames 21222-15
19F-510	*Linum perenne*	Ames 21222-16
19F-511	*Linum perenne*	Ames 21222-17
19F-512	*Linum perenne*	Ames 21222-18
19F-513	*Linum perenne*	Ames 21222-19
19F-514	*Linum perenne*	Ames 21222-20
19F-515	*Linum perenne*	Ames 31374-1
19F-516	*Linum perenne*	Ames 31374-2
19F-517	*Linum perenne*	Ames 31374-3
19F-518	*Linum perenne*	Ames 31374-4
19F-519	*Linum perenne*	Ames 31374-5
19F-520	*Linum perenne*	Ames 31374-6
19F-521	*Linum perenne*	Ames 31374-7
19F-522	*Linum perenne*	Ames 31374-8
19F-523	*Linum perenne*	Ames 31374-9
19F-524	*Linum perenne*	Ames 31374-10
19F-525	*Linum perenne*	Ames 31374-11
19F-526	*Linum perenne*	Ames 31374-12
19F-527	*Linum perenne*	Ames 31374-13
19F-528	*Linum perenne*	Ames 31374-14
19F-529	*Linum perenne*	Ames 31374-15
19F-530	*Linum perenne*	Ames 31374-16

19F-532	*Linum perenne*	Ames 31374-18
19F-533	*Linum perenne*	Ames 31374-19
19F-534	*Linum perenne*	Ames 31374-20
19F-535	*Linum perenne*	PI 650324-1
19F-536	*Linum perenne*	PI 650324-2
19F-537	*Linum perenne*	PI 650324-3
19F-538	*Linum perenne*	PI 650324-4
19F-539	*Linum perenne*	PI 650324-5
19F-540	*Linum perenne*	PI 650324-6
19F-541	*Linum perenne*	PI 650324-7
19F-542	*Linum perenne*	PI 650324-8
19F-543	*Linum perenne*	PI 650324-9
19F-544	*Linum perenne*	PI 650324-10
19F-545	*Linum perenne*	PI 650324-11
19F-546	*Linum perenne*	PI 650324-13
19F-547	*Linum perenne*	PI 650324-14
19F-548	*Linum perenne*	PI 650324-15
19F-549	*Linum perenne*	PI 650324-16
19F-550	*Linum perenne*	PI 650324-17
19F-551	*Linum perenne*	PI 650324-18
19F-552	*Linum perenne*	PI 650324-19
19F-734	*Linum perenne*	PI 650325-1
19F-735	*Linum perenne*	PI 650325-2
19F-736	*Linum perenne*	PI 650325-3
19F-737	*Linum perenne*	PI 650325-4
19F-738	*Linum perenne*	PI 650325-5
19F-739	*Linum perenne*	PI 650325-6
19F-740	*Linum perenne*	PI 650325-7
19F-741	*Linum perenne*	PI 650325-8
19F-742	*Linum perenne*	PI 650325-9
19F-743	*Linum perenne*	PI 650325-10
19F-744	*Linum perenne*	PI 650325-11
19F-745	*Linum perenne*	PI 650325-12
19F-746	*Linum perenne*	PI 650325-13
19F-747	*Linum perenne*	PI 650325-14
19F-748	*Linum perenne*	PI 650325-15

19F-749	*Linum perenne*	PI 650325-16
19F-750	*Linum perenne*	PI 650325-17
19F-751	*Linum perenne*	PI 650325-18
19F-752	*Linum perenne*	PI 650325-19
19F-753	*Linum perenne*	PI 650325-20
19F-754	*Linum perenne*	PI 650326-1
19F-755	*Linum perenne*	PI 650326-2
19F-756	*Linum perenne*	PI 650326-3
19F-757	*Linum perenne*	PI 650326-5
19F-758	*Linum perenne*	PI 650326-6
19F-759	*Linum perenne*	PI 650326-7
19F-760	*Linum perenne*	PI 650326-8
19F-761	*Linum perenne*	PI 650326-10
19F-762	*Linum perenne*	PI 650326-12
19F-763	*Linum perenne*	PI 650326-13
19F-764	*Linum perenne*	PI 650326-14
19F-765	*Linum perenne*	PI 650326-15
19F-766	*Linum perenne*	PI 650326-16
19F-768	*Linum perenne*	PI 650326-18
19F-769	*Linum perenne*	PI 650326-19
19F-770	*Linum perenne*	PI 650326-20
19F-932	*Linum perenne*	Blue Flax-1
19F-933	*Linum perenne*	Blue Flax-2
19F-934	*Linum perenne*	Blue Flax-3
19F-935	*Linum perenne*	Blue Flax-4
19F-936	*Linum perenne*	Blue Flax-5
19F-937	*Linum perenne*	Blue Flax-6
19F-938	*Linum perenne*	Blue Flax-7
19F-939	*Linum perenne*	Blue Flax-8
19F-940	*Linum perenne*	Blue Flax-10
19F-941	*Linum perenne*	Blue Flax-11
19F-942	*Linum perenne*	Blue Flax-12
19F-943	*Linum perenne*	Blue Flax-13
19F-945	*Linum perenne*	Blue Flax-15
19F-946	*Linum perenne*	Blue Flax-16
19F-947	*Linum perenne*	Blue Flax-17

19F-948	*Linum perenne*	Blue Flax-19
19F-949	*Linum perenne*	Blue Flax-20
19F-982	*Linum perenne*	CN 107283-1
19F-983	*Linum perenne*	CN 107283-2
19F-984	*Linum perenne*	CN 107283-4
19F-985	*Linum perenne*	CN 107283-5
19F-986	*Linum perenne*	CN 107283-6
19F-987	*Linum perenne*	CN 107283-7
19F-988	*Linum perenne*	CN 107283-8
19F-989	*Linum perenne*	CN 107283-9
19F-991	*Linum perenne*	CN 107283-11
19F-992	*Linum perenne*	CN 107283-12
19F-993	*Linum perenne*	CN 107283-13
19F-994	*Linum perenne*	CN 107283-14
19F-995	*Linum perenne*	CN 107283-15
19F-996	*Linum perenne*	CN 107283-16
19F-997	*Linum perenne*	CN 107283-17
19F-998	*Linum perenne*	CN 107283-18
19F-999	*Linum perenne*	CN 107283-19
19F-1000	*Linum perenne*	CN 107283-20
19F-1001	*Linum perenne*	PI 445972-1
19F-1002	*Linum perenne*	PI 445972-2
19F-1003	*Linum perenne*	PI 445972-3
19F-1004	*Linum perenne*	PI 445972-4
19F-1005	*Linum perenne*	PI 445972-5
19F-1006	*Linum perenne*	PI 445972-6
19F-1007	*Linum perenne*	PI 445972-7
19F-1008	*Linum perenne*	PI 445972-8
19F-1009	*Linum perenne*	PI 445972-9
19F-1010	*Linum perenne*	PI 445972-10
19F-1011	*Linum perenne*	PI 445972-11
19F-1012	*Linum perenne*	PI 445972-12
19F-1013	*Linum perenne*	PI 445972-13
19F-1014	*Linum perenne*	PI 445972-14
19F-1015	*Linum perenne*	PI 445972-15
19F-1016	*Linum perenne*	PI 445972-16

19F-1017	*Linum perenne*	PI 445972-17
19F-1039	*Linum perenne*	Blue Flax-1
19F-1040	*Linum perenne*	AK wildflowers-2
19F-1041	*Linum perenne*	AK wildflowers-3
19F-1042	*Linum perenne*	AK wildflowers-4
19F-1043	*Linum perenne*	AK wildflowers-5
19F-1044	*Linum perenne*	AK wildflowers-6
19F-1045	*Linum perenne*	AK wildflowers-7
19F-1046	*Linum perenne*	AK wildflowers-8
19F-1047	*Linum perenne*	AK wildflowers-9
19F-1048	*Linum perenne*	AK wildflowers-10
19F-1049	*Linum perenne*	AK wildflowers-11
19F-1050	*Linum perenne*	AK wildflowers-12
19F-1051	*Linum perenne*	AK wildflowers-13
19F-1052	*Linum perenne*	AK wildflowers-14
19F-1053	*Linum perenne*	AK wildflowers-15
19F-1094	*Linum perenne*	PI 445972-2
19F-1095	*Linum perenne*	PI 445972-3
19F-1096	*Linum perenne*	PI 445972-4
19F-1097	*Linum perenne*	PI 445972-5
19F-1098	*Linum perenne*	PI 445972-6
19F-1099	*Linum perenne*	PI 445972-7
19F-1100	*Linum perenne*	PI 445972-8
19F-1101	*Linum perenne*	PI 445972-9
19F-1102	*Linum perenne*	PI 445972-10
19F-1103	*Linum perenne*	PI 445972-11
19F-1104	*Linum perenne*	PI 445972-12
19F-1105	*Linum perenne*	PI 445972-13
19F-1106	*Linum perenne*	PI 445972-14
19F-1107	*Linum perenne*	PI 445972-15
19F-1108	*Linum perenne*	PI 445972-16

19F-1109	*Linum perenne*	PI 445972-18
19F-1110	*Linum perenne*	PI 445972-20
19F-1518	*Linum perenne*	PI 650323-1
19F-1519	*Linum perenne*	PI 650323-2
19F-1520	*Linum perenne*	PI 650323-3
19F-1521	*Linum perenne*	PI 650323-4
19F-1522	*Linum perenne*	PI 650323-5
19F-1523	*Linum perenne*	PI 650323-6
19F-1524	*Linum perenne*	PI 650323-7
19F-1525	*Linum perenne*	PI 650323-8
19F-1526	*Linum perenne*	PI 650323-9
19F-1527	*Linum perenne*	PI 650323-10
19F-1528	*Linum perenne*	PI 650323-11
19F-1529	*Linum perenne*	PI 650323-12
19F-1530	*Linum perenne*	PI 650323-13
19F-1531	*Linum perenne*	PI 650323-14
19F-1532	*Linum perenne*	PI 650323-15
19F-1533	*Linum perenne*	PI 650323-16
19F-1534	*Linum perenne*	PI 650323-17
19F-1535	*Linum perenne*	PI 650323-18
19F-1536	*Linum perenne*	PI 650323-19
19F-1537	*Linum perenne*	PI 650323-20
19F-1647	*Linum perenne*	CN 107261-1
19F-1649	*Linum perenne*	CN 107261-4
19F-1673	*Linum perenne*	CN 19179-1
19F-1674	*Linum perenne*	CN 19179-2
19F-1675	*Linum perenne*	CN 19179-3
19F-1676	*Linum perenne*	CN 19179-5
19F-1677	*Linum perenne*	CN 19179-8
19F-1678	*Linum perenne*	CN 107282-1
19F-1679	*Linum perenne*	CN 107282-2
19F-1680	*Linum perenne*	CN 107282-5
19F-1778	*Linum perenne*	CN 19024-1
19F-1779	*Linum perenne*	CN 19024-2
19F-1780	*Linum perenne*	CN 19024-3
19F-1781	*Linum perenne*	CN 19024-4

19F-1782	*Linum perenne*	CN 19024-5
19F-1783	*Linum perenne*	CN 19024-6
19F-1784	*Linum perenne*	CN 19024-7
19F-1785	*Linum perenne*	CN 19024-8
19F-1786	*Linum perenne*	CN 19024-9
19F-1787	*Linum perenne*	CN 19024-10
19F-1788	*Linum perenne*	CN 19024-11
19F-1789	*Linum perenne*	CN 19024-12
19F-1790	*Linum perenne*	CN 19024-13
19F-1791	*Linum perenne*	CN 19024-14
19F-1792	*Linum perenne*	CN 107256-1
19F-1793	*Linum perenne*	CN 107256-2

Table S2 – Sheet 6: PCoA with all genotypes

id	V1	V2
19F-249	-11.6509	1.720409
19F-1041	-11.6391	1.676815
19F-486	-11.6142	1.599523
19F-946	-11.5772	1.990824
19F-476	-11.5481	1.929326
19F-1015	-11.5098	2.061001
19F-1016	-11.4485	2.365647
19F-1810	-11.4318	2.136634
19F-209	-11.423	1.563572
19F-1784	-11.4166	1.465705
19F-1587	-11.4103	2.255879
19F-1105	-11.3846	2.29294
19F-1042	-11.3775	2.107028
19F-260	-11.373	1.824028
19F-254	-11.3559	1.698633
19F-480	-11.3439	2.320586
19F-1013	-11.3415	1.798814
19F-1588	-11.3402	2.366163
19F-471	-11.3289	2.038991
19F-367	-11.3281	1.512982

19F-364	-11.3173	1.807972
19F-475	-11.3155	2.084573
19F-1050	-11.308	1.798268
19F-1007	-11.305	2.085985
19F-512	-11.2803	1.58661
19F-1800	-11.2339	1.622392
19F-356	-11.2223	1.462528
19F-1043	-11.1977	2.189661
19F-566	-11.1859	2.321362
19F-568	-11.1818	1.822069
19F-1593	-11.1798	1.710004
19F-285	-11.1777	2.658519
19F-1001	-11.1683	1.836085
19F-932	-11.1628	1.970141
19F-278	-11.1509	2.059113
19F-1011	-11.1508	2.014977
19F-570	-11.1456	2.311594
19F-505	-11.1445	1.371165
19F-1582	-11.1299	2.001653
19F-1813	-11.1234	1.56989
19F-506	-11.1203	1.81879
19F-1095	-11.1194	2.122095

19F-1094	-11.1155	1.689645
19F-1585	-11.1021	2.201246
19F-274	-11.1004	2.346552
19F-289	-11.095	2.803384
19F-268	-11.0906	1.643082
19F-366	-11.0892	1.016246
19F-523	-11.0867	1.970025
19F-1580	-11.0691	1.80867
19F-510	-11.065	2.054693
19F-564	-11.0636	2.102085
19F-273	-11.0632	1.824022
19F-935	-11.0575	1.348497
19F-363	-11.0542	2.182912
19F-504	-11.054	2.046293
19F-1049	-11.0524	2.116569
19F-270	-11.0463	2.353739
19F-1794	-11.0462	1.4445
19F-1017	-11.0396	1.995666
19F-572	-11.0391	1.97803
19F-1009	-11.0351	1.892826
19F-1109	-11.033	1.958787
19F-212	-11.031	1.530616
19F-525	-11.0188	1.449617
19F-373	-11.0136	2.510518

19F-243	-11.0127	2.183956
19F-248	-11	1.787108
19F-288	-10.9924	2.431976
19F-563	-10.9909	1.912427
19F-943	-10.9907	1.700754
19F-1823	-10.9863	1.75938
19F-1785	-10.9857	1.848217
19F-503	-10.9824	1.510409
19F-1021	-10.979	1.045495
19F-571	-10.9766	2.082681
19F-1799	-10.9743	1.808199
19F-1048	-10.9737	2.011191
19F-501	-10.9734	1.729389
19F-1052	-10.9641	1.986936
19F-261	-10.9628	1.731109
19F-511	-10.9618	1.43609
19F-474	-10.955	1.605594
19F-1100	-10.9525	1.556569
19F-1780	-10.9466	1.5568
19F-385	-10.9462	2.221516
19F-479	-10.9398	2.552523
19F-521	-10.9394	2.243575
19F-1805	-10.9367	1.999479

19F-293	-10.9281	2.10662
19F-1589	-10.9277	2.48332
19F-295	-10.9246	2.511667
19F-483	-10.9238	1.991352
19F-1811	-10.9215	1.520663
19F-1591	-10.9201	1.613837
19F-283	-10.9059	1.915359
19F-217	-10.9058	1.080702
19F-487	-10.9046	1.794543
19F-1579	-10.8976	2.254425
19F-284	-10.8918	1.909168
19F-391	-10.884	2.193444
19F-1821	-10.8803	1.669258
19F-1039	-10.8796	2.457265
19F-1592	-10.8793	2.008381
19F-362	-10.8791	2.098094
19F-945	-10.8783	1.420099
19F-1096	-10.8756	2.006744
19F-256	-10.8744	1.692536
19F-1791	-10.8732	1.808904
19F-528	-10.8706	1.248405
19F-1817	-10.866	1.516986

19F-376	-10.8613	1.973033
19F-1586	-10.8607	2.082765
19F-933	-10.8591	1.65475
19F-1005	-10.8582	2.017862
19F-1032	-10.8537	1.531857
19F-1053	-10.8497	2.099963
19F-1782	-10.848	1.704555
19F-1010	-10.8446	1.762299
19F-365	-10.8386	1.772976
19F-941	-10.8336	1.917574
19F-1795	-10.8314	1.965148
19F-1826	-10.8254	1.676192
19F-292	-10.8216	2.230659
19F-1108	-10.8159	1.751553
19F-508	-10.8143	1.938504
19F-934	-10.8123	1.801593
19F-1098	-10.8016	2.100849
19F-275	-10.7978	1.721343
19F-522	-10.794	1.745947
19F-280	-10.7936	2.184728
19F-372	-10.7897	1.669833

19F-1779	-10.7863	1.86206
19F-286	-10.7844	2.419013
19F-473	-10.78	1.951673
19F-533	-10.7798	1.545298
19F-947	-10.7787	2.051575
19F-939	-10.7746	2.117576
19F-513	-10.7718	1.581432
19F-567	-10.7713	2.216482
19F-938	-10.7668	1.868349
19F-526	-10.7659	1.628921
19F-258	-10.7657	1.864334
19F-514	-10.7643	2.019116
19F-221	-10.7635	1.439138
19F-940	-10.7617	1.76811
19F-1822	-10.7607	1.760445
19F-1825	-10.7527	1.342148
19F-1003	-10.75	1.725893
19F-562	-10.7486	1.830505
19F-1773	-10.7472	2.008454
19F-291	-10.7419	1.904779
19F-215	-10.7402	0.815305
19F-515	-10.7399	1.74241
19F-1781	-10.731	1.705057
19F-1801	-10.7273	1.680493

19F-1045	-10.7272	1.804819
19F-1106	-10.7255	1.852716
19F-1008	-10.7204	1.706941
19F-1110	-10.7169	2.090549
19F-502	-10.716	1.806243
19F-1778	-10.7125	1.976134
19F-1576	-10.7081	2.185883
19F-949	-10.7002	1.399162
19F-222	-10.6983	1.250681
19F-251	-10.6975	1.722244
19F-371	-10.6972	1.413503
19F-757	-10.6897	1.908498
19F-1014	-10.6887	1.769428
19F-1775	-10.6885	1.172099
19F-208	-10.6787	1.646988
19F-220	-10.6748	1.803536
19F-1818	-10.6705	1.864491
19F-529	-10.6628	1.610993
19F-1797	-10.6623	1.693943
19F-357	-10.6609	1.303624
19F-1820	-10.6592	1.703422

19F-1099	-10.6562	1.660489
19F-481	-10.6541	1.68853
19F-488	-10.6527	1.64524
19F-1051	-10.6522	2.146192
19F-384	-10.6503	2.015456
19F-1028	-10.6499	1.251125
19F-257	-10.6488	1.856533
19F-1819	-10.6394	1.361394
19F-569	-10.6383	1.970765
19F-1101	-10.6379	1.574773
19F-1004	-10.6371	1.912973
19F-517	-10.6315	1.273086
19F-530	-10.6293	1.732183
19F-532	-10.6291	1.242046
19F-1006	-10.6274	1.871836
19F-361	-10.6237	1.274274
19F-520	-10.617	1.675716
19F-1040	-10.6159	2.243628
19F-470	-10.611	2.303607
19F-499	-10.6072	1.859319
19F-386	-10.6027	2.017538
19F-1012	-10.6001	1.709052
19F-213	-10.5999	1.700832

19F-1807	-10.5973	1.324334
19F-255	-10.5935	1.300568
19F-534	-10.5931	1.85884
19F-509	-10.5926	2.288166
19F-368	-10.5845	1.425903
19F-379	-10.5837	2.151758
19F-1594	-10.5794	1.520386
19F-1772	-10.5754	1.21216
19F-290	-10.5729	1.687555
19F-1590	-10.5709	2.103866
19F-370	-10.5689	1.401138
19F-527	-10.5672	1.670759
19F-1649	-10.5653	1.831925
19F-1104	-10.5634	1.375603
19F-484	-10.5626	1.424913
19F-214	-10.5529	0.675214
19F-1018	-10.5489	1.170154
19F-1046	-10.5445	2.058071
19F-1796	-10.5381	1.443266
19F-1583	-10.5326	1.653401
19F-218	-10.532	0.979418

19F-1097	-10.5298	1.638436
19F-1034	-10.5228	0.968609
19F-375	-10.5188	1.592326
19F-519	-10.5093	2.16017
19F-223	-10.4888	0.980554
19F-763	-10.4887	1.760539
19F-948	-10.4664	2.308907
19F-478	-10.4656	1.921149
19F-381	-10.4628	2.095682
19F-766	-10.461	2.145311
19F-1814	-10.4577	1.739946
19F-1002	-10.4577	2.058922
19F-1581	-10.4563	1.737978
19F-1036	-10.4538	0.805164
19F-482	-10.4499	2.026781
19F-393	-10.4499	2.469102
19F-259	-10.4483	2.01326
19F-1064	-10.4477	2.243103
19F-241	-10.4458	1.267866
19F-1020	-10.4399	1.229812
19F-760	-10.4307	1.838449
19F-758	-10.4202	1.723964
19F-477	-10.4175	1.633997

19F-1107	-10.4158	1.841595
19F-252	-10.4126	1.737933
19F-1786	-10.4013	1.712015
19F-1025	-10.3909	1.315315
19F-45	-10.3791	1.509089
19F-485	-10.3768	1.860055
19F-282	-10.3728	1.895618
19F-1787	-10.3686	1.686649
19F-1790	-10.3655	1.717078
19F-1770	-10.3648	1.665444
19F-374	-10.3648	1.804089
19F-263	-10.3626	1.989244
19F-1102	-10.3615	1.988219
19F-1019	-10.3551	1.087488
19F-1816	-10.3549	2.008186
19F-267	-10.3492	1.207109
19F-1047	-10.3429	2.161741
19F-272	-10.3383	1.149921
19F-1033	-10.3356	0.862364
19F-472	-10.3354	1.83826
19F-1771	-10.3344	1.844557

19F-759	-10.3237	2.006023
19F-1777	-10.3203	1.462945
19F-1024	-10.3171	0.860111
19F-1788	-10.3158	1.917685
19F-1031	-10.3109	0.840154
19F-1062	-10.3064	2.092233
19F-1803	-10.304	1.376586
19F-388	-10.298	2.285286
19F-294	-10.2964	1.897677
19F-516	-10.2865	1.070833
19F-1022	-10.2845	0.792954
19F-1647	-10.2795	2.000863
19F-1030	-10.278	1.39808
19F-1072	-10.2717	2.031742
19F-1063	-10.2717	2.198967
19F-1026	-10.2644	1.046864
19F-262	-10.264	2.265002
19F-358	-10.2632	1.220023
19F-377	-10.2577	2.129365
19F-769	-10.251	1.867994

19F-1812	-10.2504	1.112261
19F-271	-10.2448	2.037626
19F-1776	-10.2432	1.590612
19F-765	-10.2431	2.151265
19F-390	-10.2426	2.234234
19F-500	-10.2385	1.593612
19F-1584	-10.2352	2.277759
19F-1578	-10.2325	1.785342
19F-387	-10.2229	2.158774
19F-360	-10.2195	1.90166
19F-524	-10.2157	1.708704
19F-1769	-10.2149	1.230224
19F-1792	-10.2117	1.508135
19F-1793	-10.2039	1.718873
19F-1035	-10.1925	0.749962
19F-1029	-10.1885	1.335338
19F-389	-10.1831	1.958015
19F-1065	-10.1768	1.997761
19F-211	-10.1691	0.921265
19F-41	-10.169	1.614925
19F-378	-10.1542	1.676703

19F-1798	-10.1487	1.447661
19F-264	-10.1295	1.261814
19F-565	-10.1221	1.374603
19F-770	-10.12	2.140645
19F-754	-10.1103	1.987618
19F-1824	-10.1102	1.729508
19F-43	-10.1065	1.365587
19F-1067	-10.1008	2.352697
19F-49	-10.0956	1.140054
19F-346	-10.062	1.497524
19F-382	-10.0587	1.778111
19F-1802	-10.0491	1.568357
19F-1815	-10.0346	1.555796
19F-937	-10.0313	1.055998
19F-341	-10.0286	1.310168
19F-518	-10.0284	1.60283
19F-1069	-10.0266	2.243578
19F-383	-10.0251	2.020265
19F-219	-10.0203	0.978957
19F-1027	-10.0188	1.263825
19F-507	-10.0151	1.736623
19F-1061	-10.0017	2.157167
19F-279	-9.99263	1.873453

19F-1068	-9.99005	2.169286
19F-1774	-9.97963	1.198661
19F-762	-9.97345	1.785501
19F-1071	-9.96749	1.947177
19F-39	-9.96344	1.563854
19F-1806	-9.95584	1.173516
19F-339	-9.94214	1.485308
19F-380	-9.94	1.603225
19F-1661	-9.93407	1.116405
19F-1804	-9.93291	1.66737
19F-1023	-9.92147	1.411863
19F-561	-9.92102	1.674454
19F-392	-9.921	1.508115
19F-349	-9.90502	1.322028
19F-244	-9.89771	1.283498
19F-936	-9.88323	1.663358
19F-48	-9.87939	1.462964
19F-1809	-9.87577	1.733797
19F-348	-9.87445	0.767737
19F-1070	-9.87327	2.177243
19F-276	-9.87325	1.486684
19F-355	-9.86741	0.952931
19F-764	-9.86505	1.908807

19F-53	-9.86259	1.280779
19F-164	-9.86085	1.423902
19F-1663	-9.84319	1.398265
19F-347	-9.84123	1.132895
19F-287	-9.8322	1.493882
19F-942	-9.81851	1.821393
19F-50	-9.81492	1.029033
19F-1789	-9.79116	1.857882
19F-1783	-9.79038	1.861133
19F-369	-9.7864	1.674252
19F-37	-9.77467	1.80894
19F-761	-9.76602	1.841333
19F-210	-9.73945	1.170718
19F-755	-9.73171	1.642685
19F-1808	-9.72881	1.113467
19F-250	-9.72269	2.000498
19F-359	-9.70694	1.091426
19F-353	-9.70287	1.080146
19F-1103	-9.70276	2.023388
19F-42	-9.69818	1.176235
19F-1662	-9.6937	0.646779
19F-253	-9.68649	1.562787
19F-756	-9.66548	1.956471
19F-768	-9.6558	1.978655

19F-1664	-9.64957	1.185668
19F-1044	-9.60817	1.27895
19F-338	-9.5963	1.000352
19F-47	-9.58826	1.375966
19F-345	-9.58209	1.48928
19F-277	-9.58079	2.150294
19F-46	-9.57702	1.248212
19F-55	-9.57672	1.278908
19F-54	-9.57034	1.266717
19F-352	-9.5668	0.918603
19F-354	-9.55795	1.340384
19F-340	-9.54522	1.158688
19F-44	-9.53596	1.171608
19F-207	-9.50061	1.108673
19F-343	-9.47889	1.106127
19F-281	-9.47257	1.539377
19F-242	-9.46149	0.819876
19F-36	-9.45018	1.707659
19F-246	-9.4247	1.943119
19F-1066	-9.3502	2.040685
19F-52	-9.31812	1.317992
19F-337	-9.30397	0.859859
19F-350	-9.30002	1.268587
19F-342	-9.28138	1.20209
19F-51	-9.22168	1.436498
19F-351	-9.13676	1.368682
19F-38	-9.13206	0.768853

19F-247	-9.05162	1.48571
19F-344	-8.95812	0.654821
19F-216	-8.88613	1.522838
19F-269	-8.59278	0.651854
19F-266	-8.02813	0.910707
19F-224	-5.47717	-2.62124
19F-245	-4.41445	0.736531
19F-1577	-4.16087	0.752017
19F-1673	1.704269	-0.41191
19F-171	1.778096	-7.99647
19F-414	1.977388	-0.61013
19F-172	2.185393	-7.10658
19F-308	2.329028	-0.57201
19F-298	2.329483	-0.56015
19F-303	2.33031	-0.58363
19F-305	2.331289	-0.58891
19F-300	2.331453	-0.58203
19F-410	2.334815	-0.57416
19F-407	2.341742	-0.55954
19F-296	2.343816	-0.57517
19F-418	2.348233	-0.57747
19F-119	2.355047	-0.59824
19F-170	2.381608	-6.30937
19F-175	2.393805	-8.21065
19F-299	2.395808	-0.59768
19F-416	2.40569	-0.5346
19F-415	2.424905	-0.61723
19F-408	2.424905	-0.61723

19F-118	2.428467	-0.61174
19F-306	2.430987	-0.60422
19F-121	2.431621	-0.61795
19F-413	2.43243	-0.61156
19F-297	2.432513	-0.62352
19F-120	2.432513	-0.62352
19F-411	2.433588	-0.61823
19F-409	2.434232	-0.61953
19F-412	2.435871	-0.63735
19F-417	2.448393	-0.62612
19F-304	2.456967	-0.64353
19F-302	2.469431	-0.64198
19F-301	2.474384	-0.61947
19F-122	2.572238	-0.61725
19F-1675	2.741805	-1.08362
19F-1676	3.052087	-1.543
19F-1677	3.182748	-1.67143
19F-1674	3.290531	-0.37289
19F-161	3.514912	-7.73585
19F-174	4.180661	-9.16241
19F-163	4.309516	-9.15208
19F-167	4.328097	-9.58431
19F-169	4.59158	-10.1033
19F-1524	4.81402	-3.47745
19F-1526	4.847681	-4.32486

19F-229	4.907169	-5.96598
19F-1533	4.958622	-4.98668
19F-178	5.009486	-9.85206
19F-1523	5.074336	-4.47967
19F-1534	5.075454	-5.6925
19F-1532	5.078217	-4.79941
19F-1535	5.113509	-5.23536
19F-1519	5.133897	-5.43757
19F-177	5.149688	-9.8666
19F-162	5.170473	-9.42441
19F-1528	5.187512	-4.17337
19F-165	5.193447	-9.65217
19F-184	5.249295	-6.65421
19F-1537	5.321485	-4.35711
19F-1521	5.38208	-4.99862
19F-231	5.416855	-7.67197
19F-1527	5.421038	-5.11436
19F-228	5.545568	-7.60938
19F-1530	5.582216	-5.30129
19F-1525	5.61335	-5.43632

19F-1518	5.720455	-4.55481
19F-234	5.856904	-9.02236
19F-1522	5.857668	-5.50372
19F-239	5.906632	-9.10129
19F-1529	5.931378	-5.37059
19F-182	6.014235	-6.46421
19F-173	6.056978	-9.69643
19F-1531	6.069535	-3.90787
19F-185	6.114862	-9.34017
19F-189	6.121101	-7.91115
19F-180	6.127789	-8.37247
19F-233	6.147018	-7.87626
19F-232	6.164831	-8.46241
19F-1536	6.164896	-5.17215
19F-168	6.194158	-9.5426
19F-237	6.211987	-8.268
19F-226	6.214693	-8.17852
19F-1520	6.244405	-5.69022
19F-225	6.265321	-8.99034
19F-541	6.265434	-5.51818
19F-236	6.333145	-8.86079
19F-190	6.355615	-9.30934
19F-238	6.375475	-7.73326
19F-136	6.378503	-12.1921
19F-132	6.385808	-10.5993

19F-134	6.387516	-11.8127
19F-130	6.393281	-11.6915
19F-131	6.449873	-10.952
19F-1679	6.512394	-5.68351
19F-240	6.513023	-9.07168
19F-125	6.54147	-10.1743
19F-787	6.547981	-8.73887
19F-772	6.588121	-9.11248
19F-181	6.589615	-10.0151
19F-786	6.603253	-9.12969
19F-777	6.60505	-9.2187
19F-179	6.615255	-7.66476
19F-129	6.63406	-10.4538
19F-771	6.634137	-9.03165
19F-775	6.635673	-9.09365
19F-183	6.641474	-9.14914
19F-31	6.657941	-12.012
19F-204	6.672355	-4.75289
19F-196	6.690015	-5.79779
19F-784	6.690068	-8.59059
19F-774	6.691475	-8.47342
19F-779	6.696093	-8.55255
19F-235	6.719767	-9.91978
19F-127	6.725712	-11.444
19F-128	6.786789	-12.0185
19F-133	6.790432	-11.6135
19F-186	6.792518	-9.94677
19F-778	6.826625	-8.22958
19F-230	6.845273	-9.17542

19F-138	6.851002	-12.0014
19F-135	6.851069	-10.9705
19F-123	6.864166	-10.8894
19F-776	6.865362	-9.40064
19F-108	6.879563	-10.2091
19F-785	6.884709	-9.17958
19F-782	6.886741	-8.85715
19F-106	6.89146	-10.2011
19F-788	6.937899	-8.30007
19F-99	6.942532	-10.0875
19F-98	6.951333	-9.95915
19F-205	6.955006	-5.25026
19F-101	6.955511	-10.5694
19F-96	6.967211	-9.75323
19F-137	7.03146	-11.443
19F-104	7.049085	-10.6234
19F-187	7.061302	-8.96681
19F-780	7.066011	-8.53716
19F-166	7.107806	-10.8929
19F-100	7.116263	-9.5084
19F-188	7.116273	-9.35804
19F-102	7.116858	-10.132
19F-152	7.117523	-13.4376
19F-783	7.132619	-8.29201
19F-751	7.140873	-5.5786
19F-97	7.170945	-9.74446
19F-24	7.182296	-13.0584
19F-1718	7.184123	-12.632
19F-150	7.188386	-12.4589

19F-1712	7.196419	-12.4197
19F-146	7.202661	-12.4437
19F-740	7.208944	-5.11178
19F-985	7.227412	-5.3987
19F-148	7.288975	-12.2926
19F-206	7.293831	-5.66648
19F-17	7.301754	-12.5248
19F-753	7.304062	-5.01424
19F-107	7.315625	-9.90872
19F-745	7.332345	-5.28511
19F-741	7.368093	-5.08684
19F-18	7.383267	-12.4502
19F-1713	7.385281	-12.6661
19F-773	7.39522	-8.61106
19F-5	7.403453	-11.5494
19F-984	7.407108	-6.21394
19F-147	7.407271	-12.6913
19F-191	7.426427	-5.77339
19F-21	7.427716	-12.0645
19F-20	7.431387	-12.7851
19F-781	7.43459	-8.68659
19F-149	7.439761	-13.2205
19F-195	7.44132	-5.78955
19F-537	7.489041	-5.66528
19F-151	7.512654	-12.5086
19F-103	7.519211	-10.436
19F-141	7.525946	-12.5903

19F-1717	7.52775	-13.6132
19F-28	7.53532	-12.5875
19F-9	7.536434	-12.4826
19F-14	7.538578	-12.2578
19F-23	7.551572	-12.8452
19F-16	7.571563	-11.9428
19F-540	7.571725	-5.54428
19F-202	7.572968	-5.80428
19F-746	7.575054	-5.63044
19F-153	7.603436	-12.8802
19F-25	7.603709	-13.5033
19F-145	7.609782	-13.5709
19F-546	7.610052	-5.84681
19F-993	7.615264	-5.90679
19F-142	7.622289	-12.7285
19F-992	7.623591	-5.21088
19F-1716	7.627445	-12.9138
19F-176	7.62984	-10.2908
19F-1715	7.63952	-12.3777
19F-139	7.66082	-12.7628
19F-10	7.66966	-11.819
19F-140	7.673502	-13.2611
19F-144	7.686816	-13.1326
19F-201	7.690167	-6.471
19F-199	7.696515	-6.12821
19F-193	7.698506	-6.54789
19F-747	7.701933	-5.46948

19F-544	7.725848	-5.00419
19F-742	7.729709	-5.92297
19F-4	7.750759	-12.835
19F-7	7.751285	-12.6906
19F-34	7.752642	-13.5773
19F-8	7.797864	-12.5191
19F-1714	7.805669	-12.5487
19F-988	7.805985	-5.90358
19F-3	7.817229	-11.7377
19F-983	7.831394	-6.45708
19F-996	7.834665	-6.33771
19F-12	7.847703	-12.4153
19F-198	7.853279	-6.2201
19F-1000	7.870099	-5.98887
19F-998	7.885412	-6.43162
19F-19	7.892689	-13.9186
19F-1680	7.893894	-6.68044
19F-15	7.89401	-12.2428
19F-35	7.908702	-13.6633
19F-200	7.918102	-5.88133
19F-11	7.942581	-12.2199
19F-32	7.944803	-13.2688
19F-991	7.95167	-6.58326
19F-26	7.955132	-12.3059
19F-982	7.965794	-5.87198
19F-203	7.971056	-4.97253
19F-22	8.004878	-13.335

19F-999	8.010101	-6.56501
19F-748	8.013935	-4.72346
19F-143	8.024871	-13.1694
19F-1678	8.032276	-5.20797
19F-543	8.05178	-5.55977
19F-734	8.059837	-6.90681
19F-735	8.078318	-5.72386
19F-743	8.088087	-5.69597
19F-997	8.110137	-5.88426
19F-551	8.120933	-4.60353
19F-6	8.129915	-12.7805
19F-535	8.165778	-6.0945
19F-27	8.16585	-12.6376
19F-1	8.166027	-12.3763
19F-986	8.195914	-6.63339
19F-545	8.199445	-5.89549
19F-994	8.227468	-6.2101
19F-29	8.233962	-13.1178
19F-33	8.238033	-12.5544
19F-736	8.241639	-5.35989
19F-194	8.242342	-6.21566
19F-538	8.246492	-5.20021
19F-549	8.254862	-5.1072
19F-995	8.306835	-6.42114
19F-542	8.330582	-6.22667
19F-197	8.340864	-6.41188
19F-749	8.355858	-5.01006
19F-744	8.388227	-5.66582
19F-547	8.444451	-6.31588

19F-536	8.449798	-5.70684
19F-750	8.457904	-5.58968
19F-987	8.542161	-5.76231
19F-737	8.589083	-5.18905
19F-548	8.61406	-5.25678
19F-192	8.622539	-6.44312
19F-552	8.684057	-5.50113
19F-550	8.704579	-5.1169
19F-739	8.723863	-6.50225
19F-738	8.764487	-5.77493
19F-752	8.775425	-5.82124
19F-989	8.816202	-6.45983
19F-539	9.010416	-6.00276
19F-75	10.8114	4.806932
19F-58	11.48293	6.325933
19F-79	13.02126	6.16276
19F-73	13.72293	6.940487
19F-77	13.85808	6.843433
19F-71	14.69102	8.113762
19F-62	14.75206	8.369933
19F-59	14.91516	7.115681
19F-81	14.96489	7.06263
19F-82	14.96905	7.979477
19F-76	15.19832	7.893108
19F-582	15.27007	7.685008
19F-80	15.37328	7.855596
19F-908	15.38541	8.347996
19F-1709	15.40469	8.469305
19F-310	15.4381	7.680991

19F-318	15.45674	8.585499
19F-1603	15.47264	8.369774
19F-1669	15.49307	8.399123
19F-1670	15.49468	8.420591
19F-1596	15.50979	8.448996
19F-459	15.51692	8.499385
19F-336	15.5322	8.22708
19F-1665	15.54616	8.734944
19F-1671	15.56143	8.449073
19F-1707	15.56463	8.491987
19F-457	15.60007	8.776414
19F-403	15.6067	8.665463
19F-1605	15.61227	8.325649
19F-1708	15.61302	8.549974
19F-1601	15.62232	8.610597
19F-1668	15.62973	8.355548
19F-1572	15.63391	8.925429
19F-1667	15.63709	8.4044
19F-1626	15.6414	8.597761

19F-1710	15.65305	8.489207
19F-1666	15.65736	8.467361
19F-1598	15.66724	8.41632
19F-1597	15.68477	8.826207
19F-1567	15.69715	8.276521
19F-1604	15.70397	8.364408
19F-1711	15.70759	8.480942
19F-316	15.71027	8.557034
19F-1672	15.71079	8.4712
19F-1571	15.71199	8.62536
19F-321	15.72647	8.675799
19F-909	15.77598	8.04759
19F-452	15.77702	8.490193
19F-1600	15.78216	8.933451
19F-558	15.80382	8.631746
19F-1625	15.81615	8.615456
19F-1599	15.81997	8.794378
19F-1606	15.822	8.531331
19F-560	15.82921	8.338207

19F-1595	15.83984	8.680429
19F-317	15.84099	8.733592
19F-573	15.84698	8.481784
19F-1602	15.86508	8.78944
19F-311	15.86881	8.59572
19F-456	15.86965	8.595196
19F-453	15.87346	8.640998
19F-557	15.87529	8.454215
19F-458	15.87574	8.732376
19F-553	15.88254	8.516979
19F-580	15.88971	8.534459
19F-56	15.89105	8.769505
19F-910	15.89954	8.60474
19F-1563	15.89967	8.732722
19F-312	15.91523	8.127573
19F-725	15.93208	8.258593
19F-726	15.93898	8.426997
19F-907	15.94634	8.646946
19F-584	15.96119	8.218804
19F-314	15.96327	8.969298
19F-904	15.96696	8.710773
19F-575	15.98455	8.536849
19F-309	15.9871	8.32974
19F-322	15.99349	8.583105
19F-404	15.99731	8.264299
19F-60	16.00088	8.629852
19F-574	16.00357	8.527948

19F-455	16.00404	8.624584
19F-554	16.00498	8.626928
19F-70	16.01754	8.538715
19F-579	16.01763	8.302382
19F-64	16.02731	8.393197
19F-451	16.03094	8.504653
19F-320	16.03329	8.255116
19F-63	16.03577	8.794497
19F-577	16.03722	8.705825
19F-454	16.04006	8.589767
19F-315	16.04293	8.598725
19F-576	16.04758	8.475168
19F-1564	16.04827	8.76553
19F-583	16.0503	8.563802
19F-66	16.05576	8.597961
19F-905	16.0559	8.734098
19F-1574	16.05673	8.753763
19F-578	16.05805	8.247335
19F-556	16.06464	8.602091
19F-731	16.06925	8.553644
19F-466	16.07124	9.195314
19F-906	16.07352	8.806206
19F-559	16.09942	8.534576
19F-1566	16.10294	8.719108
19F-402	16.10843	8.66215
19F-1575	16.11215	8.686169

19F-733	16.12017	8.45712
19F-581	16.14556	8.426871
19F-555	16.15269	8.648442
19F-57	16.15746	8.517289
19F-911	16.15955	8.661255
19F-728	16.16173	8.752392
19F-1573	16.16371	8.499081
19F-69	16.16581	8.704776
19F-325	16.16906	8.886983
19F-722	16.17312	8.632216
19F-724	16.17466	8.637859
19F-401	16.17769	8.35391
19F-727	16.18498	8.546219
19F-72	16.19156	8.333688
19F-469	16.20098	8.91534
19F-319	16.206	8.558892
19F-467	16.20794	8.833504
19F-67	16.21124	8.766548
19F-394	16.21369	8.614768
19F-1570	16.22096	8.877321
19F-68	16.22207	8.779454
19F-732	16.2285	8.446188
19F-397	16.2295	8.88962
19F-730	16.2325	8.495425
19F-406	16.23978	8.865418
19F-405	16.24533	8.571419
19F-1569	16.24547	8.759613

19F-335	16.25771	8.693155
19F-327	16.26127	8.922087
19F-729	16.26867	8.376946
19F-1565	16.27337	8.964777
19F-723	16.28636	8.551611
19F-463	16.29705	8.815472
19F-462	16.30069	8.927807
19F-331	16.30325	8.911237
19F-329	16.30765	8.819247
19F-333	16.34538	8.736115
19F-398	16.36474	8.892093
19F-461	16.36917	8.978051
19F-330	16.371	8.77352
19F-326	16.37301	8.921704
19F-313	16.3885	8.935047
19F-399	16.39352	8.972668
19F-328	16.39784	8.886217
19F-395	16.39842	8.592838
19F-332	16.41983	8.990739
19F-396	16.43096	8.84544
19F-400	16.44452	8.708542
19F-1568	16.4634	8.795846
19F-460	16.4762	8.667031
19F-61	16.47775	8.938712
19F-334	16.48646	8.852758
19F-468	16.51799	8.991725
19F-465	16.6366	8.958987

Table S2 – Sheet 7: *Linum lewisii*

id	V1	V2
19F-297	-14.7984	-0.03025
19F-304	-14.7931	-0.04183
19F-119	-14.7931	-0.04183
19F-121	-14.7931	-0.04183
19F-122	-14.7931	-0.04183
19F-413	-14.7931	-0.04183
19F-409	-14.7931	-0.04183
19F-411	-14.7931	-0.04183
19F-418	-14.7422	0.419252
19F-305	-14.7417	0.42296
19F-301	-14.7417	0.42296
19F-407	-14.7417	0.42296
19F-120	-14.688	-0.07383
19F-417	-14.6827	-0.08541
19F-414	-14.6796	0.003232
19F-415	-14.6796	0.003232
19F-408	-14.6796	0.003232
19F-416	-14.6796	0.003232
19F-302	-14.6794	-0.02953
19F-412	-14.6788	-0.04554
19F-306	-14.6734	-0.06103
19F-118	-14.6721	-0.04679
19F-410	-14.6335	0.479591
19F-303	-14.6227	0.423681
19F-300	-14.6207	0.417996
19F-296	-14.5103	0.374418
19F-308	-14.5007	0.414422

19F-299	-14.3172	0.103037
19F-298	-14.163	0.455963
19F-1577	-2.00416	1.252296
19F-58	-1.48086	-1.43693
19F-75	-1.30143	-0.98235
19F-73	-0.76325	-1.57664
19F-77	-0.17605	-0.58274
19F-266	0.284943	2.877459
19F-281	0.525354	1.733418
19F-82	0.768083	-0.72802
19F-76	0.833216	-1.10477
19F-79	0.861653	-0.80411
19F-56	0.89335	-1.67639
19F-576	0.910231	-1.68759
19F-269	0.941762	2.363642
19F-62	0.985029	-0.73363
19F-582	1.16402	-1.70277
19F-1603	1.186736	-1.22221
19F-289	1.212371	2.384133
19F-579	1.272247	-1.70972
19F-583	1.272247	-1.70972
19F-580	1.28042	-1.71075
19F-80	1.318938	-1.14775
19F-1575	1.367596	-1.70425
19F-1569	1.374538	-1.6937
19F-64	1.376511	-1.61489

19F-59	1.383949	-1.7061
19F-732	1.384102	-1.70488
19F-574	1.392919	-1.71709
19F-318	1.488268	-1.71162
19F-315	1.492328	-1.71183
19F-560	1.492328	-1.71183
19F-729	1.492328	-1.71183
19F-1601	1.496345	-1.71274
19F-72	1.496578	-1.71241
19F-558	1.500406	-1.71294
19F-401	1.500406	-1.71294
19F-722	1.500508	-1.71265
19F-1564	1.501244	-1.62247
19F-1605	1.512659	-1.71507
19F-577	1.513571	-1.72605
19F-584	1.522506	-1.63511
19F-578	1.522506	-1.63511
19F-81	1.529265	-1.41233
19F-267	1.536899	2.326842
19F-1067	1.541594	2.366243
19F-909	1.556698	-1.24548
19F-292	1.603882	2.817854
19F-571	1.605383	1.918104
19F-66	1.608939	-1.719
19F-60	1.608939	-1.719
19F-553	1.608939	-1.719

19F-1597	1.608939	-1.719
19F-460	1.60946	-1.71804
19F-310	1.612365	-0.78031
19F-555	1.613	-1.71921
19F-726	1.613	-1.71921
19F-309	1.613101	-1.71888
19F-321	1.617855	-1.62964
19F-454	1.618532	-1.72138
19F-319	1.621077	-1.72032
19F-1606	1.621385	-1.71943
19F-573	1.634262	-1.73185
19F-581	1.634262	-1.73185
19F-575	1.634262	-1.73185
19F-294	1.638814	2.024291
19F-1598	1.653278	-1.7222
19F-287	1.707322	2.701045
19F-282	1.711588	2.701433
19F-67	1.729611	-1.72638
19F-69	1.729611	-1.72638
19F-71	1.729611	-1.72638
19F-557	1.729611	-1.72638
19F-465	1.729611	-1.72638
19F-556	1.729611	-1.72638
19F-459	1.729611	-1.72638
19F-1565	1.729611	-1.72638
19F-908	1.729611	-1.72638

19F-320	1.733672	-1.72658
19F-313	1.733672	-1.72658
19F-317	1.733672	-1.72658
19F-404	1.733672	-1.72658
19F-724	1.733672	-1.72658
19F-723	1.733672	-1.72658
19F-1566	1.737689	-1.72749
19F-733	1.737791	-1.7272
19F-906	1.737791	-1.7272
19F-335	1.73807	-1.7314
19F-312	1.738527	-1.63702
19F-263	1.740219	2.905208
19F-70	1.742057	-1.72681
19F-1625	1.742057	-1.72681
19F-1572	1.742057	-1.72681
19F-725	1.745869	-1.72831
19F-1061	1.74698	2.017117
19F-1594	1.748399	2.904391
19F-475	1.754127	2.028261
19F-1596	1.75905	-1.63856
19F-1062	1.768126	2.125344
19F-462	1.770695	-1.87243
19F-1563	1.781365	-1.25236
19F-261	1.799286	2.42002

19F-1580	1.815091	2.704273
19F-274	1.815185	2.515079
19F-473	1.83226	2.694057
19F-277	1.832911	2.802665
19F-295	1.845225	2.8031
19F-271	1.848445	2.89826
19F-311	1.850283	-1.73375
19F-314	1.850283	-1.73375
19F-61	1.850283	-1.73375
19F-63	1.850283	-1.73375
19F-333	1.850283	-1.73375
19F-452	1.850283	-1.73375
19F-559	1.850283	-1.73375
19F-406	1.850283	-1.73375
19F-455	1.850283	-1.73375
19F-463	1.850283	-1.73375
19F-403	1.850283	-1.73375
19F-397	1.850283	-1.73375
19F-1574	1.850283	-1.73375
19F-1567	1.850283	-1.73375
19F-1568	1.850283	-1.73375
19F-1626	1.850283	-1.73375
19F-731	1.850283	-1.73375
19F-331	1.850453	-1.73597
19F-469	1.850588	-1.73528
19F-400	1.851565	-1.73534

19F-1602	1.851565	-1.73534
19F-1571	1.858361	-1.73487
19F-1573	1.858463	-1.73457
19F-911	1.858463	-1.73457
19F-730	1.858463	-1.73457
19F-57	1.859199	-1.6444
19F-1599	1.874099	-1.73542
19F-336	1.901685	-1.26897
19F-264	1.903609	2.344113
19F-569	1.903609	2.344113
19F-1068	1.903609	2.344113
19F-477	1.906389	2.354573
19F-907	1.911372	-1.17632
19F-1592	1.916575	2.453157
19F-272	1.940486	2.687108
19F-483	1.940486	2.687108
19F-566	1.944648	2.68723
19F-285	1.953452	2.796152
19F-1589	1.953452	2.796152
19F-276	1.957575	2.775437
19F-402	1.965657	-1.72956
19F-457	1.965657	-1.72956
19F-262	1.969117	2.890884
19F-322	1.970955	-1.74113

19F-316	1.970955	-1.74113
19F-325	1.970955	-1.74113
19F-326	1.970955	-1.74113
19F-334	1.970955	-1.74113
19F-461	1.970955	-1.74113
19F-327	1.970955	-1.74113
19F-328	1.970955	-1.74113
19F-329	1.970955	-1.74113
19F-330	1.970955	-1.74113
19F-554	1.970955	-1.74113
19F-466	1.970955	-1.74113
19F-396	1.970955	-1.74113
19F-467	1.970955	-1.74113
19F-332	1.970955	-1.74113
19F-405	1.970955	-1.74113
19F-451	1.970955	-1.74113
19F-468	1.970955	-1.74113
19F-910	1.970955	-1.74113
19F-1600	1.970955	-1.74113
19F-727	1.970955	-1.74113
19F-728	1.970955	-1.74113
19F-1570	1.970955	-1.74113
19F-1595	1.970955	-1.74113
19F-1604	1.970955	-1.74113
19F-68	1.979871	-1.65177
19F-399	1.979871	-1.65177

19F-394	1.979871	-1.65177
19F-395	1.979871	-1.65177
19F-293	1.981562	2.890455
19F-565	1.988324	2.002364
19F-1072	1.988324	2.002364
19F-1065	1.988324	2.002364
19F-1579	1.988324	2.002364
19F-1066	1.988324	2.002364
19F-1588	1.988324	2.002364
19F-1064	1.995991	2.12298
19F-1069	2.00129	2.111408
19F-1063	2.018983	2.348309
19F-453	2.021916	-1.28005
19F-456	2.021916	-1.28005
19F-458	2.021916	-1.28005
19F-904	2.021916	-1.28005
19F-905	2.021916	-1.28005
19F-482	2.024281	2.336736
19F-568	2.025563	2.335146
19F-1583	2.028443	2.336858
19F-398	2.030831	-1.19069
19F-485	2.031949	2.457353
19F-279	2.032359	2.335623

19F-1070	2.032359	2.335623
19F-286	2.033197	2.426095
19F-283	2.036727	2.336308
19F-562	2.037247	2.44578
19F-1576	2.037247	2.44578
19F-1071	2.037247	2.44578
19F-484	2.05586	2.691304
19F-290	2.061158	2.679732
19F-275	2.061158	2.679732
19F-567	2.061158	2.679732
19F-480	2.061158	2.679732
19F-471	2.061679	2.68069
19F-268	2.070074	2.76909
19F-1591	2.073603	2.679303
19F-1587	2.073603	2.679303
19F-1582	2.074124	2.788775
19F-1593	2.074124	2.788775
19F-487	2.082304	2.787958
19F-278	2.089789	2.883507
19F-1590	2.089789	2.883507
19F-479	2.130552	2.218939
19F-1578	2.130552	2.218939
19F-288	2.144953	2.32936

19F-284	2.144953	2.32936
19F-561	2.144953	2.32936
19F-563	2.144953	2.32936
19F-564	2.144953	2.32936
19F-476	2.144953	2.32936
19F-572	2.144953	2.32936
19F-1585	2.144953	2.32936
19F-1586	2.144953	2.32936
19F-1581	2.157762	2.201656
19F-481	2.157919	2.438404
19F-470	2.170728	2.3107
19F-280	2.18183	2.672355
19F-486	2.18183	2.672355
19F-478	2.18183	2.672355
19F-488	2.18183	2.672355
19F-570	2.18183	2.672355
19F-474	2.18183	2.672355
19F-291	2.190746	2.761714
19F-472	2.194796	2.781399
19F-270	2.210461	2.87613
19F-273	2.210461	2.87613
19F-1584	2.210461	2.87613

Table S2 – Sheet 8: *Linum perenne*

id	V1	V2
19F-525	-11.834	4.812185
19F-528	-11.7245	5.130996
19F-1013	-11.6798	4.360655
19F-522	-11.6203	5.208066
19F-526	-11.5995	4.912408
19F-1052	-11.5837	4.243043
19F-1053	-11.5574	4.937963
19F-521	-11.502	6.064815
19F-519	-11.4945	5.194911
19F-1042	-11.4743	3.606585
19F-517	-11.4713	5.620908
19F-523	-11.469	3.79943
19F-1001	-11.4539	4.051146
19F-385	-11.4086	5.332158
19F-1050	-11.4067	5.386209
19F-529	-11.3954	4.471654
19F-946	-11.3931	4.358187
19F-534	-11.386	5.05664
19F-1046	-11.3749	3.571664
19F-1007	-11.3646	4.179955
19F-367	-11.3527	4.005336
19F-533	-11.3424	5.110434
19F-372	-11.327	6.434399
19F-1043	-11.3208	3.796501
19F-1039	-11.2971	4.115909
19F-1010	-11.2958	6.356044
19F-1105	-11.2918	2.799518

19F-1017	-11.2671	3.543623
19F-1100	-11.2662	3.686296
19F-363	-11.2634	3.959285
19F-530	-11.257	5.213668
19F-1016	-11.2516	5.353149
19F-1098	-11.2475	5.257276
19F-941	-11.2453	4.320643
19F-1006	-11.2285	5.649229
19F-520	-11.2254	4.960642
19F-1106	-11.2173	4.224249
19F-932	-11.2156	4.188559
19F-362	-11.2148	3.642372
19F-376	-11.1641	3.604687
19F-532	-11.1598	5.107719
19F-1095	-11.1573	5.328977
19F-1051	-11.1521	3.378503
19F-358	-11.1401	6.1223
19F-366	-11.1401	4.090441
19F-1094	-11.1396	4.598185
19F-387	-11.1353	6.094457
19F-527	-11.1352	5.663878
19F-1810	-11.1286	-3.19988
19F-364	-11.1224	3.861336
19F-1813	-11.1003	-2.23778
19F-381	-11.1	4.921249
19F-377	-11.0958	2.114965
19F-1004	-11.0957	5.752733
19F-1101	-11.09	3.238373
19F-370	-11.0786	3.972841

19F-947	-11.0726	4.050449
19F-1817	-11.0704	-2.35005
19F-1009	-11.0681	4.30062
19F-1003	-11.061	2.633961
19F-390	-11.0462	4.761384
19F-1048	-11.0437	4.935423
19F-934	-11.0426	5.103281
19F-1096	-11.0388	4.190288
19F-943	-11.0326	2.86756
19F-361	-11.0188	5.257095
19F-386	-10.9971	5.094519
19F-375	-10.9758	5.556379
19F-393	-10.9584	5.178802
19F-1012	-10.938	3.004701
19F-515	-10.9374	5.610005
19F-1005	-10.9277	4.218699
19F-1049	-10.9257	1.458059
19F-935	-10.9187	2.410559
19F-938	-10.914	5.836705
19F-1041	-10.9105	3.560979
19F-1045	-10.887	3.758223
19F-379	-10.8868	3.716727
19F-391	-10.866	2.876824
19F-933	-10.8641	3.27568
19F-1008	-10.8503	2.554134
19F-1104	-10.8468	4.928252
19F-1014	-10.839	3.586952
19F-356	-10.8285	3.7361
19F-1015	-10.8208	3.951798

19F-940	-10.8205	3.605995
19F-378	-10.8034	3.446475
19F-383	-10.8015	4.329605
19F-1107	-10.7758	5.296214
19F-949	-10.7706	3.131185
19F-1791	-10.7599	-2.91063
19F-1823	-10.7539	-3.86513
19F-945	-10.7518	4.396976
19F-368	-10.7453	5.063102
19F-365	-10.7178	5.39346
19F-384	-10.6942	5.114698
19F-357	-10.6905	4.991232
19F-371	-10.6702	5.294674
19F-939	-10.6681	2.334262
19F-1782	-10.6642	-2.41045
19F-1040	-10.6317	4.084565
19F-948	-10.6267	3.016482
19F-1011	-10.6248	2.819072
19F-1047	-10.6142	4.377098
19F-1800	-10.5973	-4.40483
19F-516	-10.5851	6.284581
19F-1110	-10.5693	3.365675
19F-1785	-10.5495	-2.87801
19F-1787	-10.5324	-2.27782
19F-1779	-10.524	-2.55986
19F-1102	-10.5216	5.039047
19F-524	-10.5109	3.564028
19F-388	-10.5018	3.062342
19F-1826	-10.4956	-2.42069

19F-1825	-10.4948	-2.01252
19F-1002	-10.4736	3.480866
19F-1790	-10.4695	-2.51528
19F-1108	-10.4632	3.387002
19F-1789	-10.4416	-2.95213
19F-1805	-10.4381	-4.44451
19F-1821	-10.4365	-3.44757
19F-1781	-10.4259	-1.61497
19F-1788	-10.4183	-2.67908
19F-1811	-10.378	-3.62426
19F-501	-10.3762	-3.25108
19F-380	-10.3746	3.674126
19F-1783	-10.3492	-3.14083
19F-1799	-10.347	-5.54126
19F-360	-10.3447	5.196094
19F-942	-10.34	5.814926
19F-373	-10.3298	2.832487
19F-518	-10.3225	5.680367
19F-1822	-10.315	-4.06083
19F-499	-10.3051	-3.96199
19F-1097	-10.3047	2.3575
19F-389	-10.2809	4.981945
19F-1786	-10.2764	-2.04554
19F-1792	-10.2692	-5.06961
19F-1820	-10.2656	-3.54572
19F-1797	-10.2642	-5.57488
19F-1099	-10.2299	2.977679
19F-500	-10.2151	-3.2502
19F-512	-10.2036	-3.18785

19F-1795	-10.2022	-7.24492
19F-359	-10.1746	5.981113
19F-1794	-10.1724	-4.30544
19F-511	-10.1663	-3.58546
19F-1780	-10.1625	-1.93583
19F-1778	-10.1571	-1.79137
19F-1109	-10.1353	3.839598
19F-1801	-10.1302	-3.53754
19F-1784	-10.1249	-1.94909
19F-1649	-10.1074	-3.00626
19F-937	-10.0853	1.579016
19F-1647	-10.0822	-3.28145
19F-1818	-10.0696	-4.05881
19F-508	-10.0621	-3.67855
19F-514	-9.97117	-4.59527
19F-509	-9.96609	-3.88809
19F-1819	-9.94274	-1.33125
19F-510	-9.92991	-5.06772
19F-936	-9.92119	5.454183
19F-1814	-9.90636	-3.32019
19F-506	-9.90357	-5.18553
19F-1798	-9.90196	-5.92893
19F-369	-9.90164	5.738288
19F-382	-9.89588	5.730321
19F-1796	-9.87997	-5.08438
19F-1793	-9.80662	-6.48928
19F-1816	-9.75431	-2.99046
19F-502	-9.75384	-5.70455
19F-1804	-9.73696	-4.47354

19F-503	-9.72092	-4.75893
19F-1807	-9.71986	-5.39586
19F-505	-9.64951	-5.51345
19F-1815	-9.62273	-3.78947
19F-374	-9.61297	4.544059
19F-504	-9.57875	-6.09497
19F-754	-9.54045	-9.91935
19F-760	-9.53654	-9.46505
19F-1802	-9.49061	-5.37186
19F-1103	-9.48877	4.385099
19F-759	-9.45391	-9.32389
19F-507	-9.34715	-4.88834
19F-766	-9.33307	-9.72362
19F-757	-9.30541	-9.57531
19F-1044	-9.26257	5.525829
19F-392	-9.25948	3.39273
19F-1812	-9.23013	-5.00608
19F-769	-9.19213	-9.87237
19F-1806	-9.16886	-5.99338
19F-763	-9.15344	-9.73359
19F-1803	-9.12237	-5.56257
19F-765	-9.12153	-10.069
19F-513	-9.07561	-4.85252
19F-341	-8.98305	-7.33364
19F-756	-8.97916	-10.5352
19F-1809	-8.97886	-3.81513
19F-1824	-8.96479	-3.89396
19F-345	-8.96275	-7.32397
19F-339	-8.92331	-7.50586

19F-770	-8.80355	-9.63182
19F-768	-8.77643	-7.3038
19F-758	-8.74271	-9.38577
19F-761	-8.69452	-9.65772
19F-755	-8.6598	-9.83363
19F-352	-8.65473	-7.21545
19F-762	-8.62375	-9.79622
19F-764	-8.55912	-10.1158
19F-1808	-8.50136	-5.6761
19F-346	-8.47689	-7.13453
19F-353	-8.25931	-7.80762
19F-343	-8.18114	-7.96126
19F-350	-8.03996	-5.94569
19F-347	-8.00919	-7.56695
19F-337	-7.95291	-5.02304
19F-349	-7.9315	-7.80056
19F-340	-7.75726	-7.48548
19F-355	-7.5992	-7.29855
19F-348	-7.43758	-7.63042
19F-354	-7.40002	-7.56424
19F-342	-7.33382	-7.00517
19F-338	-7.18051	-5.8579
19F-351	-7.07379	-7.29465
19F-344	-6.91488	-6.60471
19F-1673	9.9854	-2.34781
19F-1675	12.15641	-2.45629
19F-1677	13.43312	-2.7157
19F-1674	13.46116	-1.85637
19F-1676	13.48068	-2.07815

19F-1524	19.77988	-0.12764
19F-1519	22.849	-1.35255
19F-1523	23.04484	-2.32478
19F-1525	23.38564	-1.67544
19F-1533	23.3909	-1.91098
19F-1532	23.56066	-1.27549
19F-1526	23.85059	-0.60822
19F-1535	24.03299	-1.82629
19F-1537	24.09152	-0.9364
19F-1531	24.10427	-1.16221
19F-1528	24.11788	-1.54242
19F-1521	24.27273	-1.66341
19F-1534	24.49112	-2.37999
19F-1522	24.80394	-1.62552
19F-1520	24.85907	-0.73475
19F-1536	25.06649	-0.08438
19F-1530	25.26169	-1.42593
19F-1527	25.26849	-1.90629
19F-1518	25.8963	-1.08874
19F-1529	25.98317	-1.89474
19F-1679	26.3307	1.099167
19F-541	27.38398	-0.09433
19F-751	28.55376	0.768646
19F-753	28.69913	0.7531
19F-741	28.70332	0.083346
19F-746	28.81811	-0.26537
19F-982	28.89947	0.871391
19F-745	28.90013	0.723603
19F-747	28.94249	1.451356

19F-537	28.94712	0.783398
19F-536	28.9606	0.764095
19F-748	28.96412	0.726783
19F-540	28.99489	0.246097
19F-1678	29.00762	1.269076
19F-551	29.15057	0.605928
19F-544	29.18701	0.96306
19F-992	29.19852	0.74228
19F-535	29.2475	2.021721
19F-543	29.26651	0.4494
19F-740	29.35899	0.190061
19F-744	29.36192	0.687409
19F-538	29.37836	1.909507
19F-735	29.39365	0.740221
19F-545	29.43242	1.06201
19F-737	29.44704	0.84518
19F-985	29.53939	0.55079
19F-743	29.60023	0.268039
19F-546	29.83394	0.119839
19F-734	30.08331	0.605783
19F-752	30.13975	0.936483
19F-738	30.14545	1.172644
19F-736	30.16705	0.582883
19F-749	30.2461	0.951718
19F-547	30.43173	1.982679
19F-993	30.43574	1.53033
19F-983	30.43909	1.42507
19F-997	30.49596	0.628406
19F-750	30.50875	1.58073

19F-984	30.5791	0.885213
19F-552	30.58386	1.298252
19F-549	30.59815	0.491098
19F-1680	30.63805	0.853683
19F-550	30.74086	0.865954
19F-542	30.8368	0.870235
19F-987	30.87111	0.910323
19F-986	30.87382	1.433049
19F-988	30.97803	1.073189
19F-742	31.01578	1.766125
19F-739	31.03583	1.242654
19F-539	31.1627	0.763316
19F-548	31.28996	0.852843
19F-994	31.4378	0.820805
19F-996	31.44082	0.995287
19F-989	31.46649	1.376266
19F-1000	31.52255	0.977164
19F-991	31.70029	1.038898
19F-998	31.71519	0.578498
19F-995	31.78889	0.895302
19F-999	32.0172	1.317852

Table S2 – Sheet 9: *Linum austriacum*

id	V1	V2
19F-249	-16.7454	1.362833
19F-243	-16.0712	1.196646
19F-248	-15.9498	1.506889
19F-254	-15.904	1.527866
19F-258	-15.8766	1.352889
19F-1021	-15.7912	1.518009
19F-260	-15.6961	1.682075
19F-212	-15.6453	1.551614
19F-256	-15.5874	1.467046
19F-251	-15.5734	1.450275
19F-257	-15.5656	1.281403
19F-209	-15.5297	2.109776
19F-1025	-15.4326	1.738094
19F-45	-15.4284	0.902819
19F-222	-15.3766	1.934657
19F-1028	-15.3149	1.263009
19F-1032	-15.2945	1.61851
19F-1773	-15.2738	1.643543
19F-43	-15.2702	1.02778
19F-217	-15.2603	1.417227
19F-39	-15.2592	0.909012
19F-41	-15.2543	0.971437
19F-252	-15.2255	1.098983
19F-221	-15.2253	1.789996
19F-220	-15.2126	1.781497
19F-208	-15.1873	1.575828
19F-223	-15.1767	1.385363

19F-55	-15.1698	0.947946
19F-214	-15.1526	1.674341
19F-164	-15.1135	0.692195
19F-213	-15.0656	1.309437
19F-241	-15.0441	1.708433
19F-53	-15.0221	0.995458
19F-1019	-15.0193	1.659843
19F-1030	-15.0171	1.671472
19F-1031	-15.0166	1.562219
19F-1034	-14.992	1.355243
19F-255	-14.9581	1.330346
19F-1022	-14.9571	1.044285
19F-47	-14.9549	0.653123
19F-259	-14.8985	1.378501
19F-49	-14.8964	0.905961
19F-211	-14.8919	1.405053
19F-1035	-14.8385	1.581979
19F-215	-14.8259	1.669306
19F-1018	-14.8115	1.500823
19F-1020	-14.7659	1.415227
19F-46	-14.72	1.269434
19F-1023	-14.713	1.59154
19F-1027	-14.7054	1.402874
19F-1024	-14.7024	1.440779
19F-54	-14.7005	0.973171
19F-1036	-14.6952	1.408316
19F-50	-14.6905	0.930855
19F-44	-14.6837	1.072259
19F-1026	-14.6719	1.334578

19F-48	-14.6704	0.607435
19F-36	-14.6291	0.518884
19F-250	-14.6202	1.696605
19F-37	-14.6057	0.693886
19F-1777	-14.6036	1.374917
19F-1770	-14.6013	1.13907
19F-1033	-14.5931	1.462315
19F-1771	-14.5597	1.459656
19F-218	-14.5593	1.884999
19F-1772	-14.5461	1.468867
19F-42	-14.4403	0.534377
19F-1776	-14.436	1.659097
19F-210	-14.374	1.642159
19F-1664	-14.3738	0.968349
19F-219	-14.3537	1.355509
19F-244	-14.3141	0.983093
19F-1029	-14.3027	1.463294
19F-242	-14.2755	1.270218
19F-52	-14.2468	1.305829
19F-253	-14.1984	1.165617
19F-1661	-14.1946	1.36327
19F-1775	-14.151	1.511163
19F-1663	-14.1213	1.161693
19F-1769	-14.1162	1.339221
19F-207	-14.0849	1.713607
19F-246	-14.0433	0.857527
19F-247	-14.0237	0.649305
19F-38	-13.9836	1.380682
19F-51	-13.9174	0.873459

19F-1662	-13.5271	0.90108
19F-216	-13.5206	1.154334
19F-1774	-13.3408	1.07445
19F-245	-9.4104	-0.26886
19F-224	-7.78231	-2.58364
19F-170	1.82843	2.510413
19F-204	2.580958	-5.38386
19F-172	3.011518	4.558782
19F-205	3.071823	-5.75345
19F-206	3.325507	-6.5256
19F-203	3.406324	-7.08055
19F-1665	3.409435	-8.13823
19F-196	3.476454	-5.30576
19F-1710	3.478214	-8.33836
19F-1666	3.48195	-8.32901
19F-1708	3.493067	-8.34321
19F-1711	3.512367	-8.32399
19F-1709	3.561159	-8.07519
19F-1668	3.566166	-8.15608
19F-1707	3.57163	-8.41585
19F-198	3.593367	-5.9315
19F-1671	3.639809	-8.14671
19F-1670	3.64971	-8.13162
19F-1669	3.662393	-8.33896
19F-1667	3.761633	-8.27993
19F-1672	3.788779	-8.19979
19F-175	3.800549	4.314707
19F-195	3.806291	-6.78745
19F-202	3.869636	-7.02095

19F-193	3.889428	-6.09129
19F-171	3.897632	5.551877
19F-191	3.94473	-6.53005
19F-200	3.984481	-6.06752
19F-229	3.994105	-7.71794
19F-199	4.063869	-6.11533
19F-161	4.208326	3.333272
19F-201	4.215318	-6.23977
19F-194	4.341557	-6.18761
19F-184	4.623118	-8.24203
19F-197	4.653536	-6.36129
19F-192	4.847271	-6.72244
19F-231	4.869807	-7.79015
19F-228	4.992906	-8.46573
19F-182	5.171035	-8.82286
19F-226	5.268392	-9.77027
19F-189	5.686977	-8.21076
19F-167	5.789956	4.789597
19F-237	5.825835	-9.59521
19F-233	5.914249	-8.887
19F-163	6.105814	5.278111
19F-238	6.183976	-9.12072
19F-179	6.210416	-9.80902
19F-234	6.210925	-10.6996
19F-239	6.240084	-10.3312
19F-169	6.247467	6.365638
19F-232	6.252154	-9.11457
19F-180	6.280111	-8.91386
19F-185	6.333907	-9.40794

19F-779	6.498349	-10.9628
19F-225	6.502232	-10.4895
19F-230	6.529567	-10.2496
19F-778	6.552594	-12.0315
19F-236	6.558853	-9.86344
19F-240	6.560599	-9.93762
19F-788	6.578896	-11.2499
19F-174	6.635664	4.816848
19F-190	6.727648	-9.93705
19F-771	6.755727	-11.3185
19F-787	6.769199	-11.1715
19F-162	6.793954	4.883493
19F-776	6.8231	-11.0799
19F-783	6.857547	-11.3841
19F-780	6.859853	-11.4729
19F-187	6.916951	-10.0796
19F-786	6.958319	-11.6095
19F-772	7.017397	-11.2086
19F-774	7.027994	-11.1564
19F-183	7.043137	-10.1623
19F-785	7.04726	-10.945
19F-178	7.052718	5.209998
19F-784	7.067653	-11.4772
19F-165	7.069114	5.636772
19F-777	7.073199	-10.8512
19F-235	7.14904	-10.3563
19F-773	7.150842	-12.4302
19F-188	7.153881	-9.82038
19F-775	7.177075	-11.8397

19F-781	7.182394	-12.3488
19F-782	7.183536	-11.8307
19F-181	7.380352	-10.1489
19F-177	7.567126	4.992136
19F-186	7.623017	-9.95576
19F-173	7.685656	3.998954
19F-100	8.293054	3.307444
19F-108	8.375809	3.426992
19F-168	8.388267	5.197662
19F-125	8.463146	4.30013
19F-98	8.529219	3.466327
19F-106	8.534933	3.836947
19F-97	8.609585	3.190929
19F-107	8.610059	3.173307
19F-129	8.643056	4.753846
19F-99	8.71985	3.629846
19F-104	8.73552	3.289827
19F-96	8.78499	3.356305
19F-101	8.808962	3.540755
19F-102	8.891746	3.063821
19F-103	8.949089	3.429096
19F-131	9.001613	4.950144
19F-136	9.123618	5.437795
19F-132	9.214548	5.574792
19F-176	9.287154	4.546578
19F-135	9.343345	5.196106
19F-123	9.395751	5.411065
19F-166	9.514784	5.133895
19F-134	9.5692	5.829631

19F-31	9.635225	7.148608
19F-138	9.70523	5.602839
19F-127	9.72505	5.878376
19F-133	9.737934	5.428748
19F-130	9.811036	6.021393
19F-5	9.85467	5.443542
19F-128	9.877043	6.405153
19F-16	9.898054	5.884945
19F-17	9.934896	4.790996
19F-137	9.976577	5.866197
19F-21	10.23032	6.40065
19F-1718	10.24915	6.270392
19F-1716	10.34261	6.360195
19F-1713	10.4352	6.761519
19F-10	10.45073	5.929765
19F-1712	10.46489	6.353941
19F-14	10.49403	6.222952
19F-146	10.58036	6.53133
19F-18	10.58309	6.916456
19F-12	10.59034	6.140631
19F-4	10.59259	6.175617
19F-3	10.59883	5.321727
19F-11	10.61505	6.327481
19F-9	10.66037	6.312192
19F-24	10.68999	7.360933
19F-7	10.69886	6.063113
19F-28	10.73223	7.379081
19F-15	10.74202	5.681497
19F-1	10.74522	6.067308

19F-8	10.79925	6.149478
19F-150	10.81523	7.135966
19F-152	10.83833	6.762848
19F-147	10.89548	6.700188
19F-148	10.91002	6.852918
19F-139	10.94918	5.833585
19F-23	10.96086	7.25547
19F-20	10.96209	6.952391
19F-1714	10.96252	6.523035
19F-141	10.96929	6.732931
19F-6	10.98448	6.198505
19F-149	10.99082	7.119913
19F-151	10.99086	6.511578
19F-1715	11.00366	6.630582
19F-26	11.01134	7.016679
19F-153	11.0417	6.690534
19F-1717	11.0688	7.367447
19F-140	11.12913	6.752604
19F-27	11.16523	7.03808
19F-144	11.16549	6.832028
19F-142	11.17614	6.260318
19F-25	11.18925	7.591532
19F-145	11.20696	7.229028
19F-33	11.2156	7.038182
19F-32	11.43161	7.730515
19F-143	11.48042	6.911452
19F-29	11.55944	7.226443
19F-34	11.56393	7.470999
19F-19	11.69687	6.996209

19F-35	11.8062	7.592266
19F-22	11.83904	7.422438
